# KATMAP infers splicing factor activity and regulatory targets from knockdown data

**DOI:** 10.1038/s41587-025-02881-9

**Published:** 2025-11-04

**Authors:** Michael P. McGurk, David C. McWatters, Christopher B. Burge

**Affiliations:** Department of Biology, Massachusetts Institute of Technology, Cambridge, MA US

**Keywords:** Computational models, Software, Transcriptomics

## Abstract

Typical RNA sequencing (RNA-seq) experiments uncover hundreds of splicing changes, reflecting underlying changes in splicing factor (SF) activity. Understanding how SF activity influences transcriptomic variation requires elucidating how each SF impacts splicing. Here, we present an interpretable regression model, KATMAP, which models splicing changes throughout the transcriptome by analyzing changes in SF binding and the resulting alterations in RNA processing. To learn a regulatory model, KATMAP requires SF perturbation RNA-seq data and the SF’s binding motif as inputs, returning a description of the SF’s position-specific regulatory activity and predicted targets. The KATMAP software includes models pretrained on ENCODE SF knockdown data. Learned KATMAP models can be applied to predict SF regulation and *cis*-elements at individual exons, which can guide the design of splice-switching antisense oligonucleotides. KATMAP can also interpret RNA-seq data by uncovering the factors responsible for transcriptomic changes, distinguishing direct SF targets from indirect effects and inferring relevant SFs from clinical RNA-seq data.

## Abstract (web summary)

A biophysical model uses knockdown or overexpression data to infer splicing factor activity.

## Main

Programs of transcriptional and post-transcriptional regulation are responsible for development, physiology and disease. Orchestrating these gene expression programs are regulatory factors that recognize sequence elements distributed across the genome and transcriptome. The transcriptomic differences between two biological states are typically large, with hundreds or thousands of genes differing in splicing or expression, but these differences typically result from variation at a much smaller set of regulatory factors. Disentangling gene expression programs in terms of regulatory factors and their targets is critical to understanding the genomic basis of physiology and doing so requires determining the rules by which these factors exert their activities.

Alternative splicing greatly expands proteomic diversity by allowing a single gene to generate dozens of distinct splicing isoforms (spliceoforms), often with functions tuned to specific tissues or developmental stages. Splicing can also impact gene expression levels as some spliceoforms may be subject to turnover through pathways such as nonsense-mediated mRNA decay. Approximately 10–20% of disease-causing mutations are estimated to result in changes to splicing levels of individual exons^1,2^, while perturbations of splicing factor (SF) genes can broadly disrupt splicing. Several SFs are known oncogenes or tumor suppressors^3,4^ and the resulting disruptions to splicing programs represent vulnerabilities that can be exploited^5^. In a therapeutic context, splicing defects at individual exons can often be corrected by splice-switching antisense oligonucleotides (ssASOs)^6,7^. However, brute-force tiling is often required to identify effective ASOs because the relevant SFs and splicing regulatory elements (SREs) are unknown. Interpretation of clinical variants that may impact splicing is also made challenging by our incomplete knowledge of SF activities and SREs^8^. Elucidating precise rules governing SF activity would facilitate variant interpretation, ASO design and the identification of SFs that contribute to disease.

A number of exploratory approaches have been developed to assess how the presence of particular motifs or *k*-mers are associated with variation in transcription^9,10^, mRNA stability^11^ or splicing^12,13^. Because these models require discovering the putative activity of hundreds or thousands of motifs and *k*-mers every time they are applied, they do not incorporate prior knowledge of each factor’s regulatory activity, extract generalizable insights or even directly identify the causal factors. More focused approaches have leveraged perturbation experiments to predict the targets of specific SFs by incorporating expert knowledge of the factor and using crosslinking and immunoprecipitation (CLIP) data to inform binding sites^14–16^. However, the challenge of crafting these models to the specific details of the SF’s binding and activity has limited their application to a few well-studied factors and a reliance on crosslinking data still ties the insights to exons where the SF was detectably bound in specific experimental data. This need to tailor models to the specifics of particular SFs has been sidestepped by recent applications of deep learning to the prediction of splicing from the nucleotide sequence^2^. However, the learned models are black boxes containing myriad parameters representing abstract transformations rather than biological statements. Follow-up in silico experiments are typically required to predict *cis*-regulatory elements or the associated positional activities^17^.

**Fig. 1:**
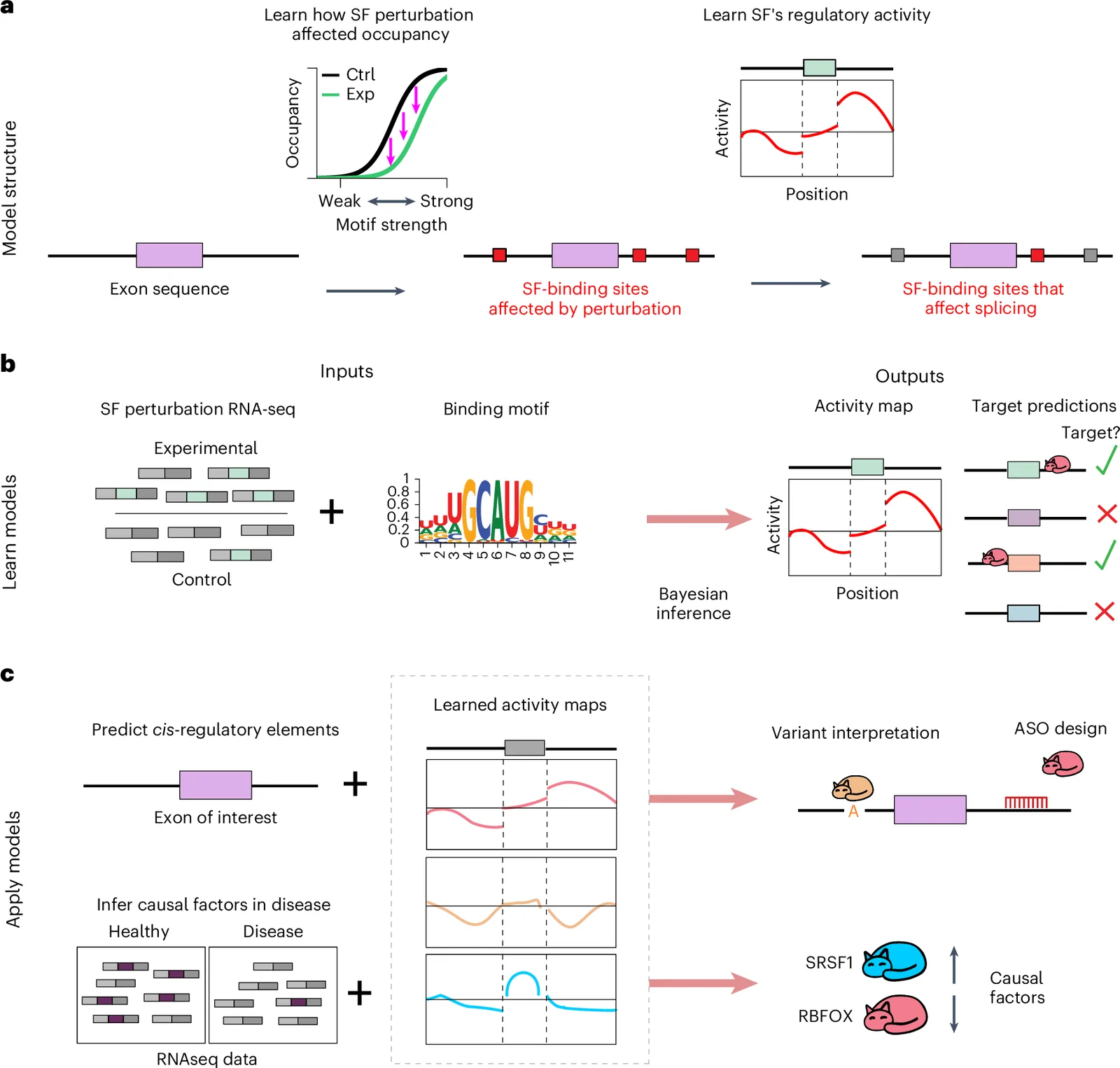
High-level overview of the KATMAP model and its applications. **a**, The general workflow of how KATMAP predicts the regulation of individual exons on the basis of their sequence. **b**, Learning the rules by which an SF regulates splicing requires only an RNA-seq dataset where that SF was perturbed and some model of its sequence specificity. Bayesian inference determines which splicing models are consistent with the observed splicing changes, yielding an activity map describing the SF’s position-specific regulatory activity and identifying which exons are the SF’s direct targets and which splicing changes represent indirect effects. **c**, Examples of how learned models can be applied to interpret splicing in terms of SF regulation. Top, scoring an exon of interest to predict the SREs and SFs that control its splicing, with application to variant interpretation and ASO design. Bottom, identifying the causal SFs underlying splicing differences observed in a given RNA-seq dataset.

Here, we present a flexible and interpretable model for learning regulatory activity from knockdown or overexpression RNA-seq data, which can then be applied to generate predictions and interpret sequencing data. Our approach, knockdown activity and target models from additive regression predictions (KATMAP), explicitly models the effect of knockdown by assigning changes in SF occupancy at potential binding sites throughout the transcriptome to predict regulation at individual exons (Fig. 1a). We use a hierarchical framework that tailors modeling assumptions to each SF and learns the nonlinear biophysical relationship between motif affinity and binding, allowing KATMAP to be broadly applied off-the-shelf to diverse SF datasets. Learning a regulatory model requires as inputs only the results of a perturbation experiment and some knowledge of the factor’s binding motif, such as a position-weight matrix (PWM) (Fig. 1b). The learned model describes the position-dependent regulatory activity for the SF of interest. Once learned, these models can be applied to any exon of interest to identify *cis*-elements and associated SFs regulating its inclusion, which can simplify ASO design, or to any RNA sequencing (RNA-seq) dataset to infer which SFs explain the splicing differences observed between conditions (for example, to identify disease drivers; Fig. 1c).

## Results

### Overview of the KATMAP model

KATMAP provides a framework for learning interpretable models of splicing regulation from perturbation experiments, which can be applied to interpret splicing regulation in other datasets. To learn a regulatory model from a perturbation experiment, KATMAP uses a model of the SF’s binding motif to explain the splicing changes uncovered in a differential splicing analysis of the perturbation experiment (Fig. 1b). The differential splicing calls can be defined with the user’s software of choice, although we use rMATS results^18^ in our analyses (instructions for organizing the results of MAJIQ v2 (ref. ^19^) are also provided in the documentation). Sequence specificity can be represented with a PWM or position-specific affinity matrix (PSAM), a collection of weighted PWMs and PSAMs or a ProBound model^20^. The PWMs may be derived from in vitro binding data, eCLIP (for example, with MCROSS^21^) and, for full interpretability, should represent the binding specificity of the SF of interest or a close homolog. Motifs discovered de novo from the RNA-seq data (for example, with MEME-suite) can also be used as input. Of course, motif discovery analyses may identify motifs reflecting secondarily perturbed SFs; hence, the models learned using de novo motif discovery will not be as interpretable as when the motif is known.

KATMAP infers which motifs were affected by the perturbation experiment and distills the observed splicing changes into a description of the SF’s position-specific regulatory activity, which we term an activity map (Fig. 1a,b). This learned model provides a scoring system for predicting whether an exon is regulated by an SF on the basis of (1) whether there are binding motifs of appropriate strength and (2) whether these motifs are located in exon-proximal regions where the SF exerts regulation. These criteria allow KATMAP to predict which splicing changes reflect the direct targets of the perturbed SF and which are the indirect effects of perturbation (Fig. 1b). Focusing on an exon of interest, our models can be used to predict which SFs regulate that exon and identify the corresponding *cis*-elements that are bound (Fig. 1c). Alternatively, these models can be applied to infer which SF perturbations underlie the splicing profile of an RNA-seq dataset relative to a control by asking whether the exons with altered splicing overlap with the targets of particular SFs. The KATMAP python library (with pretrained models) is available from GitLab (https://gitlab.com/LaptopBiologist/katmap) and provides a unified framework for these applications.

### Architecture of the regression model

The intuition guiding our model is as follows. Before a perturbation to the activity of a sequence-specific SF, sequence motifs in the transcriptome are bound by the SF at varying levels of occupancy. We assume the factor can only influence the splicing reaction if its binding sites are properly situated relative to the exon and that binding elsewhere has negligible effect. After knockdown, the free protein concentration decreases, leading to reduced occupancy across the protein’s binding sites. If binding is lost from a regulatory region, then loss of regulation results in a change in exon inclusion. The greater the decrease in binding, the more likely it is to affect inclusion. The logic is similar for SF overexpression, except that free protein concentration, occupancy and regulation increase upon perturbation.

Following this logic, KATMAP models splicing changes in terms of changes in SF binding to the transcript. As observations, we use the results of a differential splicing analysis that has labeled exons as upregulated, downregulated or not significantly changed. We score the sequence around each of these exons using a model of the factor’s sequence specificity. KATMAP aims to predict whether and in which direction each exon changes in splicing by answering two questions: (1) Are there binding sites near the exon that were affected by the SF knockdown? (2) Where must the SF bind near an exon to regulate its inclusion? We frame this as a generalized additive model that represents SF binding and regulation with nonlinear terms but accounts for other features predictive of detectable splicing change (such as the splicing event’s read count) through additional linear terms.

**Extended Data Fig. 1:**
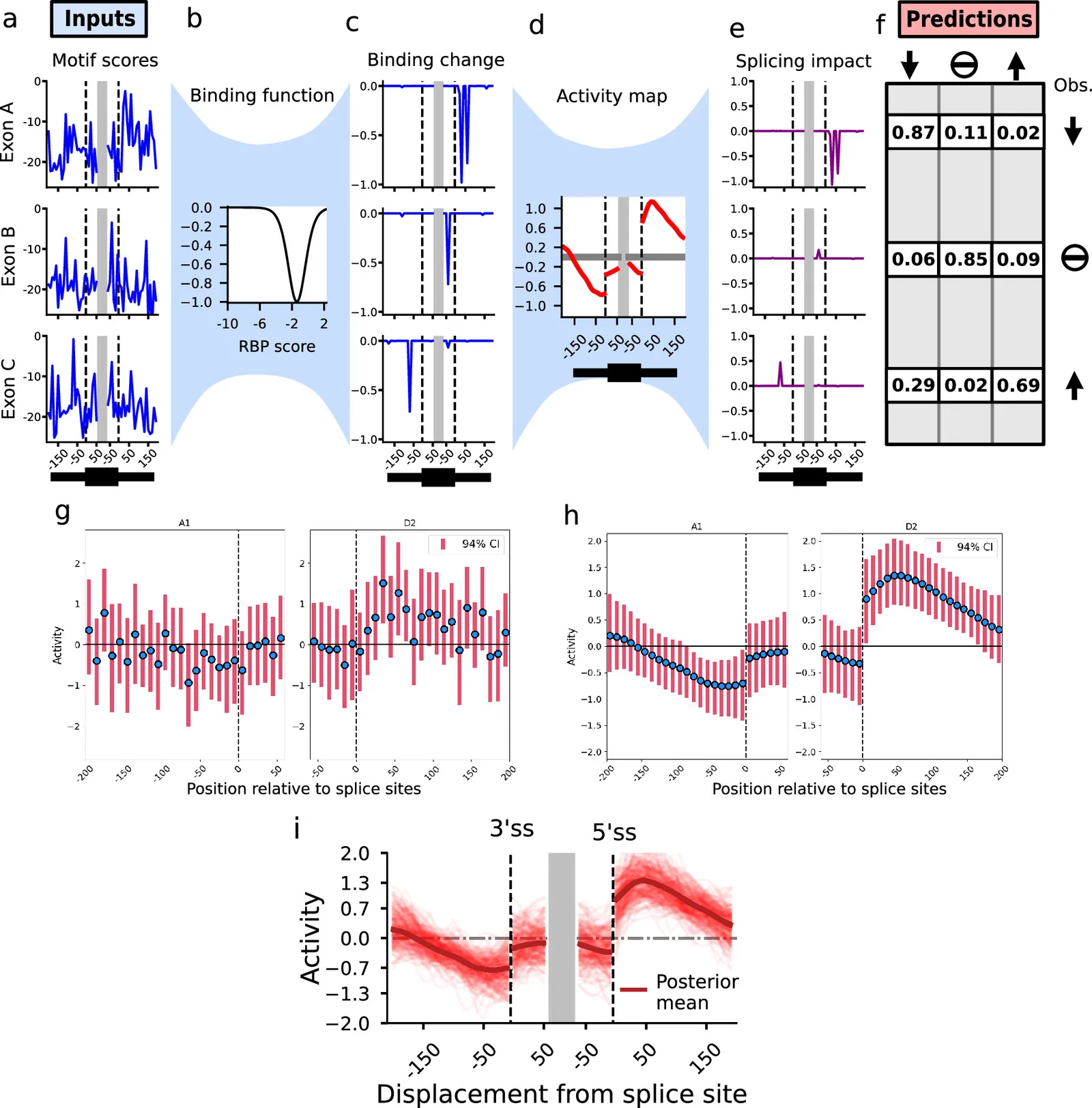
Detailed overview of model architecture. Examples of the KATMAP model applied to three real exons in the RBFOX2 (HepG2) knockdown. **a**) RBFOX2 motif scores assigned to sequences around the 3 and 5SS. **b**) The binding function that transforms the scores into relative changes in binding. **c**) Predicted changes in binding obtained by passing the motifs scores (**a**) through the binding function (**b**). **d**) An activity map describing the effect on splicing of RBFOX2 binding at positions near the 3 and 5SS. Discontinuities at the splice sites allow activity to differ between introns and exons. **e**) The predicted splicing impacts, computed by multiplying the binding changes (**c**) by the activity map (**d**). **f**) The predicted probabilities of being downregulated (down arrow), unchanged (dash), or upregulated (up arrow) for these exons and the observed splicing changes. **g**, **h**) The activity maps with 94% credible intervals inferred from an RBFOX2 knockdown in HepG2 cells with priors assuming independence among the activity coefficients (**g**) or with our full model’s assumption of spatial correlation (**h**). **i**) A posterior sample of 200 activity maps for the RBFOX2 (HepG2) KD. Source data

Our model begins by scoring all potential binding motifs around an exon’s splice sites (SSs), extending 200 nt on the intronic and 60 nt on the exonic sides, using a model of the SF’s motif (Extended Data Fig. 1a). These *motif scores* are then transformed to changes in binding using a binding function based on a biophysical description of how occupancy changes after a decrease in SF availability (Extended Data Fig.1b,c). The shape of the binding function is controlled by a ‘most relevant affinity’ parameter that defines which binding sites were most affected by the knockdown (that is, shifts the function along the *x* axis) and an ‘affinity scale’ parameter that defines the range of motif scores affected by the knockdown (that is, widens or narrows the binding function); we refer to these collectively as the binding parameters (Methods).

We represent the SF’s position-specific regulatory activity with a set of parameters termed the activity map (Extended Data Fig. 1d), which assigns enhancing (>0) or suppressing (<0) regulatory activity to different positions on the intronic and exonic sides of 3′ and 5′ SSs (3SS and 5SS). These regulatory activities are shared across all exons in the analysis. Critically, we assume that activity varies smoothly with position, with discontinuities at the exon–intron boundaries that allow exonic and intronic binding to have distinct activities. The degree of smoothness is learned from the data, rather than assumed a priori, in the form of two spatial correlation parameters. We multiply each change in binding by the corresponding activity to determine its impact on the splicing outcome (Extended Data Fig. 1e). The sum of these products defines the SF’s splicing impact on the exon, making the simplifying assumption that regulation is additive.

Lastly, we incorporate these splicing impacts into a regression framework that assigns each exon a probability of being upregulated, downregulated or unchanged (Extended Data Fig. 1f). In addition to SF binding, the unperturbed level of exon inclusion and gene expression impact our statistical power to detect splicing changes. Specifically, exons with inclusion levels (percent splicing inclusion (PSI) values) near the boundaries of 0% or 100% inclusion are less likely to be detected as downregulated or upregulated, respectively, while statistical power to detect splicing changes is limited for exons in genes with low expression. We include these confounding variables (for example, unperturbed inclusion level and expression) and their squares as linear predictors, whose effects are controlled for by a set of linear coefficients.

This model architecture answers the two questions we posed in an integrated fashion. SF binding and regulatory activity of the perturbed SF are learned simultaneously, without the need to define arbitrary cutoffs for binding or activity. The activity map provides a simple description of the SF’s position-specific activity, identifying where a binding site must occur to influence an exon’s inclusion positively or negatively, which we propose as an alternative to the traditional RNA map.

The goal of KATMAP’s inference algorithm is to determine which parameter values provide good explanations for the observed splicing changes. Prior target prediction models obtained point estimates of the most likely parameter values^14^, but we wished to also quantify our uncertainty by identifying the range of plausible hypotheses. We, therefore, used Bayesian inference to precisely evaluate the plausibility of hypotheses given observed data (Extended Data Fig. 1i), which we accomplish by coupling adaptive importance sampling^22^ with integrated nested Laplace approximations (INLA)^23^. This allows us to compute point estimates of the most plausible parameters, quantify our uncertainty with credible intervals (CIs) and propagate this uncertainty forward into our predictions of regulation. Finally, the learned parameters can be used to score new sequences.

### KATMAP accurately infers parameters and quantifies uncertainty

**Extended Data Fig. 2:**
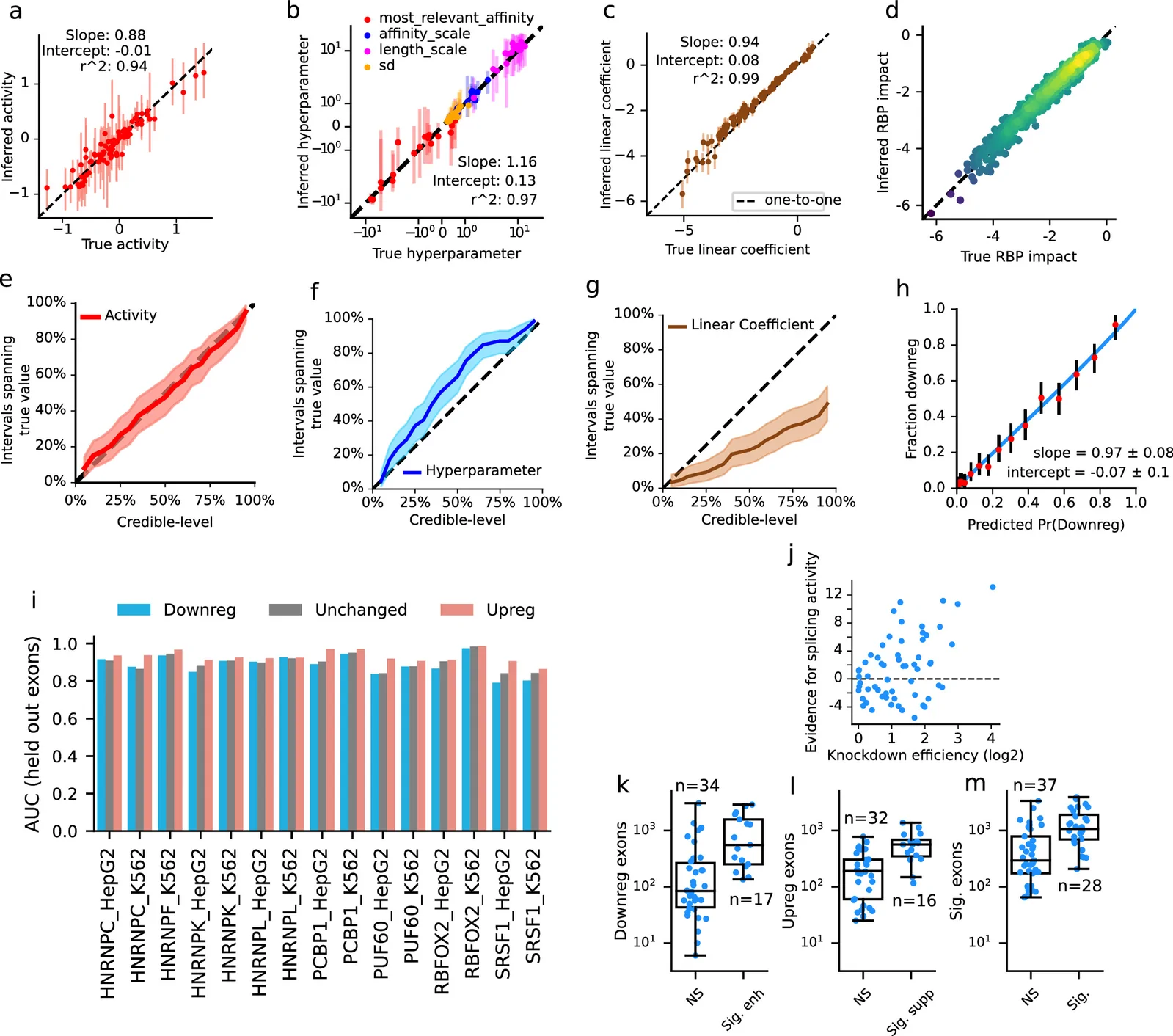
Assessment of KATMAP’s performance. **a**-**c**) True activities (**a**), hyperparameters (**b**), and linear coefficients (**c**) used in the 21 simulated datasets versus the values (with 94%-CI) recovered by inference. The dotted line represents the one-to-one relationship expected for completely unbiased estimates. Only four activities per simulation are shown to ensure independence of the plotted values. **d**) An example comparing splicing impacts used to simulate splicing changes and the splicing impacts recovered by inference. **e**-**g**) Coverage plots of how frequently credible intervals contain the true activity (**e**), hyperparameter (**f**), or linear coefficient (**g**), for different CI-levels. The dotted line represents the expectation that frequency equals the credible level. **h**) Calibration plot for the PUF60 (K562) knockdown, comparing average Pr(Downreg) and fraction truly downregulated for sets of 200 held out exons. The blue line is a binomial regression fit, the dots are posterior means with 94%-credible intervals. **i**) Area-under-curve (AUC) statistics summarizing how well learned models classify held-out exons as unchanged, up- or downregulated. **j**) The relationship between the efficiency of an ENCODE knockdown as shrinkage estimate of the gene’s of log2 fold-change in the DEseq analysis. **k**-**m**) The numbers of differentially spliced exons in knockdowns where KATMAP identified enhancing (**k**) or suppressing (**l**) activity, or activity in either direction (**m**). Boxes are upper and lower quartiles, and whiskers extend to the furthest data points from the box within 1.5 of the interquartile range (IQR). Source data

Before applying our model to interpret experimental data, we wanted to confirm that our inference strategy could correctly infer regulatory activity. We simulated 21 SF knockdown datasets with known parameter values and found that KATMAP robustly recovers the true parameters (Extended Data Fig. 2a–c). Importantly, our posterior samples properly quantify the uncertainty regarding the activity and binding parameters (Extended Data Fig. 2e,f). Because the activity and binding parameters are accurately recovered, so are the splicing impacts used to simulate the splicing changes (Extended Data Fig. 2d). The linear coefficients are slightly underestimated (Extended Data Fig. 2c), meaning that their true values are less frequently captured by the CIs than expected (Extended Data Fig. 2g), likely because of the multivariate Gaussian approximation to their conditional posterior^23^. Nonetheless, the accurate recovery of the activity and splicing impacts shown above indicates that this subtle bias does not meaningfully interfere with the primary function of the linear coefficients in controlling for potential confounders.

To assess performance on real data and ensure that the models had not overfit, we selected 15 knockdowns with many splicing changes. For each experiment, we trained on 70% of the differentially spliced exons, holding out the remaining 30% as part of the test set. We evaluated our models against these held-out exons, comparing the true splicing changes to our predictions of which exons were upregulated, downregulated or unchanged upon knockdown. We found that the predicted probabilities were well calibrated (Extended Data Fig. 2h) and effective at predicting the splicing changes in the held-out exons (Extended Data Fig. 2i).

### KATMAP robustly infers splicing regulatory activity

**Fig. 2:**
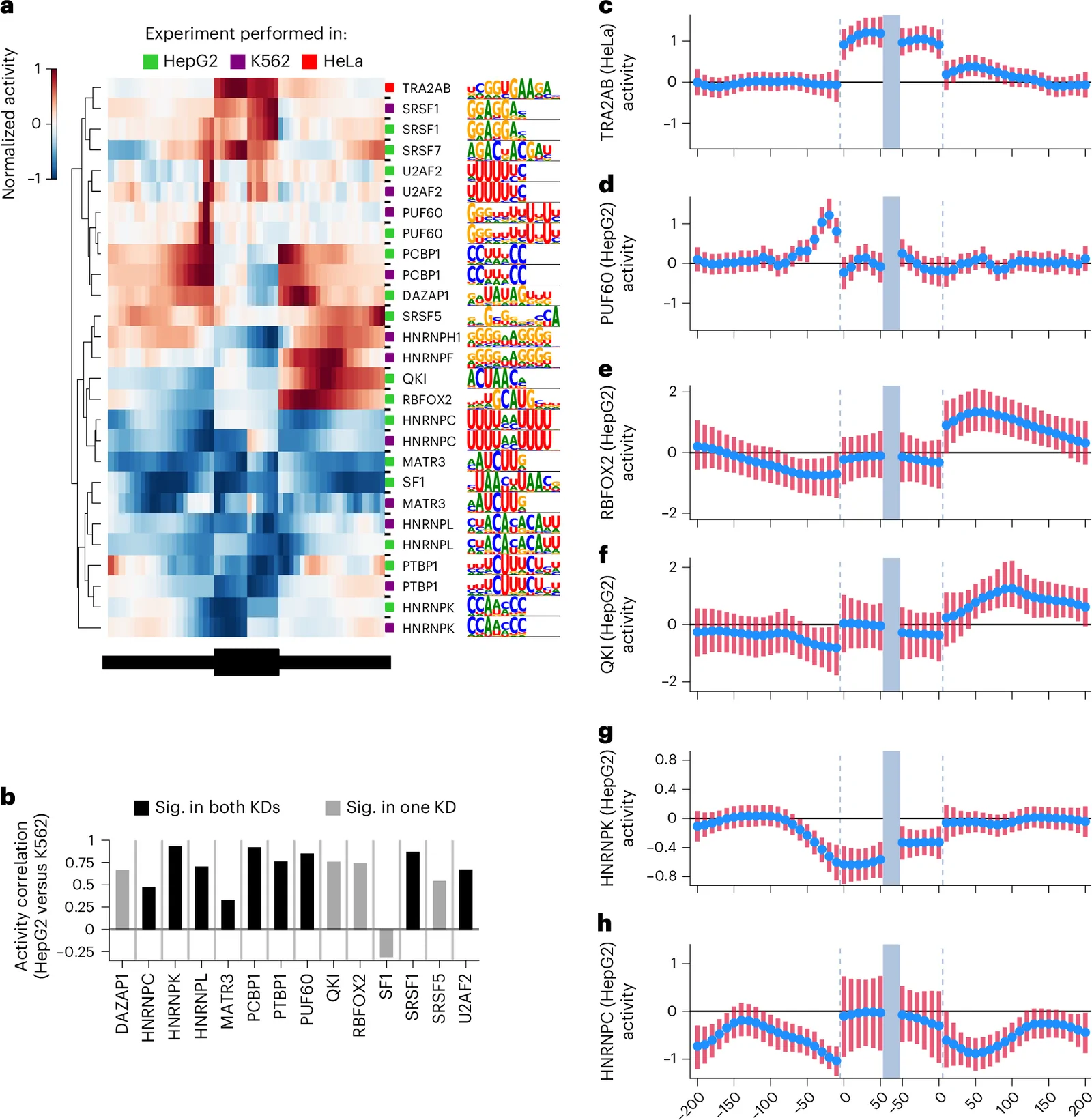
KATMAP robustly infers splicing activity. **a**, Significant activity maps and their motifs organized by hierarchical clustering. For ease of comparison, activity is normalized relative to the maximum absolute activity in each map. If the binding model included multiple motifs, only the top motif is shown. **b**, The correlation between activity maps for the same RBP inferred from different knockdowns. KD, knockdown. **c**–**h**, Activity maps for TRA2A/B (**c**), PUF60 (**d**), RBFOX2 (**e**), QKI (**f**), HNRNPK (**g**) and HNRNPC (**h**). The blue dots indicate the posterior mean and the lines are 94% CIs (*n* = 1,682, 1,420, 1,024, 1,106, 1,047 and 1,122 posterior samples, respectively). Source data

KATMAP aims to distill perturbation experiments into practical descriptions of the perturbed SF’s splicing regulatory activity. Here, we applied KATMAP to knockdown data from the ENCODE project for 35 SFs for which we also had good models of the protein’s sequence specificity (Supplementary Table 1)^24^. We used RNA-bind-n-seq-derived PSAMs as the binding model for 23 SFs^25,26^ and RNAcompete-derived PWMs for the remaining 12 (refs. ^27,28^). A total of 18 SFs showed significant enhancing or repressing activity maps and outperformed a reduced model (with splicing activity excluded), with nine displaying enhancing activity, six showing repressing activity and three with both enhancing and repressing activity (Fig. 2a). Among these 18, 14 had available knockdown/RNA-seq data from both lymphocyte-derived K562 cells and hepatocyte-derived HepG2 cells. For all but one factor (SF1), the activity maps were consistent between the two knockdowns, highlighting the reproducibility of our inferences (Fig. 2b).

The evidence for splicing activity correlated with knockdown efficiency (*r* = 0.45; Extended Data Fig. 2j) and enhancing or repressing activity was only confidently detected in knockdowns with at least 100 downregulated or upregulated exons, respectively (Extended Data Fig. 2k–m). For example, both ENCODE knockdowns for the splicing activator TRA2A yielded <100 downregulated exons and no clear evidence of activity, perhaps because of compensation by paralog *TRA2B*. However, applying our model to a *TRA2A/B* double-knockdown experiment with >1,000 downregulated exons^29^ recovered strong evidence of the expected exonic enhancing activity (Fig. 2c).

Examining the significant activity maps identifies three distinct groups of splicing activators. First, we identify SRSF1, SRSF7 and TRA2A/B as exhibiting the classic exonic enhancing pattern of SR protein regulation^30^ (Fig. 2a,c). TRA2A/B shows similar activity near both SSs, while SRSF1 is inferred to have stronger activity near the 5SS and SRSF7 near the 3SS. Second, several polypyrimidine-binding factors show enhancing activity directly upstream of the 3SS, consistent with polypyrimidine tract (PPT) definition. For U2AF2 (which canonically defines the PPT^31^) and its homolog PUF60 (ref. ^32^), we infer intronic enhancing activity just upstream of the 3SS (Fig. 2a,d). U2AF2’s inferred activity is constrained to within 20 nt of the 3SS, while PUF60’s activity is slightly more upstream and extends 40–60 nt into the intron, consistent with observations of extended PPTs at PUF60-responsive exons^33^. Similar upstream enhancing activity is inferred for PCBP1 (Fig. 2a) but with additional activity downstream of the 5SS, consistent with prior observations of C-rich motifs upstream and downstream of exons activated by PCBP1 and PCBP2 (ref. ^34^). KATMAP failed to detect the expected enhancing activity of the branch point-binding factor SF1 (ref. ^35^) and the exonic enhancing activity of SRSF5, perhaps because of indirect effects of other SFs obscuring the signal in these knockdowns.

While upstream activators display greatest activity near the 3SS, a third group of factors with downstream enhancing activity (DAZAP1, HNRNPF/H, QKI and RBFOX) have their greatest activity tens of nucleotides away from the 5SS and influence splicing over a wider intronic window (Fig. 2a). Among these, two pairs of factors have strikingly similar activities. The related poly(G)-binding factors HNRNPF and HNRNPH1 both enhance inclusion when bound in the downstream intron while suppressing inclusion in the exon. HNRNPH1 was one of the first SFs demonstrated to activate and repress inclusion in a position-dependent manner^36,37^. RBFOX2 and QKI also share patterns of activity (Fig. 2e,f), enhancing downstream while suppressing upstream; however, unlike HNRNPF/H, they are not related and bind distinct motifs (YGCAUG versus ACUAAC, respectively).

In contrast to splicing activators, all inferred repressors displayed activity in multiple regions (Fig. 2a). The PCBP-related HNRNPK protein shows silencing activity upstream of the 3SS and in the exon (Fig. 2g), consistent with its role as a splicing repressor^38^ and with observations that PCBP2 and HNRNPK can regulate the same exon in opposing directions^39^. We find a similar pattern of upstream and exonic silencing activity for PTBP1, as well as evidence of downstream enhancing activity (Fig. 2a), all of which is consistent with prior studies^40^. This exonic activity of HNRNPK and PTBP1 stands in contrast to the pyrimidine-binding upstream activators, which lacked exonic activity. The other repressors have even wider regions of suppression. HNRNPC’s silencing activity is strongest in the ~100 nt upstream and downstream of the exon but has two additional regions of silencing activity at ±200 nt (Fig. 2h). HNRNPC is known to form tetramers, with repression suggested to result from looping out the exon, which may explain the multiple regions of activity^41^. HNRNPL activity maps also display repressing activity both upstream and downstream (Fig. 2a) but also indicate repression within the exon, consistent with prior work^42,43^. MATR3 (Matrin3) has even broader regions of repression, extending throughout both upstream and downstream introns in the HepG2 model but only showing evidence of upstream repression in K562 cells (Fig. 2a). This broad silencing activity is consistent with CLIP-based enrichment maps of MATR3-repressed exons, which found MATR3 uniformly distributed hundreds of bases into the flanking introns^44^.

**Extended Data Fig. 3:**
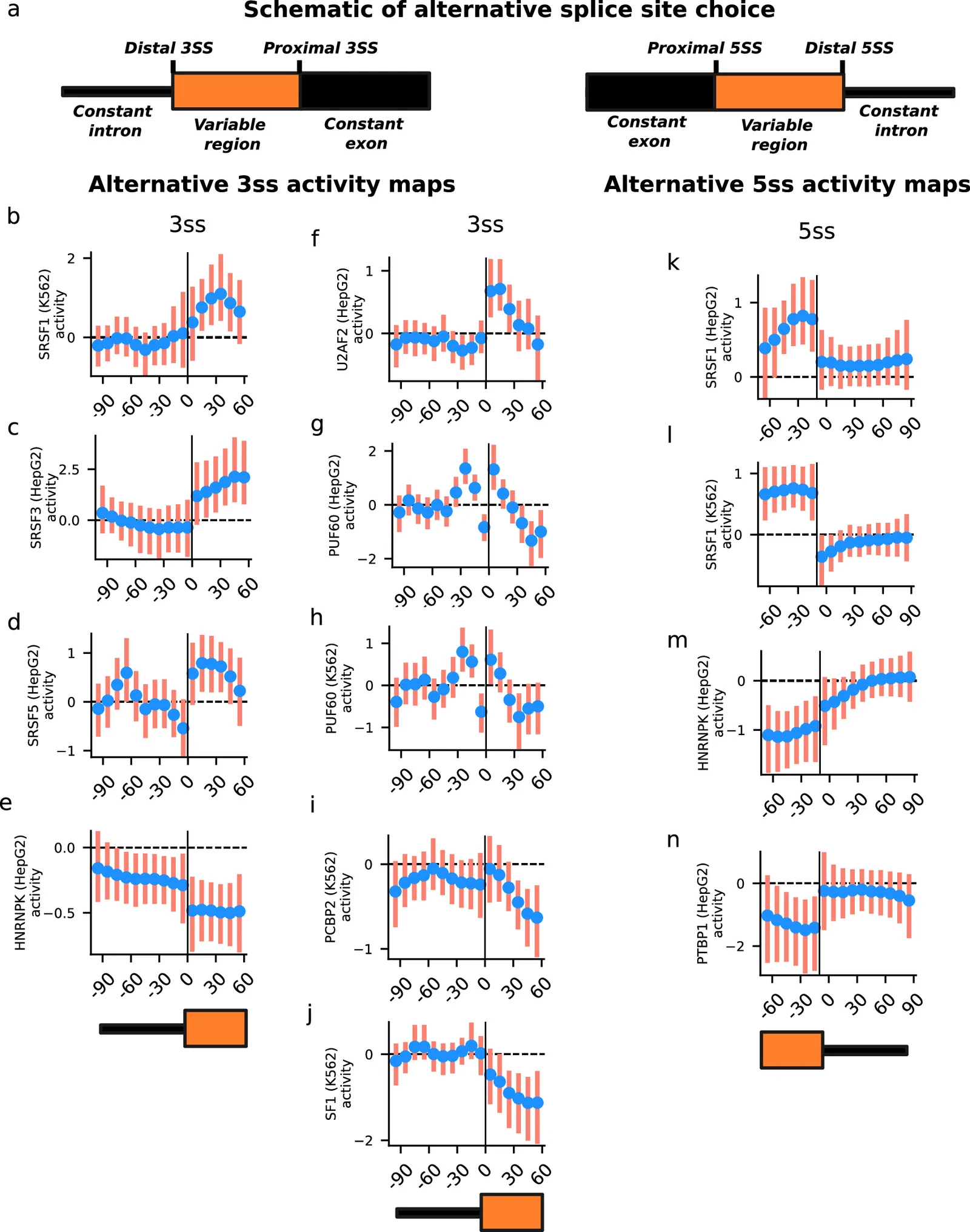
Activity maps learned from alternative 3 and 5SS splicing events. **a**) Schematics of alternative 3 and 5SS events, labelling the splice sites proximal and distal to the fixed exonic segment. **b**-**i**) All significant activity maps (with 94%-CI) learned from alternative 3SS events with significant enhancing or repressing activity. **k**-**n**) Activity maps (with 94%-CI) learned from alternative 5SS events with significant enhancing or repressing activity. N = 1100 (**b**), 1208 (**c**), 1233 (**d**), 1264 (**e**), 1150 (**f**), 973 (**g**), 962 (**h**), 1027 (**i**), 1097 (**j**), 1227 (**k**), 1094 (**l**), 1205 (**m**), 950 (**n**) posterior samples. Source data

While we focus on cassette exons, KATMAP’s statistical framework is general enough to accommodate other classes of splicing events. To explore this, we modeled regulation of alternative 3SS and 5SS choice, predicting usage of the SS distal to the constant exonic region on the basis of SF-binding sites in the constant intronic region and the variable exon segment between the two alternative SSs (Extended Data Fig. 3a). The evidence for splicing regulation inferred from alternative SSs was typically weaker than observed for cassette exons, perhaps because the inferences were often based on fewer significant events and shorter sequence regions. While fewer models satisfied our model comparison criterion, the activity maps showing significant enhancing or repressing activity were typically consistent with those learned from cassette exons (Extended Data Fig. 3b–n). This demonstrates that KATMAP can model alternative SS choice and suggests that our model comparison criterion may be conservative. The interpretation of activity in the variable region differs from the cassette exon models, with positive values favoring inclusion of the variable region as an exon (either by enhancing the distal SS or repressing the proximal SS) and negative values favoring usage of the variable region as an intron. For example, binding of PCBP2 and SF1 in the variable region between alternative 3SSs favors its usage as an intron (Extended Data Fig. 3i,j), likely reflecting activation of the proximal 3SS. This is consistent with the intronic enhancing activity observed for PCBP1 in the cassette exons maps (Fig. 2a) and SF1’s known role in branch point definition^35^.

The diverse position-dependent patterns of splicing regulation we uncover underscore the power and flexibility of KATMAP’s approach to describing activity. Our assumption that this activity changes smoothly with position (except at exon–intron boundaries) is biologically plausible^45^ and learning this smoothness from the data (rather than assuming this a priori) improved statistical power by pooling information spatially (Extended Data Fig. 1g,h) and allowed KATMAP to detect both broad and narrow ranges of activity. We were able to robustly and reproducibly uncover this activity by modeling the sequences of exons and introns without using crosslinking data, avoiding the bias toward exonic signals of eCLIP-based RNA maps^24^.

### Activity maps define regulatory targets

**Fig. 3:**
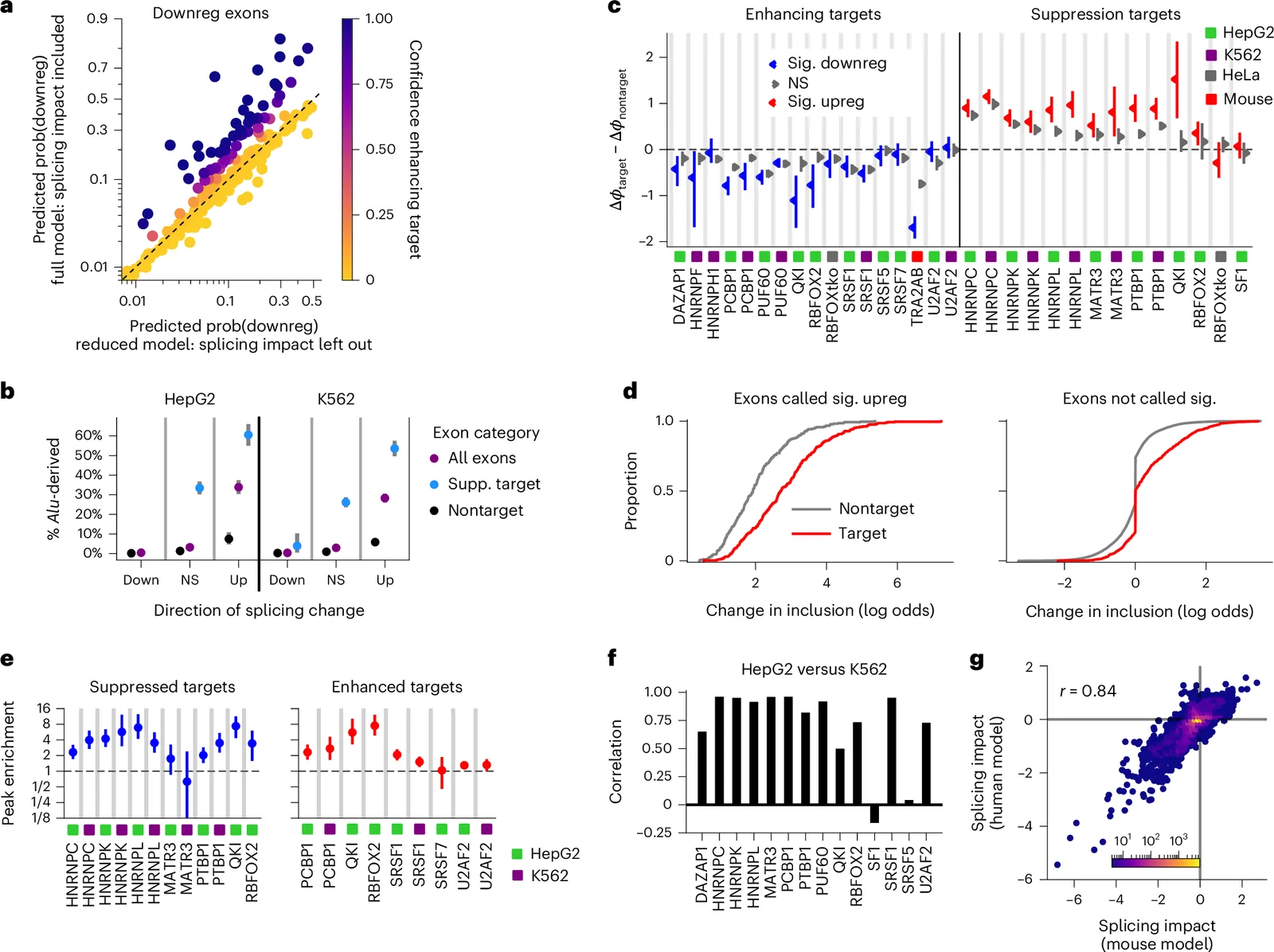
KATMAP predicts regulatory targets. **a**, The probabilities of downregulation predicted by the full model and a reduced model lacking splicing impacts for exons downregulated upon RBFOX2 knockdown (HepG2). Exons where the full model assigns a higher probability than the reduced model are likely enhancing targets, while those assigned the same predictions by both models are likely nontargets. The axes are in log odds scale and depicted in the range 0.01–0.99. **b**, The fraction of exons derived from *Alu* among targets of HNRNPC’s suppression, nontargets or all exons that changed in a given direction, computed for each of the three splicing categories, with the posterior means and 94% CIs. **c**, The difference in average inclusion level change between confident targets and confident nontargets among exons that were significantly altered by knockdown (red and blue) and those that were not (gray). NS, not significant. The 94% CIs were obtained by Bayesian bootstrapping (*n* = 2,000). **d**, eCDF plots of the distributions of inclusion level changes at targets and nontargets upon HNRNPC knockdown (HepG2) for exons significantly upregulated upon knockdown (left) and nonsignificant exons (right). It is the difference in the means of the target and nontarget distributions that is shown in **c**, where we excluded exons where the data supported a confidently small effect of knockdown (spike at 0, ∣*Δψ*∣ < 10^−3^) to focus on those exons called nonsignificant potentially because of a lack of statistical power; the estimates with all exons included are shown in Extended Data Fig. 4b. **e**, The enrichment of eCLIP peaks in differentially spliced targets compared to nontargets differentially spliced in the same direction, with 94% CIs computed under a beta-binomial model (*n* = 2,000 posterior samples). The enrichment summarizes eCLIP peaks that span the significant regions of a given SF’s activity map. **f**) The correlation in splicing impacts of exons across the human transcriptome computed from the HepG2 and K562 models for SFs with data for both cell types. **g**, Comparing RBFOX splicing impacts in the mouse transcriptome computed using the human RBFOX2 (HepG2) model and a model inferred from mouse *Rbfox1/2/3* triple-knockout data. Source data

The changes observed in a differential splicing analysis after knockdown are a mixture of direct SF targets and the indirect effects of the perturbation. When studying an SF of interest, these indirect effects can obscure the underlying biological signal. However, the activity maps inferred by KATMAP lead to a natural definition of an SF’s direct targets as exons with motifs of appropriate strength and location for regulation, distinguishing these from exons that lack binding motifs in regions expected by the model to impact inclusion (predicted nontargets). This is summarized in the ‘splicing impact’ score assigned to each exon, computed by multiplying predicted changes in binding by the SF’s activity map (Extended Data Fig. 1c–e and Supplementary Table 2). We consider an exon to be a direct repression or enhancement target if the splicing impact of the exon is confidently nonzero and large enough to improve predictions over a reduced model that excludes all splicing activity (Fig. 3a). Exons not predicted to be targets reflect several possibilities. Some predicted nontargets should reflect exons truly not bound or regulated by the SF (that is, true nontargets), while others might reflect true direct targets where the nature of binding or regulation is more complex than that assumed by the model or where the knockdown/RNA-seq data did not provide sufficient power for confident identification. To investigate these possibilities, we compared our target predictions to the effect sizes of knockdown on inclusion, crosslinking data and prior literature.

Comparing these target predictions to the literature recovers previously described targets. For example, PTBP1 is known to silence exons in its paralogs *PTBP2* (ref. ^46^) and *PTBP3* (ref. ^47^) during development, as well as in *RTN4* (ref. ^48^) and *FLNA*^49^, all of which were identified as direct targets of PTBP1 by KATMAP. To assess our target predictions more comprehensively, we considered HNRNPC, which is known to silence exons derived from the highly abundant transposable element *Alu*^50^. These *Alu*-derived exons provide a ground truth for validating our target predictions. Intersecting predicted HRNPNC targets with the UCSC RepeatMasker track (https://www.repeatmasker.org/) confirmed that roughly 30% of all exons upregulated upon HNRNPC knockdown are *Alu*-derived. When we divide these into predicted targets and nontargets, ~55% of predicted targets are derived from *Alu* compared to only ~5% of nontargets. Thus, KATMAP’s target predictions recover HNRNPC’s known silencing of *Alu* exons, strongly sorting *Alu*-derived exons into the set of targets, with few labeled as nontargets (Fig. 3b), supporting the model’s classifications of direct versus indirect targets.

**Extended Data Fig. 4:**
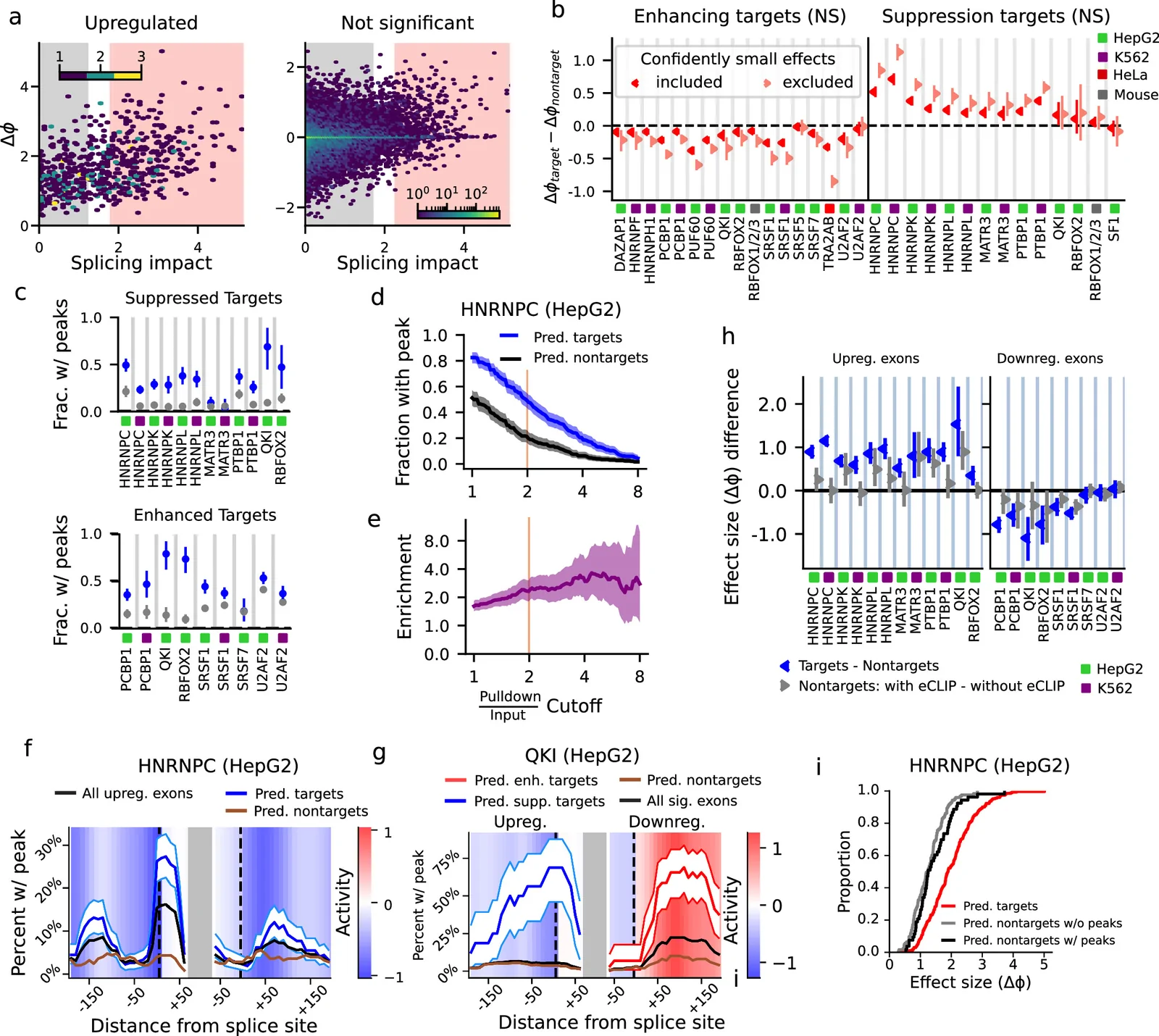
Additional validation of target predictions. **a**) The relationship between the change in the log2-odds of inclusion, *Δϕ*, and splicing impact for exons called significantly upregulated (left) and those not called significantly affected (right) by HNRNPC knockdown in HepG2. Red and grey shaded regions indicate confident targets and nontargets respectively. **b**) The difference in average effect size (with 94%-CI, n=2,000 posterior samples) between confident targets and confident nontargets among exons called nonsignificant with confidently small effect sizes (∣*Δψ*∣ < 10^−3^) included in (red) or excluded from the averages (pink). **c**) The fraction of knockdown upregulated predicted HNRNPC targets (blue) and nontargets (grey) with pulldown/input eCLIP enrichment exceeding the specified cutoffs (with 94%-CI computed under a beta- binomial model). **d**) The enrichment (target/nontarget) of eCLIP peaks at the specified cutoffs (with 94%-CI computed under a beta-binomial model). **e**) The fraction of differentially spliced predicted targets and nontargets with eCLIP peaks at different fold-enrichment cutoffs (with 94%-CI, n=2,000 posterior samples). Used to compute the peak enrichments in Fig. 3e. **f**) The percent of upregulated exons in the HNRNPC (HepG2) knockdown with eCLIP peaks (pulldown/input≥2) plotted for all upregulated exons (black), predicted suppression targets (blue, with 94%-CI), and predicted nontargets (brown). The regulatory activity associated with each position is indicated by the background color. **g**) eCLIP peaks at diffentially spliced exons in the QKI (HepG2) knockdown. As in **f**, but summarizing the signal around the 3SS for upregulated exons (left) and around the 5SS for downregulated exons (right). **h**) The average difference in effect size (with 94%-CI, n=2,000 posterior samples) between predicted targets versus nontargets (blue) compared to the difference at predicted nontargets with eCLIP peaks versus those without (grey). **i**) The distributions of effect sizes at significantly upregulated exons in the HNRNPC (HepG2) knockdown for predicted targets (red) versus predicted nontargets with (black) and without eCLIP peaks (grey). Source data

We then assessed the extent to which our target and nontarget predictions correspond to the sensitivity of exons to the SF’s knockdown. While our models were trained to predict the directions but not the magnitudes of changes in exon inclusion, among differentially spliced exons, those predicted to be direct targets had larger changes in inclusion following knockdown than predicted nontargets (Fig. 3c). For example, among exons upregulated upon HNRNPC knockdown, those with larger splicing impacts tended to have larger changes in inclusion (Fig. 3d and Extended Data Fig. 4a), with the average effect of knockdown being twofold higher at upregulated predicted HNRNPC targets compared to upregulated predicted nontargets. KATMAP’s predicted targets also include exons that were not called significant in the differential splicing analysis but have binding motifs properly situated to affect splicing. These may represent true targets where the RNA-seq coverage was inadequate to confidently detect a splicing change. Consistent with this, ‘nonsignificant’ exons predicted to be targets on average changed more in the expected direction than predicted nontargets (Fig. 3c,d and Extended Data Fig. 4a). KATMAP’s target predictions can, therefore, help interpret marginal signals in differential splicing analyses, using the presence of binding motifs to flag the subset most likely to be regulated by the SF.

KATMAP target predictions are based on sequence similarity to motifs identified in vitro and do not consider more complex binding modes, such as RNA structural preferences. To assess whether predicted targets were bound by the analyzed SF, we used crosslinking data. For the 11 SFs with credible activity maps that also had ENCODE eCLIP data, we asked whether among differentially spliced exons the predicted targets were enriched for eCLIP peaks compared to predicted nontargets. Of the 11 considered SFs, nine showed clear eCLIP enrichment at predicted targets, with most showing 2–8-fold higher frequency of peaks compared to predicted nontargets (Fig. 3e and Extended Data Fig. 4c). These enrichments are robust across different minimum $\frac{\rm{pulldown}}{\rm{input}}$ cutoffs for defining eCLIP peaks (Extended Data Fig. 4d,e). Depletion of eCLIP peaks from predicted nontargets suggests that these typically reflect exons not directly bound by the SF. The positional distribution of eCLIP peaks at targets was typically consistent with the factor’s activity map (Extended Data Fig. 4f,g). For example, in the QKI (HepG2) knockdown, ~70% and ~80% of differentially spliced suppression and enhancing targets had upstream and downstream QKI eCLIP peaks, compared to <5% and <15% of upregulated and downregulated predicted nontargets (Extended Data Fig. 4c,g), consistent with QKI’s upstream repressing and downstream enhancing activity (Fig. 2a). Furthermore, the peaks found at predicted nontargets typically displayed a more uniform positional distribution compared to predicted targets or to all exons differentially spliced in the same direction (Extended Data Fig. 4f,g), suggestive of nonregulatory binding. To assess this, we compared the effect sizes observed at predicted nontargets with and without eCLIP peaks. For most SFs, the predicted nontargets with eCLIP peaks were no more responsive to knockdown than those without and were always less responsive to knockdown than the predicted targets (Extended Data Fig. 4h,i). This pattern indicates that most nontargets with eCLIP peaks represent nonregulatory RNA-binding events rather than true regulatory targets missed by our model. Together, these observations suggest that KATMAP’s predicted targets are typically those directly bound and regulated by the knocked down SF, while the predicted nontargets more frequently reflect indirect effects of knockdown.

### Predicted targets generalize across biological contexts

**Extended Data Fig. 5:**
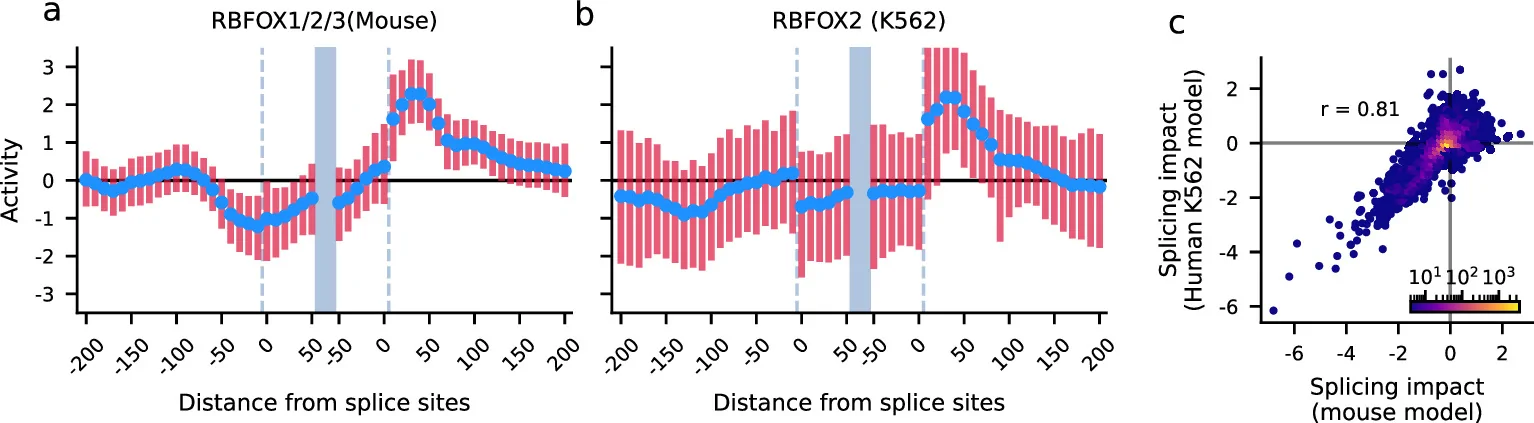
Comparing RBFOX models learned from mouse and human data. **a**) The RBFOX activity map (with 94%-CI, n=1,204 posterior samples) inferred from mouse *Rbfox*1/2/3 knockout data. **b**) The nonsignificant human RBFOX2 activity map (with 94%-CI, n=1,140 posterior samples) inferred from the K562 knockdown. **c**) Splicing impacts computed for the mouse exons using the nonsignificant human RBFOX2 (K562) model compared to those learned directly directly mouse *Rbfox*1/2/3 triple knockout data. Source data

Knockdown and crosslinking experiments are specific to the cell types and conditions in which the experiment was performed; however, if a model successfully captures regulatory activity, its predictions should generalize to other biological contexts. To assess whether our models can generalize in this way, we considered SFs with knockdowns in distinct cell types and compared their predictions. For 12 of the 14 factors, models inferred from HepG2 and K562 cells yielded highly similar predictions of regulation (Fig. 3f), indicating that KATMAP consistently identifies the same underlying relationship between binding motifs and splicing regulation. To assess whether this generalizability extends across species, we applied KATMAP to mouse *Rbfox1/2/3* triple-knockout data^51^. The inferred activity map (Extended Data Fig. 5a) was highly consistent with those obtained from human RBFOX2 knockdowns (Fig. 2e and Extended Data Fig. 5b). Using the human RBFOX models to score exons in the mouse transcriptome predicts essentially the same regulation as the splicing impacts learned directly from mouse neuronal *Rbfox1/2/3* triple-knockout data^51^ (Fig. 3g and Extended Data Fig. 5c). Together, these analyses indicate that the models KATMAP infers can be applied to different tissues and to related species, including those where in vivo binding data are lacking and where genes and exons not expressed in the original knockdown experiment are present.

### KATMAP identifies *cis*-regulatory elements and the consequences of their disruption

**Fig. 4:**
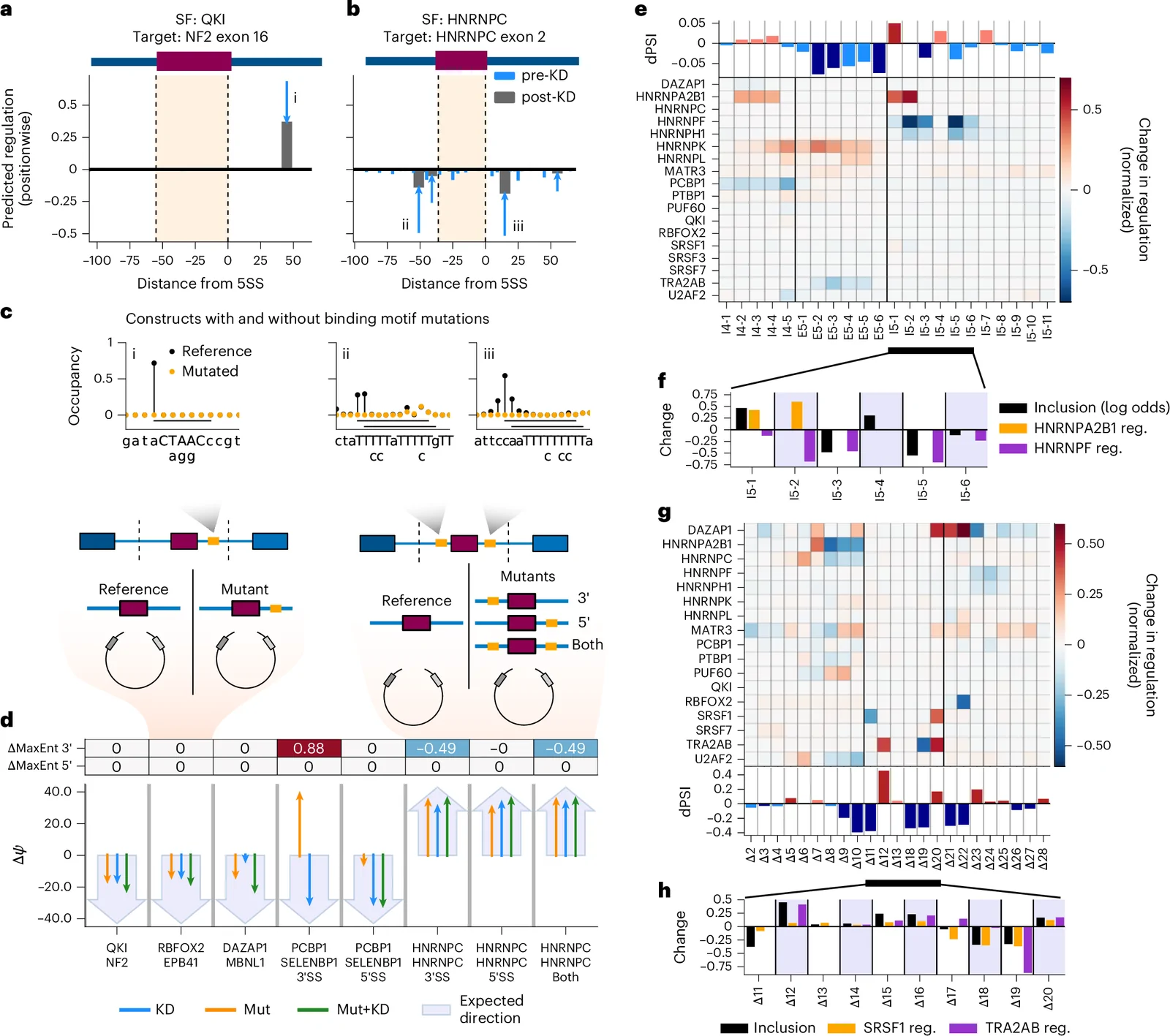
KATMAP predicts the consequences of disrupting *cis*-elements. **a**,**b**, The expected changes in regulation because of loss of QKI (**a**) and HNRNPC (**b**) binding at KATMAP’s predicted targets, assuming that change in free protein was proportional to knockdown efficiency. The blue arrows indicate the change in regulation from pre-knockdown (tail of arrow) to post-knockdown (gray bar) levels. **c**, Schematics of the minigene reporter design. Top, the expected occupancy at the predicted SREs (i–iii from **a**) and the predicted effects of mutations. The reference sequence is on the *x* axis with the expected binding motif capitalized; the specific mutations are below. Bottom, schematics of the reporters constructed from these sequences. **d**, The results of the reporter assay. The thin arrows depict the observed effect of knockdown, mutation and mutation + knockdown. The large arrow depicts direction the mutations were expected to alter inclusion. **e**, The reported changes in HRAS exon 5 inclusion (top) after ASO treatments and the changes in SF regulation predicted by KATMAP (bottom). Changes in regulation are normalized to the highest activity position in each SF’s activity map. Black vertical lines separate the exon from the introns. **f**, Zoomed-in view of altered inclusion and regulation by HNRNPF and HNRNPA2B1 in the downstream intron. **g**, The reported effects of deletions on inclusion of the TRA2B poison exon (bottom) and KATMAP’s predicted changes in regulation (top), as in **e**. **h**, Zoomed-in view of the effect of deletions in the exon on inclusion and predicted regulation by TRA2A/B and SRSF1 based on the 200-nt exonic models. Source data

Implicit in KATMAP’s predictions are statements about *cis*-regulatory elements: for any predicted target, we can examine the collection of predicted SREs that could impact that exon’s inclusion. For example, QKI is predicted to enhance the inclusion of *NF2* exon 16, which is one of two stop-codon-containing exons in the gene, the skipping of which suppresses tumor growth 3–4-fold in schwannoma^52^. Using KATMAP’s outputs, the position-wise splicing impacts can be computed, which suggest that QKI enhances the exon’s inclusion through a single ACUAA motif 53 nt downstream of the 5SS (Fig. 4a). Other targets are predicted to be controlled by multiple binding sites. For example, HNRNPC is predicted to suppress the inclusion of a highly conserved exon in its own 5′ UTR through multiple poly(U) motifs in both the upstream and downstream introns (Fig. 4b).

We used this interpretability to validate selected predictions using a minigene splicing assay. For five SFs (QKI, RBFOX2, DAZAP1, PCBP1 and HNRNPC), we chose predicted target exons that changed in splicing upon knockdown and evaluated the effects of mutating their predicted SREs (Supplementary Table 5). For predicted targets with both upstream and downstream SREs, we considered the effects of mutating only the upstream or only the downstream elements. We inserted the reference and mutated exons into reporters and measured exon inclusion (Fig. 4c). To confirm that the effects of mutation were mediated by the expected SF, we assayed reporters with and without knockdown of the expected factor.

Inclusion changed in the expected direction for seven of the eight mutant reporters (Fig. 4d). Mutation of the QKI-binding and RBFOX2-binding sites downstream of the *NF2* and *EPB41* exons, respectively, reduced inclusion comparable to knockdown. Disrupting the DAZAP1-binding sites upstream of the *MBNL1* exon decreased inclusion as expected, to a greater extent than the knockdown. Mutation of the PCBP1 downstream sites decreased inclusion moderately as expected but mutation of the upstream PCBP1 sites increased rather than decreased inclusion. In this case, the upstream mutations are close to the 3SS and are predicted to increase the strength of the PPT, which may override any impact of the loss of PCBP1 binding. In the HNRNPC reporters, both the upstream and downstream HNRNPC mutations behaved as expected, with either being sufficient to increase inclusion to nearly 100%. Thus, with the exception of one of the PCBP1 mutants, splicing changes always occurred in the expected direction.

**Extended Data Fig. 6:**
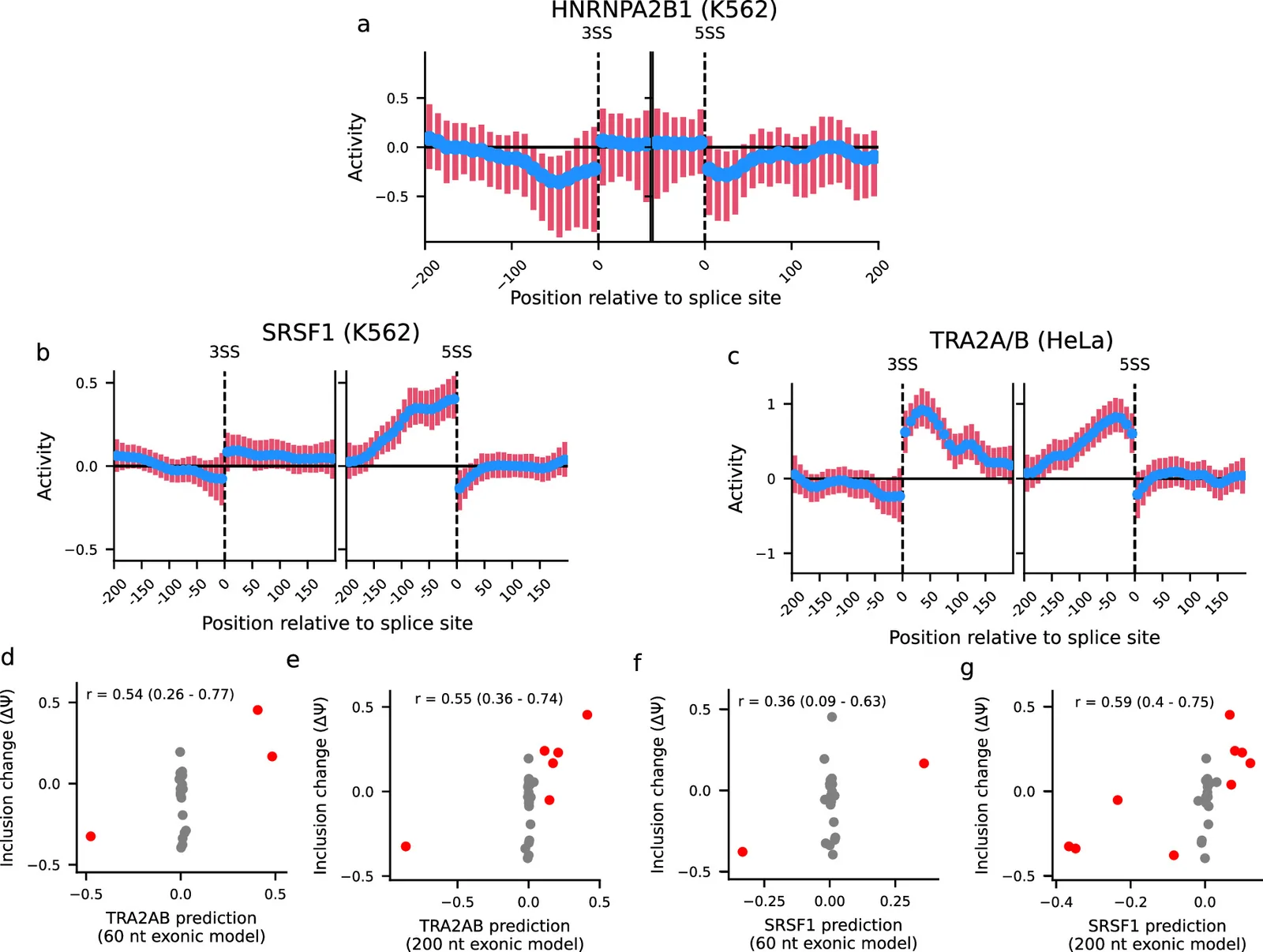
Additional activity maps predictive of inclusion changes in the ASO and deletion tiling analyses. **a**-**c**) The activity map (with 94%-CI, n=1,154 posterior samples) from the HNRNPA2B1 (K562) ENCODE knockdown (**a**) using the standard 60 nt exonic window, and activity maps inferred from **b**) SRSF1(K562) and **c**) TRA2A/B knockdowns, with the windows expanded to consider positions 200 nt up- and downstream of splice sites, with 94%-CIs (n=982 and n=1,248 posterior samples respectively). **d**-**g**) Association between change in exon inclusion^54^ and KATMAP predicted changes in TRA2A/B and SRSF1 regulation based on the standard activity maps (**d**, **f**) and extended 200 nt activity maps (**e**, **g**). Deletions that change KATMAP’s prediction by more than ± 5% of the largest possible change are colored red. 94%-CI for the correlations were computed using Bayesian bootstrapping (n=1,000 posterior samples). Source data

To further evaluate KATMAP’s *cis*-element predictions, we asked whether our models could be used to interpret two previously published tiling assays: an ASO tiling of *HRAS* exon 5 (ref. ^53^) and a deletion tiling of *TRA2B*’s poison exon^54^. The *HRAS* exon tiling experiment found that ASOs targeting the downstream intron led to decreases in exon inclusion by blocking enhancing activity of HNRNPF/H proteins at G_3_ and G_4_ motifs^53^, as well as increases in inclusion consistent with a previously reported silencing element just downstream of the 5SS, likely mediated by HNRNPA1 (ref. ^55^). While the HNRNPF and H1 knockdowns both yielded significant KATMAP activity maps, the HNRNPA maps failed to pass our model comparison criteria. However, the HNRNPA2B1 (K562) knockdown yielded an activity map supporting intronic repression (Extended Data Fig. 6a) and *HRAS* exon 5 was the model’s highest confidence repression target; thus, it was included in these analyses.

For each SF, we predicted the changes in regulation because of ASOs blocking SREs, assuming that the ASOs disrupt recognition of their complementary nucleotides and one additional base on either side. The SFs predicted by KATMAP to be most strongly affected by the ASOs were HNRNPF and HNRNPA2B1, with HNRNPH1 showing a profile similar to HNRNPF (Fig. 4e). Two of the three ASOs predicted to disrupt HNRNPF’s enhancing activity led to decreased inclusion and four of the five ASOs predicted to disrupt HNRNPA2B1’s repression led to increased inclusion (Fig. 4e,f). The exception was ASO I5-2, which was predicted by KATMAP to disrupt both HNRNPA2B1’s repression and HNRNPF’s enhancement and yielded no change in inclusion (Fig. 4f), perhaps because blocking both activities canceled each other out. The ability of the HNRNPA2B1 model to explain the effects of ASOs both suggests that it captures real regulatory activity and that our model comparison criterion might be excessively stringent in some cases.

Analysis of a deletion tiling experiment of the *TRA2B* poison exon again implicated expected factors. KATMAP primarily explains the effects of exonic deletions on inclusion in terms of the SR proteins TRA2A/B and SRSF1 (Fig. 4g). While deletions that increase inclusion are typically interpreted as removing silencing elements, KATMAP instead predicts that these deletions promote positive regulation by TRA2A/B by moving its binding sites closer to the SSs, where activity is higher. The *TRA2B* poison exon is particularly long (276 nt), while our activity maps only extend 60 nt from the SSs. To confirm that this was not driven by binding sites located just outside of this 60-nt window, we refitted SRSF1 and TRA2A/B models using larger windows that extend up to 200 nt into the exon (Extended Data Fig. 6b,c). These extended models infer that TRA2A/B and SRSF1 activity decays with distance from the SSs, as previously demonstrated for SR proteins^45^, and similarly predict that the deletions increase regulation by moving binding sites nearer the SSs (Fig. 4h and Extended Data Fig. 6d–g). Finally, we performed a permutation analysis, exchanging *ΔΨ* among deletions within the exon and within the upstream and downstream introns. The observed associations for TRA2A/B and SRSF1 were consistently stronger than the chance associations under permutation (*P* = 0.003 and *P* = 0.007, respectively).

Together, these observations support KATMAP’s ability to infer *cis*-regulatory elements responsible for splicing regulation and to predict the effects of their disruption. KATMAP’s predictions should, therefore, aid in predicting viable ASO targets, reducing the search space needed for screening.

### Target predictions reveal cooperative regulation

Our model makes the simplifying assumption that SFs act independently of each other. However, we reasoned that we could uncover evidence of cooperative regulation by examining our model’s predictions for deviations from this null assumption. To test this possibility, we focused on RBFOX and QKI, which have strikingly similar activity maps despite binding distinct motifs (Fig. 2e,f), raising the possibility of a functional relationship between them. Earlier analyses noted that the sets of exons responsive to QKI and RBFOX knockdown are overlapping^24,56,57^ and observed enrichment of QKI eCLIP signal at RBFOX-downregulated exons. However, clear evidence of QKI and RBFOX binding at the same exons was not observed and it was concluded that the correlated effects of knockdown did not reflect direct coregulation by the factors^24^, though another study found binding of both factors at a subset of exons^57^. These changes could arise without direct coregulation (for example, if perturbing RBFOX impacted QKI activity, altering splicing of its targets). These analyses, however, could not distinguish between the direct and indirect effects of knockdown, a limitation that might have obscured signatures of coregulation.

**Fig. 5:**
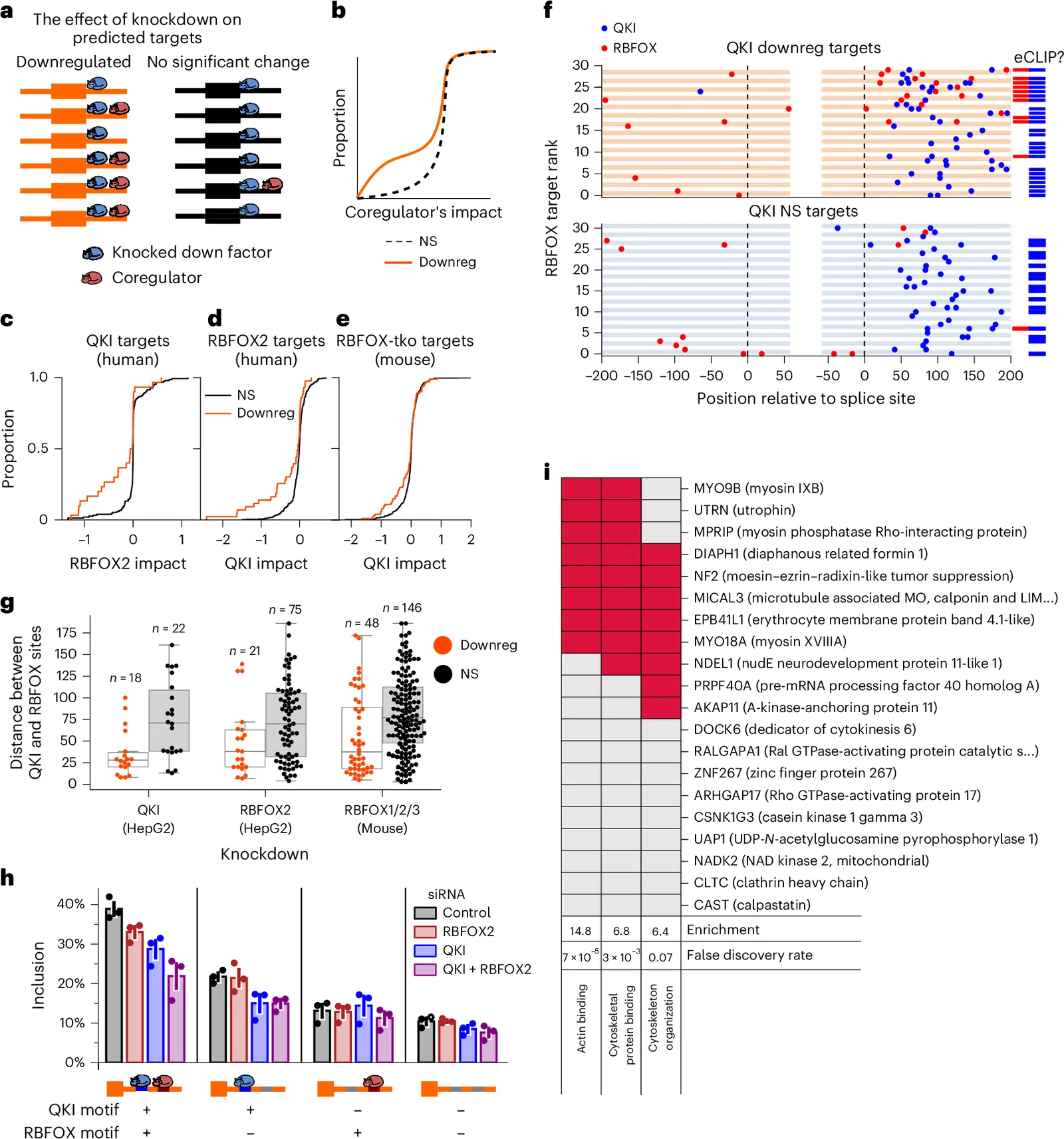
QKI and RBFOX cooperatively regulate a splicing subprogram. **a**, In the case of cooperativity, predicted targets that affected knockdown should be enriched for the coregulator compared to those unaffected by knockdown. **b**, The expected distributions of the coregulator’s splicing impact computed at KD-affected (orange) and KD-unaffected targets (black). **c**–**e**, Distributions of RBFOX2 (**c**) and QKI (**d**,**e**) splicing impacts at the targets of the other protein (**c**, QKI; **d**,**e**, RBFOX), comparing the KD-affected (orange) to KD-unaffected targets (black). **f**, Locations of predicted binding sites (∣relative change in binding∣ > 20%) for both RBFOX (red) and QKI (blue) in KD-affected QKI enhancing targets affected (**top**) and an equal number of KD-unaffected targets (**bottom**). The exons are sorted by RBFOX splicing impact and the bars to the right denote the presence of eCLIP peaks in the downstream intron. **g**, The distributions of distances between the nearest QKI and RBFOX sites in KD-affected (orange) and unaffected (black) targets. Boxes are centered on the median and span the lower and upper quartiles, with whiskers extending to the most extreme observation no more than 1.5× the IQR distant from the lower or upper quantile. **h**, Semiquantitative RT–PCR measurements of NF2 exon 16 inclusion (mean ± 1 standard error) in minigene for constructs with the QKI and/or RBFOX motifs mutated under all combinations of RBFOX2 and QKI knockdown. **i**, GO analysis of confident QKI–RBFOX cotargets; red denotes GO membership. Source data

We used KATMAP’s ability to discriminate direct regulatory targets from indirect effects to reassess the case for direct coregulation. In the scenario of strong cooperativity, knocking down either factor should primarily affect exons bound by both factors, whereas exons bound by only one should be minimally affected. As KATMAP models one SF at a time, an exon with a binding motif for the knocked down factor will be called a target, whether or not a motif for the coregulator is present. Cooperative regulation should produce a clear signature at predicted targets: knockdown-affected targets of each factor should be enriched for targeting by both coregulators compared to targets unaffected by knockdown (Fig. 5a,b). We detected this expected signature of cooperativity, with differentially spliced QKI targets being enriched for RBFOX enhancing regulation (Fig. 5c). The reciprocal relationship was also observed; predicted RBFOX2 targets affected by RBFOX2 knockdown were enriched for QKI-binding sites (Fig. 5d). Furthermore, this pattern was evident in mouse cells depleted of all three RBFOX paralogs (*Rbfox1/2/3*) (Fig. 5e), supporting that coregulation of exons by RBFOX and QKI proteins is conserved in mice (~70 million years ago).

These coregulated exons have both QKI-binding and RBFOX-binding sites (Fig. 5f), frequently multiple of each, occurring as clusters of interspersed motifs separated by a few dozen nucleotides (Fig. 5g). This proximity of binding sites suggests that QKI and RBFOX proteins could physically interact, which is supported by prior yeast two-hybrid^58^ and pulldown mass spectrometry data^59^. Furthermore, reassessment of the eCLIP data focused on the predicted direct targets supports binding of both QKI and RBFOX to the same exons; QKI crosslinking was detected at both knockdown-affected and unaffected QKI targets, whereas RFBOX2 was primarily bound at the targets affected by QKI knockdown. We selected *NF2* exon 16 to directly test this, constructing reporters with the native QKI-binding and RBFOX-binding motifs mutated in all combinations and performed all combinations of QKI and RBFOX2 knockdown. We found that regulation of this exon by RBFOX2 depends on the presence of QKI’s motif; while knockdown of RBFOX2 or the mutation of its motif decreased inclusion, they had no additional effect on inclusion when the QKI motif was absent (Fig. 5h). Together, these observations support direct, cooperative coregulation of exons by QKI and RBFOX proteins.

Our analyses identified 20 human exons with binding sites for both QKI and RBFOX proteins and responsiveness to either QKI or RBFOX2 knockdown (Supplementary Table 3). Of these 20, 11 are in genes involved in cytoskeleton binding and organization, with seven being actin-binding proteins (Fig. 5i). This represents strong enrichment for these categories relative to expression-matched control genes, as well as relative to the full set of RBFOX or QKI targets, consistent with a focused subprogram active only in cells where both QKI and RBFOX are expressed and distinct from their individual regulatory roles. Cytoskeletal remodeling and cell migration are integral to early neuronal development and a number of these exons occur in genes with known roles in neural and glial biology. For example, *NDEL1* is critical for cell migration during neural differentiation^60^ and skipping of *NF2* exon 16 strongly promotes growth of glial tumors^52^. Both RBFOX and QKI proteins are expressed in neuronal progenitor cells, which differentiate into both neurons (where RBFOX predominates) and glia (where QKI predominates), suggesting a potential venue for coregulation^49,61^.

### KATMAP’s models uncover the SFs responsible for splicing changes in RNA-seq data

**Fig. 6:**
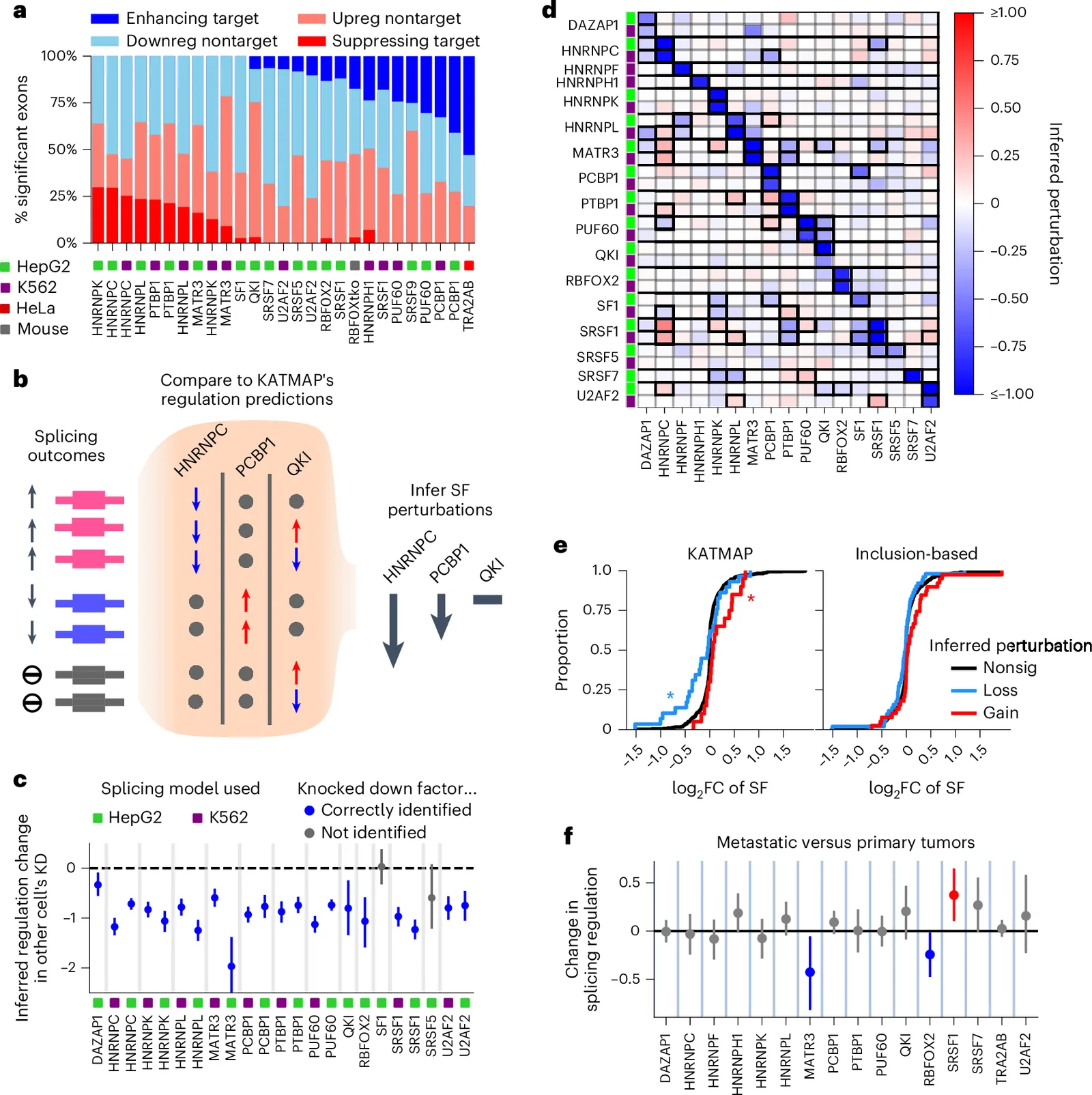
KATMAP’s regulatory models uncover SF perturbations. **a**, The fraction of targets and nontargets among differentially spliced exons (rMATS FDR < 0.05). Ambiguous target predictions (Pr(target) and Pr(nontarget) < 0.8) are not included. **b**, Schematic showing how we infer underlying SF perturbations by comparing splicing changes to KATMAP’s regulatory models. In this example, exons predicted to be suppressed by HNRNPC are upregulated and exons enhanced by PCBP1 are downregulated, suggesting that decreases in HNRNPC and PCBP1 regulation caused the splicing changes. There is no association between the splicing changes and QKI’s predicted targets, suggesting no perturbations to QKI’s regulation. **c**, Predicted changes in regulation for the knocked down factors (*γ*^(*f*)^, as described in the Methods), with the 94% CI (*n* = 800 posterior samples). All activity models learned from the other cell type were included in the regression and knockdowns where the expected factor gave the strongest signal are indicated in blue. **d**, Predicted regulation changes for knockdowns of all RBPs with a significant activity map, using ashr to enforce sparsity. Changes significant at 10% FDR are denoted with thick boxes. **e**, The log_2_ fold changes in expression (DEseq, ashr shrinkage estimates) of SFs inferred to have been secondarily perturbed at a 10% FDR. *Significant according to a two-sided KS test at *P* < 0.05 (*P* = 0.007 for losses and 0.01 for gains). **f**, Predicted differences in SF regulation between metastatic and primary PDA samples, with 94% CI (*n* = 800 posterior samples). Source data

Most knockdown experiments cause abundant splicing changes in both directions, even for established splicing activators (for example, SRSF1) and repressors (for example HNRNPL), where direct targets should be downregulated and upregulated, respectively, limiting their ability to predict causal factors. Indeed, in most ENCODE knockdowns, fewer than 30% of the significant splicing changes were predicted by KATMAP to be direct targets (Fig. 6a). Given that our previous analyses (Fig. 3c–e) showed that predicted nontargets are depleted for eCLIP peaks and typically display smaller inclusion changes, this suggests that many of the remaining splicing changes do in fact reflect indirect effects, potentially resulting from the multiday period of antibiotic selection common to knockdown experiments and the extensive crossregulation among SFs. Because KATMAP distills knockdown and RNA-seq data into transcriptomewide predictions about direct SF regulation, it can be applied to interpret splicing changes in other RNA-seq datasets following perturbations. This includes the indirect effects of knockdown, as well as the splicing changes observed following stress, drug treatments, cell differentiation and in clinical samples associated with disease.

If a given SF contributed to the splicing changes in an RNA-seq dataset, the splicing model learned from analyses of its knockdown data should be predictive of which exons were affected. We express this logic with a multinomial logistic regression that predicts each exon’s splicing changes on the basis of the splicing impacts computed using the models we learned from the different ENCODE knockdowns (Fig. 6b). To validate this approach, we used the splicing models inferred from K562 knockdowns to explain splicing changes in the HepG2 knockdowns and vice versa. This approach correctly identified the depleted SF as most predictive in 21 of 23 knockdowns (91%) (Fig. 6c), demonstrating that our regulatory models can generalize beyond their training data to infer the SFs underlying transcriptomic changes across cell types.

**Extended Data Fig. 7:**
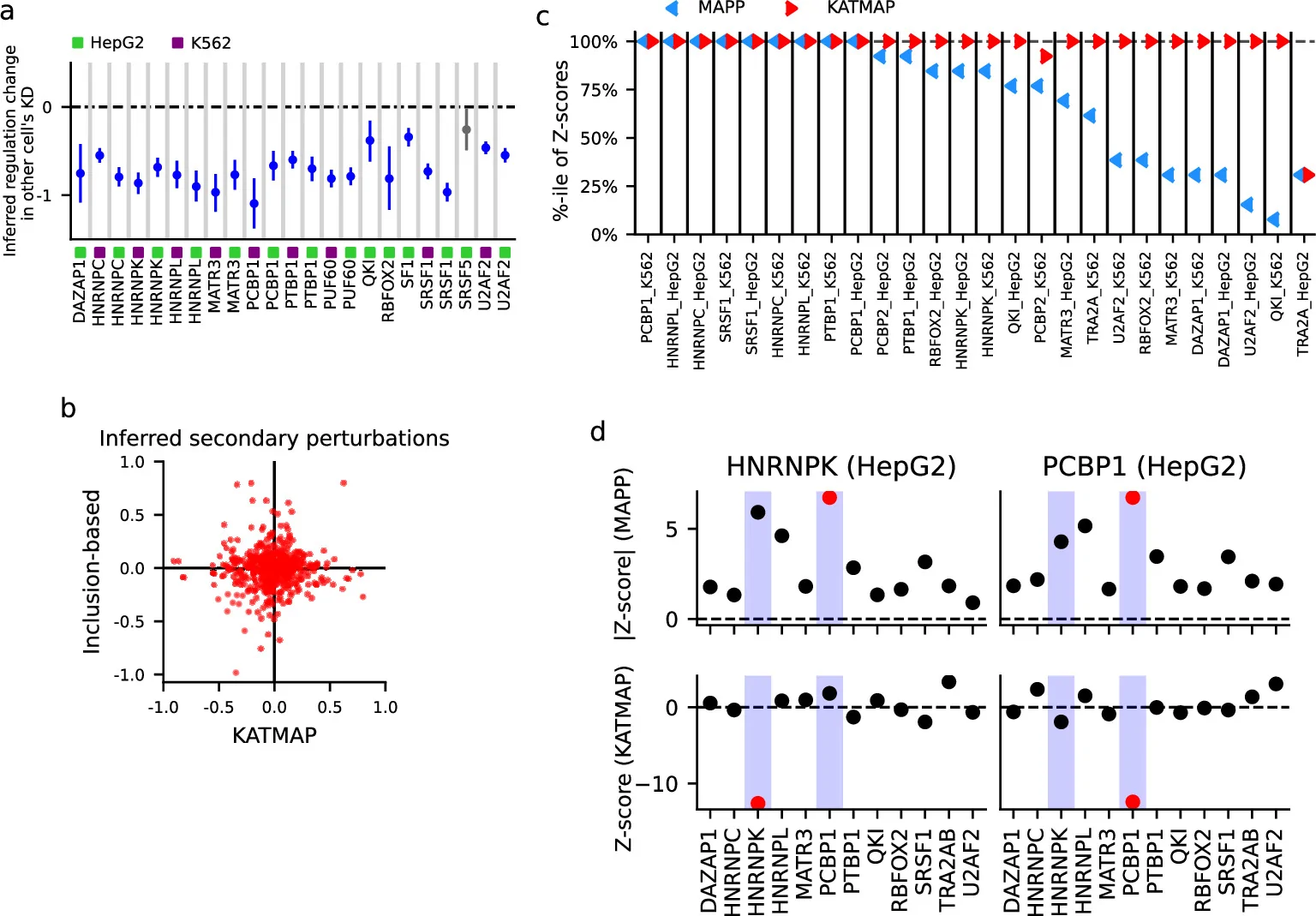
Comparison of KATMAP to alternative approaches to inferring SF perturbations. **a**) Predicted changes in regulation (with 94%-CI, n=800 posterior samples) for the knocked down factors, inferred using observed inclusion changes as the predictors. Knockdowns where the expected factor gave the strongest signal are indicated in blue. **b**) Scatterplot comparing the secondary perturbations (that is excluding the knocked down SF) inferred by the inclusion-based approach to those inferred by KATMAP. **c**) The rank of the z-score assigned to the knocked down factor by MAPP (blue) and KATMAP (red) for the 26 knockdowns where either MAPP or KATMAP identified evidence of splicing activity. Analysis was restricted to factors knocked down in both HepG2 and K562 to ensure that when inferring perturbations with KATMAP the model learned from the other cell type could be used. **d**) The SF perturbations inferred by MAPP (top) and KATMAP (bottom) in HNRNPK and PCBP1 knockdowns. The SF with the largest z-score is colored red, and HNRNPK and PCBP are highlighted for reference. Source data

We asked how this approach compares to using the exon inclusion changes directly observed in other knockdown data as predictors to infer perturbations. Both KATMAP and the inclusion-based approach effectively identified the knocked down factor (Fig. 6c and Extended Data Fig. 7a). However, the two approaches disagreed as to which SFs were secondarily perturbed (Extended Data Fig. 7b). To assess these disagreements, we selected significant secondary perturbations (10% false discovery rate (FDR), Fig. 6d) and asked whether these were associated with expression changes in the expected directions. The gains and losses of SF regulation inferred by KATMAP were supported by corresponding increases and decreases in expression (Fig. 6e, Kolmogorov–Smirnov (KS) statistics = 0.35 and 0.31; *P* = 0.01 and 0.007), while the gains inferred by the inclusion-based approach were more weakly supported by expression increases and not supported for inferred losses (KS statistics = 0.21 and 0.19; *P* = 0.07 and 0.06). These observations indicate that KATMAP more accurately identifies secondarily perturbed factors, likely because of its ability to focus primarily on splicing changes at the respective SF’s direct targets.

MARA (motif activity response analysis)-like approaches have previously been applied to this problem; thus, we compared KATMAP to the MARA-like tool, MAPP (motif activity on pre-mRNA processing)^13^. MAPP considers a large library of RNA-binding protein (RBP)-associated PWMs or *k*-mers and fits linear models to identify which motifs are associated with splicing changes, simultaneously estimating impact maps that describe position-specific activity and computing absolute *z* scores indicating which SFs might be responsible for the splicing changes. To compare the two approaches, we considered SFs for which there were (1) knockdowns in both ENCODE cell lines; (2) PWMs in the MAPP library; and (3) a significant KATMAP model in at least one knockdown.

MAPP assigned the top *z* score to a PWM associated with the knocked down factor (or a paralog) in nine of the 24 knockdowns, while KATMAP did so in 22 of 24 (Extended Data Fig. 7c). Furthermore, because MARA-like approaches must learn the activities associated with each motif every time they are applied, MAPP struggled to distinguish factors with similar binding motifs. For example, in the HNRNPK knockdowns, MAPP identified perturbations of both PCBP1 and HNRNPK because the two SFs bind similar C-rich motifs (Extended Data Fig. 7d) and faced the same problem in the PCBP1/2 knockdowns. By contrast, KATMAP couples motifs with activity profiles previously learned from knockdown data, allowing it to distinguish between HNRNPK and PCBP1 perturbations (Extended Data Fig. 7d) because their distinct patterns of activity are accounted for (Fig. 2a). This highlights the added power gained from incorporating prior knowledge of an SF’s activity when it is available.

These analyses provide a proof of principle that SF perturbations can be inferred using KATMAP’s models in the context of well-controlled experiments. To assess the potential of this approach in more complex and disease-relevant contexts, we applied KATMAP to a published comparison of splicing changes between primary and metastatic pancreatic ductal adenocarcinoma (PDA) samples, which uncovered a role for RBFOX proteins in repressing metastasis^62^. Unlike knockdown data, cancer samples typically represent a mixture of distinct etiologies, which may obscure the signal of any specific causal factor. Indeed, the original analysis did not recover unambiguous RBFOX motifs, but rather amalgams of G-rich and C-rich motifs, some of which had similarity to RBFOX secondary motifs. Applying KATMAP, we directly inferred loss of RBFOX regulation in the metastatic samples through a single analysis (Fig. 6f), without the need to first identify motifs and then match them to putative SFs. We further infer a gain of SRSF1 regulation, a factor known to promote tumorigenesis^3^, which was missed in the original motif analysis. The inferred perturbations to RBFOX and SRSF1 are consistent with reported protein level changes in these samples, with RBFOX protein levels decreased in the metastatic lineages and SRSF1 protein levels elevated^62^. This analysis highlights KATMAP as a powerful tool for inferring the causal factors for splicing changes in disease contexts.

## Discussion

Since the development of RNA-seq, perturbation experiments have been the predominant tool for probing the rules by which regulatory factors control gene expression and processing. However, these studies typically report visual summaries of regulation, which are not directly useful for quantitatively interpreting regulation in additional biological settings. Furthermore, most analyses do not distinguish between the direct and indirect effects of perturbation, instead assuming that the signal from direct targets will rise above the background of indirect effects. Our approach, KATMAP, extracts actionable insights about splicing regulation from SF perturbation data. The activity maps that KATMAP generates are interpretable visualizations of an SF’s regulatory activity and also models that can be applied to make predictions in additional contexts. These predictions distinguish the direct from indirect effects of SF perturbation, allowing indirect effects to be further interpreted in terms of secondary factors and preventing them from contaminating conclusions drawn about the perturbed factor’s biological role. Our approach is supported by the reproducibility of inferred activities and predictions when applied across cellular contexts and related species. The model design makes KATMAP a general framework for rigorously applying insights from perturbation data to various types of analyses.

Despite these advantages, our approach has several limitations. Firstly, we make the simplifying assumption that each SF can be modeled individually and ignore potential cooperative regulation between SFs. Nonetheless, the regulatory models learned by KATMAP can be used as a null hypotheses to uncover evidence of cooperativity, as seen for RBFOX and QKI proteins. Secondly, KATMAP’s interpretability depends on knowledge of the SF’s sequence specificity. While motifs discovered de novo can be used with KATMAP, there are epistemic limitations to what can be concluded when an SF’s binding motif is not known a priori. As the sequence specificity of more SFs becomes available, the set of factors for which interpretable models can be learned will expand. Lastly, while based on a general model of regulation, KATMAP is currently limited to describing splicing regulation. Recent work indicates that transcription factors also have position-specific activities^63^, suggesting that modified versions of KATMAP might find application in modeling transcription.

In distilling knockdown experiments into generalizable models, KATMAP provides the opportunity to leverage decades of experimental work to answer biological questions. The insights our model extracts from perturbation data will benefit work focused on specific regulatory factors, providing information about activity and targets and quickly generating hypotheses to further probe that factor’s biology. More generally, many researchers who generate RNA-seq data will perform a differential splicing analysis, typically uncovering hundreds of splicing differences, which are challenging to interpret. Integrating KATMAP into RNA-seq workflows provides a means to focus downstream analyses on factors responsible for the phenotype of interest. Our reanalyses of pancreatic cancer data illustrate a use case for inference of SF drivers of splicing changes in a clinical dataset (Fig. 6f). Identifying the SFs and SREs underlying splicing programs in disease may speed the search for therapeutic targets and lead to a better understanding of underlying causes. Furthermore, because KATMAP’s predictions are explicitly framed in terms of changes in binding at *cis*-elements, our models can facilitate the interpretation of sequence variants. This is highlighted in our minigene experiments where we successfully identified regulatory sequence elements and designed mutations that disrupt that regulation. Similar analyses have applications in ASO design by providing information on which *cis*-elements are likely to change inclusion in the desired direction. Focusing on these regions can reduce the need for laborious tiling or deletion assays during therapeutic development and inference of specific SFs may aid in the design of ASOs active in relevant tissues.

## Methods

### Selecting the knockdown experiments

We selected ENCODE knockdown RNA-seq experiments for proteins annotated as SFs^24^ and which had a good affinity model derived from RBNS^58^ using RBPamp^26^ or a PWM derived from RNAcompete^27^ obtained from the CISBP-RNA data database^28^. We use the rMATS differential splicing^18^ results for the hg19 human reference genome in our analyses. The mouse *Rbfox1/2/3* triple-knockout data^51^ were processed by rMATS 4.1.1 with default settings using the GRCm39 assembly.

### The structure of the KATMAP regression model

The goal of KATMAP is to infer an SF’s regulatory activity and targets from knockdown data by explaining the resulting splicing changes in terms of changes in SF binding. Accomplishing this requires several inputs. Firstly, we need the results of a differential splicing analysis, which assigns labels *Y* to exons indicating whether their inclusion increased, decreased or was unaffected by knockdown. These are the observations KATMAP seeks to predict. The second input is a model of the SF’s sequence specificity. This assigns exons in the differential splicing analysis a set of scores, *S*, which evaluate all potential binding sites within predefined windows around their SSs. These scores, *S*, are what KATMAP will use to explain the splicing changes, *Y*, in terms of SF binding and ultimately to infer regulatory activity. Lastly, additional information about each exon can be included using a set of optional linear predictors, *X*. In our analyses, we use these linear features to control for expression and pre-knockdown exon inclusion levels.

#### Defining the splicing changes

Any differential splicing analysis that labels each exon as unchanged, upregulated or downregulated may be used to define the observations, *Y*. We use one-hot encoding to define *Y*_*i*_ as (1, 0, 0), (0, 1, 0) or (0, 0, 1) if exon *i* is labeled downregulated, unchanged or upregulated, respectively.

In our analyses, we defined splicing changes by further processing the rMATS outputs with ashr^64^ to better account for the uncertainty in rMATS’s estimates. This step had two motivations. First, ashr accomplishes something like a multiple-test correction on parameter estimates, while accounting for the possibility that more exons are upregulated than downregulated (or vice versa). Second, while KATMAP does not consider the magnitude of splicing changes, some of our downstream analyses do. The shrinkage estimates provided by ashr control for measurement error because of low read counts and yield a cleaner view of splicing differences than the raw rMATS *Δψ* summaries.

Briefly, we used the read counts and rMATS *P* values to reconstruct the effect size on a log odds scale, which we denote *Δϕ*, and associated uncertainty (mathematical details in Supplementary Information). We then pass these values to ashr to obtain shrinkage estimates for *Δϕ* and associated s.d. We construct 94% CIs on the basis of these, calling exons differentially spliced if the 94% CI does not overlap zero and nonsignificant otherwise.

#### Defining the training set

We primarily use the rMATS skipped exon table in our analyses. If a set of skipped exons partially overlapped or shared flanking exons, we retained only the most significant to avoid double counting. We include all remaining significant exons in our training sets. For computational efficiency, we downsample the nonsignificant exons, retaining 2,000 randomly chosen exons.

#### Scoring motifs with a binding model

To explain splicing in terms of binding, KATMAP requires some model of the perturbed SF’s binding motif. The binding model may be a traditional PWM obtained from RNAcompete, one or more PSAMs (for example, from RBPamp) or more general affinity models. The key requirement is that the binding model orders potential binding sites from weakest to strongest. In our analyses, we used PSAMs derived from RBNS^25^ using RBPamp^26^ when available and PWMs derived from RNAcompete^27^ obtained from the CISBP-RNA database^28^. We excluded RBNS models where the PSAMs showed strong complementarity to the sequencing adaptors, which likely reflects a technical artifact rather than true sequence specificity.

To score a *k*-length motif *x* with a single 4 × *k* PWM or PSAM, *W*, we sum the scores (in log scale) for each nucleotide in the motif

$$S(x,W\;)=\mathop{\sum }\limits_{i=1}^{k}\sum _{{\rm{nt}}\in \{A,C,G,U\}}{W}_{{\rm{nt}},i}[{x}_{i}={\rm{nt}}],$$

where the Iverson bracket [*x* = nt] is equal to 1 if true and 0 if false.

If the motif model comprises multiple matrices *W*_1_, …, *W*_*n*_, each with associated weights *w*_1_, …, *w*_*n*_, then we compute the combined score as

$$S(x)=\log \left({\mathop{\sum }\limits_{i}^{n}}{w}_{i}\exp \left[S(x,W_{i})\right]\right).$$

In practice, we always define the scores such that the optimal motif is assigned a score of 0 and all other motifs receive negative scores. This is accomplished by subtracting the score of the optimal motif from all raw scores.

Even for affinity models, we do not assume that these initial sequence-based scores are fully accurate representations of affinity. Rather, we treat them as related to the log affinity but potentially differing by some scaling factor. To account for this, we include an affinity scale parameter, *λ*, which rescales the binding scores.

a(x)=λS(x)

These rescaled scores are used as log affinities from which occupancy and changes in occupancy are computed. If the scores are truly linearly related to log affinity, this can be thought of as calibrating the scores against the observed splicing changes. If the scores are related to log affinity in a monotonic but not linear manner, this rescaling can be thought of as widening or narrowing the interval of scores to which appreciable changes in occupancy are assigned.

#### Scoring the sequences around exons

KATMAP scores sequences in windows around the SSs of each exon. In our analyses, we scored 200 potential binding sites on the intronic and 60 on the exonic side of the SSs of each cassette exon. If desired, larger or smaller windows can be used or the regions adjacent to the flanking exons’ SSs can be included.

Our model calculates a log affinity for each of the potential binding sites analyzed per exon. For computational tractability, rather than model binding at nucleotide resolution at each exon, we aggregate neighboring binding sites into nonoverlapping regions of ten potential binding sites each. We index these regions, 1, …, *J*, where, in most of our analyses, $J=\frac{1}{10}(200+60)\times 2=52$. More generally, in an analysis with a different region size, different intronic or exonic window size or number of SSs (for example, including the flanking exons),

$$\begin{array}{l}J=\displaystyle\frac{1}{{\rm{region}}\;{\rm{size}}}({\rm{intronic}}\;{\rm{window}}\;{\rm{size}}+{\rm{exonic}}\;{\rm{window}}\;{\rm{size}})\\\quad\;\times {\rm{number}}\;{\rm{of}}\;{\rm{splice}}\;{\rm{sites}}.\end{array}$$

We then assign each subregion *j* of exon *i* a log affinity. We denote the motif scores of the ten binding sites ending in subregion *j* as *S*_*ij*_ and compute the log affinity of the region by first scaling the log affinity of each binding site by *λ* and taking the log of the sum of their exponents:

$${a}_{ij}={\rm{log}}\;{\rm{sum}}\;{\rm{exp}}(\lambda {{{S}}}_{ij}).$$

#### Defining the binding function

For a given free protein concentration *F*, the occupancy of a site with given log affinity, *a*, is described by the Langmuir equation:

$$\Theta (a;F)=\frac{1}{1+\frac{1}{F{e}^{a}}}.$$

**Extended Data Fig. 8:**
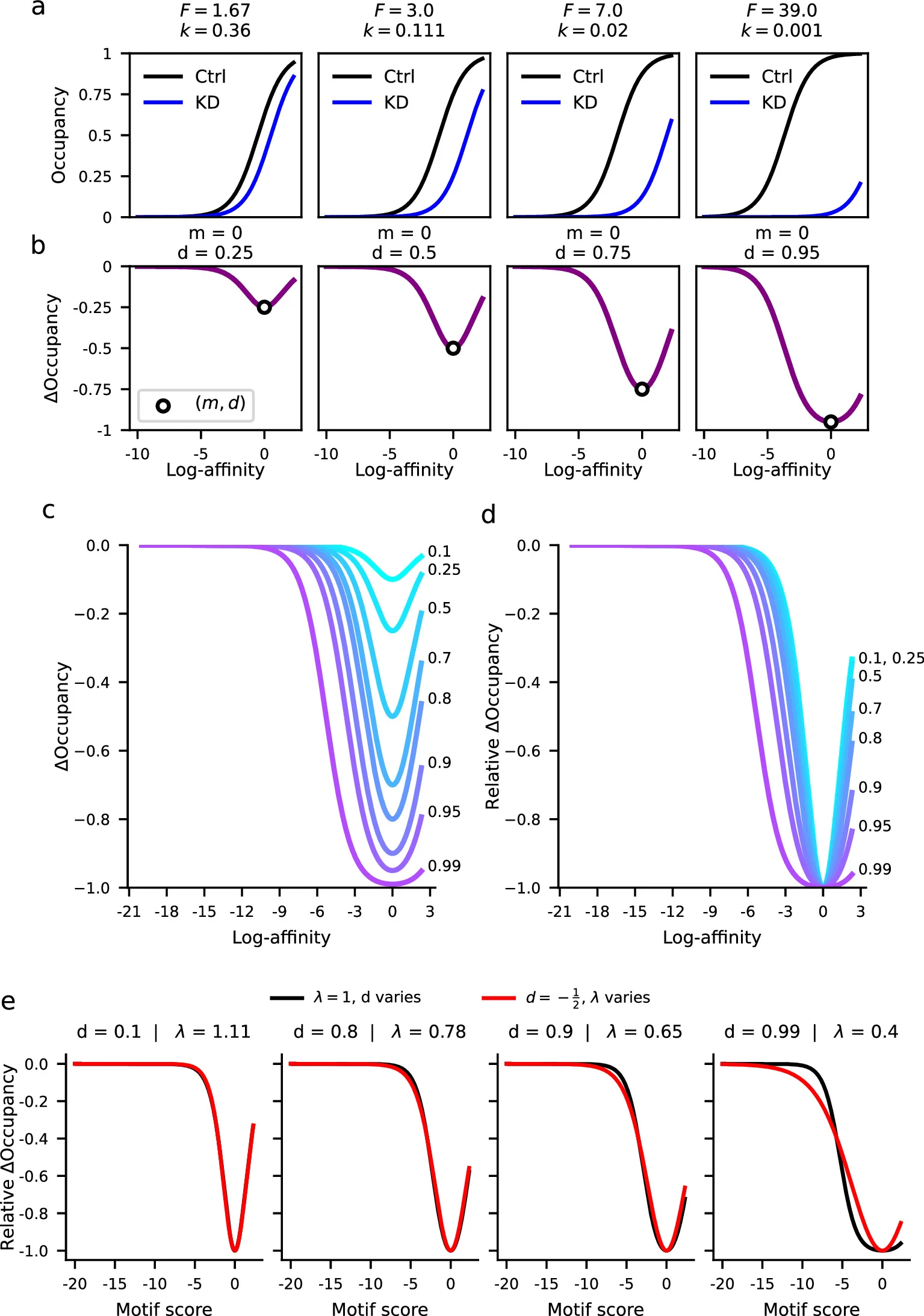
Illustrative examples of how the binding function is defined. **a**) The affinity-occupancy relationship before (black) and after knockdown (blue) for different pre-knockdown free protein concentrations (*F*) and knockdown efficiencies (1 − *k*). **b**) The change in binding functions that result from occupancy curves in **a**, with the corresponding values for the shape parameters *m* and *d*. Note that unlike the biophysical parameters (*F* and *k*) these shape parameters explicitly define the affinity and magnitude of the greatest binding change (open circle). **c**-**d**) Absolute change in binding (**c**) and relative change in binding (**d**) with the most-relevant affinity *m* = 0 for different values of *d*. **e**) The best approximations of the binding function at a given **d** obtainable by rescaling the affinities.

The change in occupancy is, thus, determined by the free protein concentration before and after knockdown or overexpression (Extended Data Fig. 8a). If the free protein concentration changes by some factor *k*, the change in occupancy can be computed (Extended Data Fig. 8b) as:

$$\delta (a;F,k)=\Theta (a;kF)-\Theta (a;F).$$

However, these parameters, *F* and *k*, while biophysically meaningful, are not immediately interpretable in terms of which affinities experience large changes in binding. We, therefore, reparameterize the change in binding function in terms of its shape. First, we include the most relevant affinity, *m*, indicating which affinity experienced the greatest change in binding. Second, we defined the magnitude of the change in binding at sites with affinity *m* as *d* (Extended Data Fig. 8b,c). That is, we reparameterize the function such that the maximum change in occupancy, *d*, occurs at log affinity *m*.

The corresponding biophysical parameters can be computed by solving

$$\begin{array}{rr}{\delta }^{{\prime} }(m;F,k)=0&{\rm{fix}}\,{\rm{the}}\,{\rm{optimum}}\,{\rm{at}}\,m\,{\rm{by}}\,{\rm{setting}}\,{\rm{the}}\,{\rm{derivative}}\,{\rm{to}}\,{\rm{zero}}\\ \delta (m;F,k)=d&{\rm{assert}}\,{\rm{that}}\,{\rm{the}}\,{\rm{change}}\,{\rm{in}}\,{\rm{occupancy}}\,{\rm{at}}\,m\,{\rm{is}}\,d\\ \end{array}$$

which yields

$$\begin{array}{rcl}k(d)&=&\frac{{(d+1)}^{2}}{{(d-1)}^{2}}\\ F(m,d)&=&{\left({e}^{m}\sqrt{k}\right)}^{-1}\end{array}.$$

Thus,

$$\tilde{\delta }(a;m,d)=\delta \left(a;F(m,d),k(d)\right),$$

where $\tilde{\delta }$ denotes a reparameterization of *δ*.

Our model’s predictions are constructed by multiplying these binding changes by other parameters we term activity coefficients, α. Because *d* changes the height of the binding function but not the relative shape (unless *d* is near 1 or −1, $\frac{\widetilde{\delta}(s;m,{d}_{1})}{\widetilde{\delta}(s;m,{d}_{2})}\approx \frac{{d}_{1}}{{d}_{2}}$; Extended Data Fig. 8c), we cannot usually identify both the magnitude of binding changes and the magnitude of regulatory activity; most effects on the model’s predictions of changing *d* from value *d*_1_ to *d*_2_ can be entirely reversed by scaling the magnitudes of the activity coefficients by $\frac{{d}_{1}}{{d}_{2}}$. To resolve this, we normalize the changes in binding by ∣*d*∣ to compute the relative change in binding (Extended Data Fig. 8d).

$${\tilde{\delta }}_{\rm{rel}}(a;m,d)=\frac{1}{| d| }\tilde{\delta }(a;m,d)$$

Furthermore, because all values of ∣*d*∣ < 0.95 yield essentially the same relative changes in binding, we fix $d=-\frac{1}{2}$ for knockdown experiments and $d=\frac{1}{2}$ for overexpression. If ∣*d*∣ > 0.95, as might be the case for knockout data, the relative change in binding function flattens out, but this can be approximated with small values of the affinity scale parameter, *λ* ≪ 1 (Extended Data Fig. 8e).

To recap, we score the sequences around each exon’s SSs using a model of the RBP’s binding motif. We then divide the sequence around each exon into nonoverlapping 10-nt regions, each containing ten potential binding sites. We denote the set of all scores as *S*, where *S*_*ij*_ refers to the scores of the ten potential binding sites in region *j* of exon *i*. We then scale these scores with an affinity scale parameter, *λ*, and treat the resulting values as log affinities. We compute the log affinity of the regions on the basis of the affinities of the ten binding sites contained in each. Finally, we assign each region a relative changing in binding, determined by the value of the most relevant affinity, *m*. We describe this entire procedure of transforming the motif score to relative changes in binding with the binding function

$$B({{{S}}}_{ij};\lambda ,m,d)={\tilde{\delta }}_{rel}\left({\rm{log}}\;{\rm{sum}}\;{\rm{exp}}(\lambda {{{S}}}_{ij});m,d\right),$$

fixing $d=-\frac{1}{2}$ for knockdown experiments.

#### Predicting changes in regulation from changes in binding

We describe the position-specific effect of RBP binding with a set of activity coefficients *α*_1_, …, *α*_*J*_. A positive value of *α*_*j*_ indicates that binding at region *j* enhances exon inclusion. A negative value instead describes suppressing activity. Critically, we assume that activity varies smoothly with position, described later when discussing our model’s priors.

To compute the predicted effect of loss of binding near exon *i* on its splicing outcome, we multiply the change in binding at each region *j*, *B*(*S*_*ij*_; *λ*, *m*, *d*), by the corresponding activity coefficient *α*_*j*_. We make the simplifying assumption that activity is additive across all binding sites; hence, the total change in activity because of the changes in binding across regions 1,...,*J* is

$$\Delta {{{A}}}_{i}={\mathop{\sum }\limits_{j=1}^{J}}B({{{S}}}_{ij};\lambda ,m,d){\alpha }_{j}.$$

#### Connecting changes in regulation to observed splicing changes

To predict the observed splicing changes, the model must assign each exon *i* three probabilities, the probability of being downregulated, unchanged or upregulated: ${\hat{p}}_{i}=({p}_{i}^{(-)},{p}_{i}^{(0)}{p}_{i}^{(+)})$. We describe the log odds ($q=log{\frac{p}{1-p}}$) of these probabilities with an additive model:

$$\begin{array}{rcl}{q}_{i}^{(+)}=\Delta {{{A}}}_{i}+\ldots &=&{\mathop{\sum }\limits_{j=1}}^{J}B({S}_{ij};\lambda ,m,d){\alpha }_{j}+\ldots \\ {q}_{i}^{(-)}=-\Delta {{{A}}}_{i}+\ldots &=&-{\mathop{\sum }\limits_{j=1}}^{J}B({S}_{ij};\lambda ,m,d){\alpha }_{j}+\ldots \end{array}$$

That is, if the activity at exon *i* increases, the predicted log odds of inclusion being upregulated increases and the log odds of being downregulated decreases. In an overexpression experiment, the changes in binding *B*(*S*_*ij*_; *λ*, *m*, *d*) are positive and upregulation is driven by gains in enhancing activity and downregulation by gains in suppressing activity. In knockdown experiments, the changes in binding are negative, flipping the signs of the change in activity: upregulation is driven by a loss of suppressing activity and downregulation is driven by a loss of enhancing activity.

#### Incorporating additional information into the predictions as linear features

Our models aims to explain the results of the differential splicing analysis in terms of SF binding but other information unrelated to binding is likely to be predictive of which exons were called differentially spliced, for biological and/or technical reasons. We incorporate these features by adding a standard linear model into the predictions. The *K* linear features included in the model are organized as an *I*-by-*K* table, *X*. All features are mean-centered and standardized. Any quadratic terms are created using the standardized features to reduce the dependence structure of the target posterior distribution.

Because not every linear feature will be relevant to each of the three predictions (downregulated, unchanged and upregulated), we divide the linear features *X* into three (potentially overlapping) subsets, denoted *X*^(−)^, *X*^(0)^ and *X*^(+)^. For example, the read count of an exon influences the statistical power to detect changes in splicing and, hence, should inform the predicted probability of being unchanged, *P*^(0)^ and is, thus, assigned to *X*^(0)^. The original inclusion level of an exon determines whether upregulation or downregulation is detectable because there is little room for inclusion to increase or decrease when the exon was already nearly 100% or 0% included. Thus, the inclusion level before SF perturbation is likely directly informative of ${p}_{i}^{(-)}$ and ${p}_{i}^{(+)}$ is, therefore, assigned to both *X*^(−)^ and *X*^(+)^. These features are incorporated into the predictions as

$$\begin{array}{c}{q}_{i}^{(-)}=-\mathop{\sum }\limits_{j=1}^{J}B\left({S}_{ij};\lambda ,m,d\right){\alpha }_{j}+\mathop{\sum }\limits_{k=1}^{{K}^{(-)}}{\beta }_{k}^{(-)}{X}_{ik}^{(-)}+{\beta }_{0}^{(-)}\\ {q}_{i}^{(0)}=\mathop{\sum }\limits_{k=1}^{{K}^{(0)}}{\beta }_{k}^{(0)}{X}_{ik}^{(0)}\\ {q}_{i}^{(+)}=\mathop{\sum }\limits_{j=1}^{J}B\left({S}_{ij};\lambda ,m,d\right){\alpha }_{j}+\mathop{\sum }\limits_{k=1}^{{K}^{(+)}}{\beta }_{k}^{(+)}{X}_{ik}^{(+)}+{\beta }_{0}^{(+)}\end{array},$$

where ${\beta }_{1\ldots {K}^{(-)}}^{(-)}$, ${\beta }_{1\ldots {K}^{(0)}}^{(0)}$ and ${\beta }_{1\ldots {K}^{(+)}}^{(+)}$ denote the linear coefficients describing the effect of the linear features on the predictions and where *β*^(−)^ and *β*^(+)^ are the intercepts.

#### Connecting predictions to observed splicing changes

The probabilities for category *z* are computed as

$${\hat{p}}_{i}^{(z)}=\frac{\exp \left({q}_{i}^{(z)}\right)}{\exp \left({q}_{i}^{(-)}\right)+\exp \left({q}_{i}^{\left(0\right.}\right)+\exp \left({q}_{i}^{(+)}\right)}.$$

We connect these predictions to the observed splicing changes with a categorical distribution:

$${Y}_{i} \sim {\rm{Categorical}}\left(p=\left[{\hat{p}}_{i}^{(-)},{\hat{p}}_{i}^{(0)},{\hat{p}}_{i}^{(+)}\right]\right).$$

The probability of an observation under this distribution is simply equal to the predicted probability for that category. For example, if exon *i* is downregulated, the likelihood is $P({Y}_{i}| {\hat{p}}_{i})={\hat{p}}_{i}^{(-)}$.

### Defining the model’s priors

We represent assumptions about our model’s parameters with Bayesian priors. Some of the priors we use reflect what we consider plausible given our understanding of molecular biology. Others reflect statistical considerations that aim to avoid unrealistically strong predictions or complications to statistical inference. For example, given the limitations of experimental data and modeling thereof, we do not believe KATMAP should ever make extremely confident predictions on the order of million-to-one odds. In other cases, we wish to avoid identifiability issues, where many combinations of distinct parameter values would yield essentially the same predictions. In describing the model’s priors, we note for each parameter which consideration is involved.

#### Priors on the binding parameters

The parameter *λ* scales the motif scores *S* to assign binding sites log affinities *S* × *λ*. When *λ* < 1, the scores are compressed, assigning a narrower range of affinities. When *λ* > 1 the scores are stretched, assigning a wider range of affinities. We choose a prior that asserts both to be equally plausible. Beyond this, the prior we place on *λ* largely reflects a practical consideration about operations on a log scale. Multiplication on the log scale corresponds to exponentiating the actual affinities:

$${\rm{Affinity}}={e}^{\;\lambda S}={{e}^{S}}^{\lambda }.$$

**Extended Data Fig. 9:**
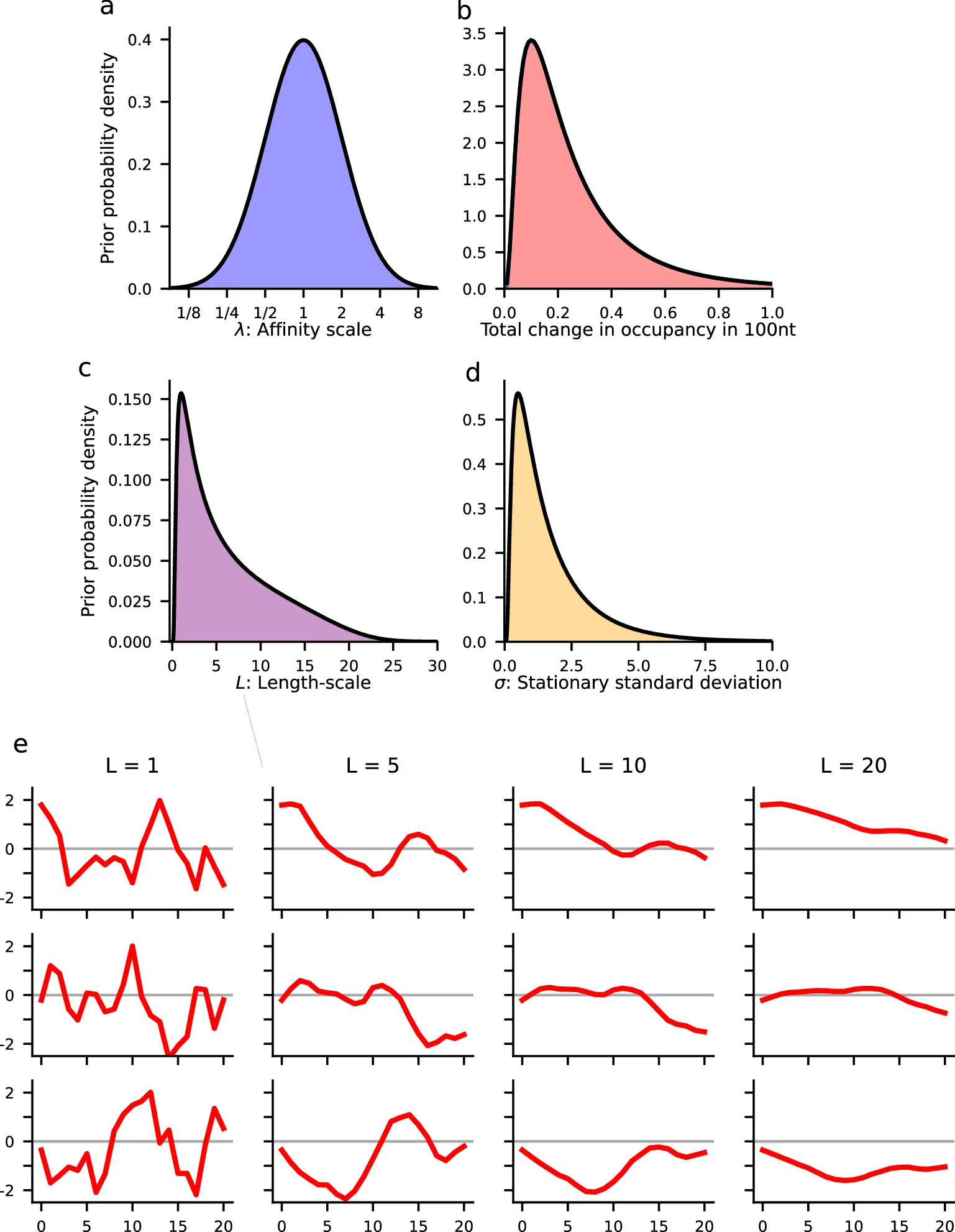
Illustrations of the priors on the hyperparameters and resulting spatial correlations. Prior densities on **a**) the affinity scale (in ${\log }_{2}$-scale); **b**) the average amount of binding lost per exon (implicit prior on most-relevant-affinity); **c**) the length-scale of the Gaussian process prior (in units of 10 nt regions) on the activity coefficients; and **d**) stationary standard deviation of the Gaussian process. **e**) Examples of the spatial smoothness resulting from different length-scales.

Even modest values of *λ* will yield extremely large or small affinities. Thus, we constrain *λ* to a relatively narrow range of values by using a unit-normal prior:

$${\log }_{2}\lambda \sim \rm{Normal}(0,1),$$

which places about 99% of the prior probability on *λ* between $\frac{1}{6}$ and 6 (Extended Data Fig. 9a).

The most relevant affinity parameter *m* describes which affinity experiences the greatest change in binding. Assuming that the binding site model is accurate, motifs with moderate to high affinity should experience changes in binding; motifs with very low affinity are too weak to be appreciably bound in the first place. Thus, a biologically reasonable prior would favor values of *m* closer to the affinity of the optimal motif and disfavor very low affinities. Beyond this, it is difficult to precisely express how plausible a given value of *m* is.

However, *m* determines how much SF occupancy our model predicts was lost from near each exon. It is easier to assess the biological plausibility of these statements. Certain values of *m* might imply that the average exon has >20 copies of the SF bound within a few hundred nucleotides. For SFs that bind specific sequence motifs, this is unrealistically high. Thus, rather than explicitly define a prior on *m*, we instead define our prior on the average change in binding near each exon:

$$\bar{B}=\left\vert \frac{1}{N}\mathop{\sum }\limits_{i=1}^{N}\mathop{\sum }\limits_{j=1}^{J}B({S}_{ij};m,\lambda ,d)\right\vert.$$

We divide this quantity by the number of potential binding sites per exon (*J* = 52 in our analyses) and place the prior on the log odds of this fraction:

$$\tilde{B}={\frac{\bar{B}+\epsilon }{J+2\epsilon }},$$

with small constants, *ϵ* = 10^−7^, added to the numerator and denominator to avoid numerical issues because of computational precision. We then define our prior as

$$\log {\frac{\tilde{B}}{1-\tilde{B}}} \sim {\mathrm{Normal}}(\mu =-3.9,\sigma =0.84),$$

which places 99% of the prior probability on the average change in binding per exon between 0.12 and 7.8 (Extended Data Fig. 9b). To exclude extremely unrealistic values of *m*, we constrain the model to only consider values between the median log affinity and the maximum affinity per 10-nt region, log(10) (or, more generally, $\log ({\rm{region}}\;{\rm{size}})$).

#### Assuming that activity varies smoothly with distance

A fundamental assumption of our model is that the effect of SF binding on exon inclusion depends on where the protein is bound relative to the exon. This is described by activity coefficients *α*_1_, …, *α*_*J*_. We further assume that the activities of nearby regions, say *j* = 2 and *j* = 3, are similar but that distant positions may have very different activities. Statistically, we express this with a Gaussian process prior that assumes that the activity coefficients are spatially correlated^65^. The Gaussian process can be conceptualized as a multivariate normal (${\mathcal{N}}$) distribution, where the covariance between variables *α*_*j*_ and *α*_*k*_ is a decreasing function of how far apart they are ∣*j* − *k*∣.

$${\alpha }_{1\ldots J} \sim {\mathcal{N}}\left(0,\Sigma (L,\sigma )\right)$$

We choose the smooth yet flexible Matern-3/2 covariance function to express these spatial correlations^65^. Because we further want to assume that the effects of exonic versus intronic binding may be very different, we restrict the spatial correlations to pairs of regions that fall on the same side of a given SS. We express this with a set of labels *R*_1_, …, *R*_*J*_ that indicate whether region *j* is on the intronic side of the 3SS or the exonic side, or is on the exonic side of the 5SS or the intronic side. If a pair of regions *j* and *k* correspond to different biological labels, the spatial correlation between *α*_*j*_ and *α*_*k*_ is set to zero:

$${\Sigma }_{jk}(L,\sigma )=\left\{\begin{array}{ll}{\sigma }^{2}\left(1+\frac{\sqrt{3}| j-k| }{L}\right)\exp \left(-\frac{\sqrt{3}| j-k| }{L}\right)\quad &{\rm{if}}\,{R}_{j}={R}_{k}\\ 0\quad &{\rm{if}}\,{R}_{j}\ne {R}_{k}.\end{array}\right.$$

Assuming this spatial correlation structure requires introducing two additional parameters that must be learned. The length scale, *L*, determines how far apart a pair of regions *j* and *k* must be before they are weakly correlated. The stationary s.d., *σ*, determines the range of magnitudes the activity coefficients are expected to span. To prevent the inference algorithm from wasting computation by exploring large but practically equivalent regions of parameter space, we constructed priors that avoid both large and small values of these hyperparameters (Extended Data Fig. 9c,d; mathematical details in Supplementary Information). This places 99% of the prior probability for the length scale on the interval 3.2 nt < *L* < 226 nt (Extended Data Fig. 9c), which ranges from near independence to correlations that span the whole intronic window (Extended Data Fig. 9e). For the stationary s.d., 99% of the prior probability falls in the interval 0.14 < *σ* < 8.7 (Extended Data Fig. 9d).

#### Assumptions about the linear coefficients

Because we mean-center and standardize the linear features, *β*_*k*_ indicates how much an increase in linear feature *k* by one s.d. changes the log odds of the prediction. Because the impact is on a log odds scale, coefficients of magnitude ∣*β*∣ > 10 would have extreme impacts on the prediction; increasing the linear feature by one s.d. would increase a predicted probability of *P* = 0.01 to *P* > 0.99. As the standardized features typically span a range of more than four s.d., we used a weakly informative prior for the linear coefficients and intercept, using a normal density with mean of 0 and s.d. of 5:

$${\beta }_{0\ldots K} \sim {\rm{Normal}}(\mu =0,\sigma =5).$$

The model is not sensitive to this choice of prior, as it spans the range of reasonable effect sizes, both strong and weak.

### High-level overview of model inference

The goal of Bayesian inference is to determine which parameters are plausible given the observed data. In practice, this is often a harder task than formulating the statistical model itself. Evaluating the exact plausibility requires applying Bayes’ theorem, which expresses a reasonable proposition: to determine how plausible a given hypothesis *h* is, you must compare it to every other hypothesis admitted by the model, $\tilde{h}\in \Theta$:

$$P(h| Y)=\frac{P(Y| h)P(h)}{{\int}_{\tilde{h}\in \Theta }P(Y| \tilde{h})P(\tilde{h})d\tilde{h}}.$$

This is typically impossible to compute exactly because it requires integrating over the entire parameter space. In the case of KATMAP’s regression model, this parameter space has >50 dimensions.

A common solution is Monte Carlo sampling. Rather than evaluate the exact posterior density *P*(*θ*, *ϕ*∣*Y*), we instead seek to obtain a sample of hundreds of parameter values that follow the posterior distribution:

$$({\theta }_{1},{\phi }_{1}),\ldots ,({\theta }_{N},{\phi }_{N}) \sim P(\theta ,\phi | Y).$$

This posterior sample provides a representative set of parameters that are plausible given the observed data from which we can estimate the posterior mean. We can also construct CIs describing the range of plausible parameters values. Because the model predictions for each exon are computed on the basis of these parameters, it is further possible to obtain interval estimates for all of the exon-level predictions.

### Bayesian inference with the KATMAP model

When writing the posterior for KATMAP’s model, it is useful to divide the model parameters into two groups. The first group we term hyperparameters *φ* = (*λ*, *m*, *L*, *σ*) because, if these four parameters are held fixed, the model reduces to a much simpler generalized linear model (GLM). The second group represents the regression coefficients *θ* = (*α*_1_, …, *α*_*J*_ and β_0_, …, β_*K*_) of this conditional GLM. Partitioning the parameters in this manner makes clear the hierarchical nature of the model:

$$P(\theta ,\varphi | Y)\propto P(Y| \theta ,\varphi )P(\theta | \varphi )P(\varphi ).$$

INLA provides a means to leverage this structure and divide the problem of posterior inference into two manageable, nested subproblems^23^. First, an inner step focuses on identifying which regression coefficients are plausible given the data and some specified values of hyperparameters. This approximates the conditional posterior *P*(*θ*∣*Y*, *φ*) for a specific hyperparameter value, *φ*. Second, an outer step focuses on determining which hyperparameters are plausible given the data. This approximates the marginal posterior for the hyperparameters *P*(*φ*∣*Y*).

#### Approximating the conditional posterior over the regression coefficients

The inner step takes advantage of how the model simplifies if the hyperparameters are held fixed to a particular value. The known binding parameters (*λ*, *m*) would transform the table of motif scores *S* into a fixed table of changes in binding *B*_(*λ*, *m*)_. The known spatial correlation parameters would yield a fixed prior on the activity coefficients. The problem reduces to a GLM, albeit a Bayesian formulation with priors.

We use second-order optimization identify the optimal regression coefficients as the mode of the conditional posterior, ${\theta }_{\varphi }^{* }$, determining the step size with a backtracking line search^66^. This requires evaluating the gradient ∇ and Hessian matrix *H*, which we compute using automatic differentiation with the JAX library (http://github.com/google/jax). How sharply the log posterior is curved around the mode is described by the Hessian matrix ${H}_{{\theta }^{* }}$ and can be used to describe the uncertainty about the coefficients with a covariance matrix, ${\Sigma }_{\varphi }^{* }={H}_{{\theta }_{\varphi }^{* }}^{-1}$. This provides a multivariate normal approximation to the conditional posterior:

$$P(\theta | Y,\varphi )\approx {\mathcal{N}}(\theta ;\mu ={\theta }_{\varphi }^{* },\Sigma ={\Sigma }_{\varphi }^{* }).$$

#### Approximating the marginal posterior over the hyperparameters

The outer step evaluates the plausibility of specific hyperparameters *φ* by approximating the marginal posterior, *P*(*φ*∣*Y*). The hyperparameters, *φ* only relate to the observations through the regression coefficients *θ*. Hence, evaluating the plausibility of some specific *φ* requires considering all possible values the regression coefficients *θ* could take. Rue et al.^23^ accomplish this by using the normal approximation to the conditional posterior obtained in the inner step evaluated at its mode, ${\theta }_{\varphi }^{* }$

$$\begin{array}{rcl}P(\varphi | Y)\propto \sim \frac{P(Y,{\theta }_{\varphi }^{* },\varphi )}{{\mathcal{N}}({\theta }_{\varphi }^{* };{\theta }_{\varphi }^{* },{\Sigma }_{\varphi }^{* })}&=&P(Y,{\theta }_{\varphi }^{* },\varphi )\det {\left({(2\pi )}^{d}\left.{\Sigma }_{\varphi }^{* }\right)\right)}^{\frac{1}{2}}\\ &=&P(Y,{\theta }_{\varphi }^{* },\varphi ){\int}_{x\in {{\mathbb{R}}}^{d}}\exp\\&&\left({\left.{\theta }_{\varphi }^{* }-x\right)}^{T}{{\Sigma }_{\varphi }^{* }}^{-1}({\theta }_{\varphi }^{* }-x)\right)dx\end{array}$$

This approximation to the marginal density multiplies the joint density $P(Y,{\theta }_{\varphi }^{* },\varphi )$ by the volume of regression coefficients that are plausible given *φ*.

#### Monte Carlo sampling from the joint posterior with INLA

**Extended Data Fig. 10:**
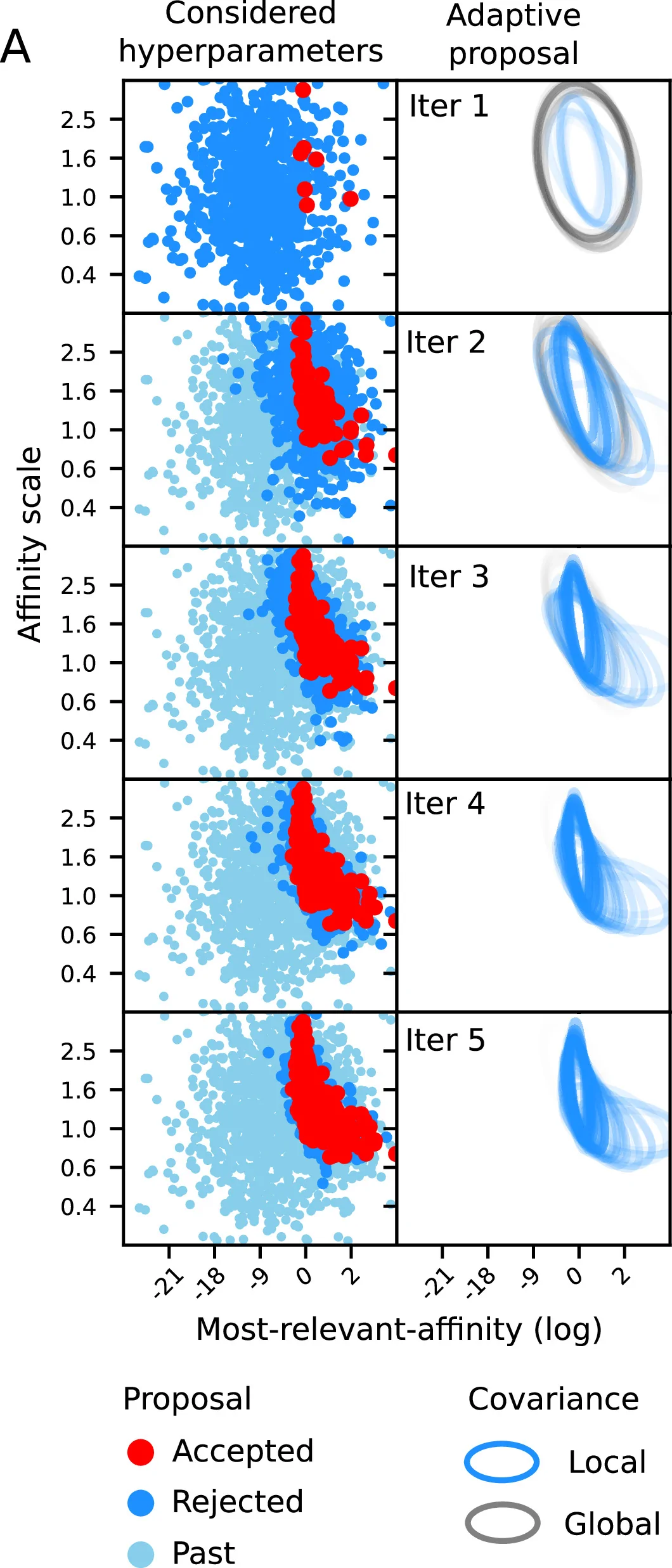
Illustration of posterior inference with for the KATMAP model. **a**) Exploration of the hyperparameters through adaptive importance sampling, showing the evaluation points (left) and the adaptive proposal distribution at successive iterations. New hyperparameters (blue) are drawn from the proposal estimated in the previous iteration, which are in turn used to refine the adaptive proposal distribution to better match the target posterior. The proposal is a student T mixture model fit by importance-weighted Expectation-Maximization. Upon termination, hyperparameters are accepted by importance sampling to obtain a sample from the posterior (blue).

We couple adaptive importance sampling with these INLAs to draw samples of plausible parameters from the joint posterior. First, we use adaptive importance sampling and the INLA approximation to draw a representative sample of plausible hyperparameters from their marginal posterior, *φ*_1_, …, *φ*_*N*_ ~ *φ*∣*Y*. This explores the hyperparameters in a way that iteratively hones in on the true posterior by fitting mixture model approximations of the marginal posterior with importance-weighted expectation maximization^22^ (Extended Data Fig. 10a; mathematical details in Supplementary Information). Second, for each sampled hyperparameter, we solve the resulting conditional model by optimization, approximating the conditional posteriors (including their correlation structure) over the regression coefficients with a multivariate Gaussian. We then sample regression coefficients from these conditional posteriors, *θ*_1_, …, *θ*_*N*_ ~ *θ*∣*Y*, *φ*_1, …, *N*_. Together, these constitute an approximate sample from the joint posterior, (*θ*_1_, *φ*_1_), …, (*θ*_*N*_, *φ*_*N*_) ~ *θ*, *φ*∣*Y*. We run this algorithm twice for each knockdown experiment, generating two separate posterior samples. If both runs converge to the same distribution, we consider inference successful and merge the samples.

#### Evaluating convergence

We use two criteria to assess whether our Monte Carlo samples are reliable representations of the target posterior. First, we use the split $\hat{r}$-statistic^67^ to assess whether our two inference runs converged to the same distribution. Values of $\hat{r}$ close to 1 indicate highly overlapping distributions and if marginal distributions of all parameters yield $\hat{r} < 1.05$, we consider the Monte Carlo sampling runs to have converged. Second, during adaptive importance sampling of the hyperparameters, we stabilize the importance weights with Pareto smoothing. This automatically provides a diagnostic statistic, $\hat{k}$, indicating whether the resulting importance sample is reliable. We use the cutoff suggested by Vehtari et al.^68^, requiring $\hat{k} < 1-\frac{1}{lo{g}_{10}{N}_{\rm{proposals}}}$ or $\hat{k} < 0.7$ (whichever is more stringent) for the posterior samples to be considered trustworthy.

#### Evaluating evidence of splice regulatory activity

To quantify evidence of in favor of splice regulatory activity, we compare to a reduced model with the splicing impacts removed. We compare the full and reduced models using Pareto-smoothed importance sampling leave-one-out cross-validation^68^, a fully Bayesian approach to model comparison that uses the posterior samples from the full and reduced models to estimate whether the full model better predicts held-out data. We bootstrap observations to compute a *z* score describing our confidence that including splicing activity in the model improves performance.

#### Determining which activity maps are significant

We consider an activity map significant on the basis of two criteria. First, we require that at least two activity coefficients be significantly nonzero (98% CI does not overlap zero). Second, we require a model comparison *z* score > 2.

We additionally considered whether the splicing changes are well described by the expected motif. We excluded any activity models that chose binding parameters that assigned an unreasonable number of binding changes per exon (>20). This indicates that the knockdown-affected exons are distinguished by some characteristic sequence composition but it is not well described by the perturbed factor’s expected motif. This excluded SRSF9 (HepG2) from our analysis, which yielded a significant map by assigning appreciable occupancy to most sites in the transcriptome.

#### Clustering activity maps for visualization

When clustering the activity maps from different knockdowns ${\hat{\alpha }}^{(1)},{\hat{\alpha }}^{(2)},\ldots ,{\hat{\alpha }}^{(N)},$ we wanted to account for the uncertainty in the posterior samples. Each inferred activity map is a posterior sample with a value ${\hat{\alpha }}_{i,j}^{(f)}$ at each position *j* for each *M*^(*f*)^ from the posterior. We scale each sample *i* from the posterior by the absolute value of the largest activity coefficient in that sample and compute the posterior mean and s.d. at each position *j* for the activity map of each knockdown *f* as

$${\mu }_{f,j}=\frac{1}{M}\mathop{\sum }\limits_{i=1}^{M}\frac{{\hat{\alpha }}_{i,j}^{(f)}}{\max {\hat{\alpha }}_{i,\cdot}^{(f)}}$$

and

$${\sigma }_{f,j}=\sqrt{\frac{1}{M}\mathop{\sum }\limits_{i=1}^{M}{\left(\frac{{\hat{\alpha }}_{i,j}^{(f)}}{\max {\hat{\alpha }}_{i,\cdot}^{(f)}}-{\mu }_{f,j}\right)}^{2}}.$$

To compute the difference between a pair of activity maps ${\hat{\alpha }}^{(x)}$ and ${\hat{\alpha }}^{(y)}$ we compute the absolute difference between the posterior means ∣*μ*_*x*,*j*_ − *μ*_*y*,*j*_∣ and normalize this by the expected s.d. of the difference $\sqrt{{\sigma }_{x,j}^{2}+{\sigma }_{y,j}^{2}}$, averaging this *z* score across all positions 1, .., *J:*

$${\rm{Distance}}_{xy}=\frac{1}{J}\mathop{\sum }\limits_{j=1}^{J}\frac{| {\mu }_{x,j}-{\mu }_{y,j}| }{\sqrt{{\sigma }_{x,j}^{2}+{\sigma }_{y,j}^{2}}}.$$

We use hierarchical clustering using this metric with average linkage and use optimal leaf sorting to order the dendrogram.

### Extending the additive models to alternative splicing events

While the outlined additive model works well for cassette exons, there may be cases where more complicated but related models may be desired. For example, alternative SS choice can be influenced by whether an exonic enhancer or silencer is located between the two alternative SSs. We structured KATMAP with enough flexibility to accommodate these cases; however, for the sake of simplicity, they are not the primary focus of this work. When computing the splicing impact for each exon *i*, we sum the positional impacts over different regions *j* in windows centered around the exon’s SSs.

$$\Delta A_{i}={\mathop{\sum }\limits_{j=1}^{J}}B({{{S}}}_{ij};\lambda ,m,d){\alpha }_{j}$$

We extend our model by truncating these windows of sequence when they extend past another SS associated with exon *i*. To do this, we introduce a precomputed set of weights, *τ*_*ij*_, equal to the fraction of coordinates within region *j* where the corresponding window does not extend past another SS associated with exon *i*. If region *j* does not extend past another SS, *τ*_*ij*_ = 1; if region *j* overlaps another SS *τ*_*ij*_ takes a fractional value; if region *j* falls on the other side of another SS, *τ*_*ij*_ = 0. These are incorporated into the splicing impacts as

$$\Delta {{{A}}}_{i}={\mathop{\sum }\limits_{j=1}^{J}}B({{{S}}}_{ij};\lambda ,m,d){\tau }_{ij}{\alpha }_{j}.$$

This can be be turned on in the command line interface by setting ‘--annotation-based-activity’ to ‘True’.

When learning models from alternative 3SS and 5SS splicing events, we reduced the intronic and exonic window sizes to 100 and 60 nt, respectively, and only considered knockdowns with at least 50 significant splicing changes.

### Evaluating a reduced model without splicing activity

To evaluate evidence of splice regulatory activity, we compare to a reduced version of the model that only includes the linear predictors. The predictions under this model are computed as

$$\begin{array}{c}{q}_{i}^{{{\emptyset}}(-)}=\mathop{\sum }\limits_{k=1}^{{K}^{(-)}}{\beta }_{k}^{(-)}{X}_{ik}^{(-)}+{\beta }_{0}^{(-)}\\ {q}_{i}^{{{\emptyset}}(0)}=\mathop{\sum }\limits_{k=1}^{{K}^{(0)}}{\beta }_{k}^{(0)}{X}_{ik}^{(0)}\\ {q}_{i}^{{{\emptyset}}(+)}=\mathop{\sum }\limits_{k=1}^{{K}^{(+)}}{\beta }_{k}^{(+)}{X}_{ik}^{(+)}+{\beta }_{0}^{(+)}\end{array}.$$

This is a standard multinomial logistic regression and has no hyperparameters that would necessitate the use of INLA. We approximate the posterior distribution over the regression coefficients using the same optimization and Laplace approximations used to evaluate the conditional posterior for the full model. We then draw a posterior sample from the reduced model, ${\theta }_{1\ldots N}^{({{\emptyset}})}$, of the same size as the posterior sample obtained for the full model.

### Predicting direct targets

We consider an exon a direct target if its splicing impact is sufficiently large to improve upon a reduced model without splicing activity. To assess this, we compare the posterior distribution of predictions between the full and the reduced models, *p* and ${p}^{{{\emptyset}}}$. In a knockdown experiment, enhancing targets should be downregulated and suppression targets upregulated. Hence, we consider an exon an enhancing target if we are confident the splicing impact, *A*_*i*_, is negative enough to improve the downregulation prediction,

$$P({A}_{i} < 0| Y) > 0.9\quad {\rm{and}}\quad P\left({p}_{i}^{(-)} > \left.{p}_{i}^{{{\emptyset}}(-)}\right\vert Y\right) > 0.9,$$

and a suppression target if the splicing impact is positive enough to improve the upregulation prediction,

$$P({A}_{i} > 0| Y) > 0.9\quad {\rm{and}}\quad P\left({p}_{i}^{(+)} > \left.{p}_{i}^{{{\emptyset}}(+)}\right\vert Y\right) > 0.9.$$

This assigns each exon into one of three mutually exclusive categories: enhancing target, suppression target and nontarget. In some analyses, we wish to evaluate the confidence of these classifications and we do so with a multinomial regression that asks what fraction of exons with a given splicing impact were assigned to each category. This assigns each exon a probability of being an enhancing target, a nontarget or a suppression target on the basis of its splicing impact. We assumed that fractions of direct targets differ among upregulated, downregulated and nonsignificant exons by fitting separate intercepts for each category.

### Evaluating eCLIP enrichment at targets

For SFs with significant activity maps and available eCLIP data, we downloaded the BED files representing peak calls for both replicates. We did not use the results of the irreproducible discovery rate analysis because we believe this to represent an overly stringent criteria for combining replicate information. We considered the presence of a peak >2-fold enriched over input in at least one of the eCLIP replicates as evidence of potential binding. The signal of differential splicing and eCLIP binding are clearest in highly expressed transcripts because of better representation in the sequencing reads; evaluating enrichment between differentially spliced and nonsignificant exons risks spurious associations because of correlated statistical power. To avoid this, we compared binding at targets to nontargets that significantly changed in the same direction. We further focused on the positions predicted by KATMAP to impact splicing. For both targets and nontargets, we counted the number of exons with evidence of binding, *k*_target_ and *k*_nontarget_, and used a beta-binomial conjugate model with a uniform prior on the fractions (*α*_prior_, *β*_prior_ = 1) to obtain 2,000 posterior samples of the fractions of bound target and nontarget exons, *f*_target_ and *f*_nontarget_:

$$\begin{array}{c}{f}_{\rm{target}}| {k}_{\rm{target}} \sim \rm{Beta}(\alpha ={k}_{\rm{target}}+1,\beta ={N}_{\rm{target}}-{k}_{\rm{target}}+1)\\ {f}_{\rm{nontarget}}| {k}_{\rm{nontarget}} \sim \rm{Beta}(\alpha ={k}_{\rm{nontarget}}+1,\beta ={N}_{\rm{nontarget}}-{k}_{\rm{nontarget}}+1)\end{array}$$

where *N*_target_ and *N*_nontarget_ are the total number of predicted targets and nontargets that significantly changed in a given direction. We constructed a posterior sample of enrichment estimates as

$${\log }_{2}\frac{{f}_{\rm{target}}}{{f}_{\rm{nontarget}}}$$

and computed the means and CIs from this.

### Defining effect sizes for changes in splicing

Differential splicing analyses typically report inclusion changes as *Δψ* = *ψ*_*kd*_ − *ψ*_*ctrl*_. However, under the hood, the statistical analysis is typically performed with respect to the log odds transformation of inclusion, $\phi ={\log }_{2}\frac{\psi }{1-\psi }$, with the effect size being the log odds ratio of inclusion before and after knockdown.

$$\Delta \phi ={\phi }_{kd}-{\phi }_{ctrl}={\log }_{2}\frac{{\psi }_{kd}/\left(1-{\psi }_{kd}\right)}{{\psi }_{ctrl}/\left(1-{\psi }_{ctrl}\right)}$$

While our model does not aim to predict the magnitude of inclusion changes, only the direction, our analyses make use of inclusion levels in two ways. First, we use the pre-knockdown inclusion level, *ϕ*_*ctrl*_ as a linear predictor, being informative of whether an increase or decrease in inclusion is detectable. Second, we assess whether our splicing impacts can predict the magnitudes of inclusion changes, despite not being trained on them. In both cases, we favor the log odds representation.

The log odds representations, *ϕ* and *Δϕ*, offer two advantages. First, *ψ* by definition must be between 0 and 1; thus, the magnitude of *Δψ* only has meaning in the context of the original inclusion level. Here, *Δψ* = +0.1 is valid for *ψ*_*ctrl*_ = 0.05 but not for *ψ*_*ctrl*_ = 0.95 because *ψ*_*kd*_ = 1.05 is impossible. A second, related concern, is that inclusion levels near 0 or 1 likely represent more extreme splicing contexts than does intermediate inclusion. A change in inclusion from 0.5 to 0.6 corresponds to *Δψ* = 0.1, as does a change from 0.01 to 0.11. However, an exon included in only 1% of transcripts likely represents a strong silencing context or has weak SSs; hence, the increase of 10% likely reflects a more substantial change in regulation than a change from 50% to 60%. The odds ratio reflects this, treating an inclusion change of 0.5 to 0.6 as representing a smaller effect (*Δϕ* ≈ +0.6) than a change of 0.01 to 0.11 (*Δϕ* ≈ +3.6).

When comparing the effect sizes between predicted targets and nontargets, we only compared exons that were differentially spliced in the same direction. For ‘nonsignificant’ exons, we computed the average effect size in two ways. First, we simply averaged across all nonsignificant exons. However, these likely include constitutively included or excluded exons with very strong or weak splicing contexts. To focus on exons could be subject alternative splicing regulation by SFs, we also computed the average effect size excluding exons with confidently small *Δψ* values. We computed CIs for these point estimates using Bayesian bootstrapping.

### GO analysis of QKI–RBFOX cooperatively regulated exons

We defined confident QKI–RBFOX cotargets as predicted targets of either QKI or RBFOX that were downregulated upon knockdown and were inferred to also be regulated by the other factor based on splicing impacts. For the latter criteria, we chose a splicing impact cutoff of <−0.25 by examining the empirical cumulative distribution functions (eCDFs) (Fig. 5c,d). We constructed expression-matched background sets from all genes expressed in the QKI and RBFOX HepG2 knockdowns using importance sampling and used GOrilla to identify enriched Gene Ontology (GO) terms relative to this background^69^. We also performed this analysis using all differentially spliced QKI and RBFOX targets as the background. We used all three ontologies: process, function and component. We identified significantly enriched terms using a 10% FDR threshold.

### Inferring SF perturbations from splicing changes

To explain a set of observed splicing changes in terms of changes in SF regulation, we use the splicing impacts previously inferred from the knockdown data as the linear features in a multinomial regression. For each SF, *f*, with a significant activity map, we use the posterior means of its activity (${\hat{\alpha }}_{1, \ldots , J}^{(f)}$) and binding parameters (${\hat{\lambda }}^{(f)}$, ${\hat{m}}^{(f)}$) to compute splicing impacts for every considered exon *i*:

$${{{A}}}_{i}^{(f)}={\mathop{\sum }\limits_{j=1}^{J}}B\left({{{S}}}_{ij};{\hat{\lambda }}^{(f)},{\hat{m}}^{(f)},-{\frac{1}{2}}\right){\hat{\alpha }}_{j}.$$

We incorporate these into a regression model, including linear coefficients to account for unperturbed inclusion levels, expression and their squares:

$$\begin{array}{c}{q}_{i}^{(-)}=-\mathop{\sum }\limits_{f\in SF}{\gamma }^{(f)}{{{A}}}_{i}^{(f)}+\mathop{\sum }\limits_{k=1}^{{K}^{(-)}}{\beta }_{k}^{(-)}{X}_{ik}^{(-)}+{\beta }_{0}^{(-)}\\ {q}_{i}^{(0)}=\mathop{\sum }\limits_{k=1}^{{K}^{(0)}}{\beta }_{k}^{(0)}{X}_{ik}^{(0)}\\ {q}_{i}^{(+)}=\mathop{\sum }\limits_{f\in SF}{\gamma }^{(f)}{{{A}}}_{i}^{(f)}+\mathop{\sum }\limits_{k=1}^{{K}^{(+)}}{\beta }_{k}^{(+)}{X}_{ik}^{(+)}+{\beta }_{0}^{(+)}\end{array},$$

where each *γ*^(*f*)^ is a coefficient describing how predictive the splicing impacts for SF *f* are of the splicing changes. We describe the observed splicing changes *Y*_1, …, *N*_ as following a categorical distribution:

$$\begin{array}{rcl}{\hat{p}}_{i}^{(z)}&=&\frac{\exp \left({q}_{i}^{(z)}\right)}{\exp \left({q}_{i}^{(-)}\right)+\exp \left({q}_{i}^{\left(0\right.}\right)+\exp \left({q}_{i}^{(+)}\right)}\\ {Y}_{i}& \sim &{\rm{Categorical}}\left(p=\left[{\hat{p}}_{i}^{(-)},{\hat{p}}_{i}^{(0)},{\hat{p}}_{i}^{(+)}\right]\right)\end{array}$$

Because all of the splicing impacts A are precomputed, this is a standard multinomial logistic regression. We use second-order optimization to find the posterior mode and, as described previously, use the curvature at the mode to approximate the posterior with a multivariate Gaussian, obtaining the posterior mean and s.d. for each coefficient. When considering perturbations inferred from many knockdown datasets, we use ashr to apply shrinkage to the estimates and compute FDR-corrected *P* values. These shrinkage estimates reflect the assumption that only a subset of SFs were truly perturbed (that is, sparsity). We use the half-normal mixture option to reflect the possibility that the proportions of gains and losses in regulation are unequal.

We compared this to a simpler approach instead aiming to infer perturbations from inclusion changes rather than splicing impacts. Here, we set *A* equal to the ashr shrinkage estimate for dPSI observed in the knockdown dataset the splicing impacts would have been learned from. We standardize but do not mean-center the inclusion changes to allow the scale of the coefficients to be compared across factors, while preserving the sign of the inclusion changes. If an exon *i* was not observed in knockdown *f*, we set ${{{A}}}_{i}^{(f)}$ equal to zero such that factor *f* simply does not contribute to the prediction at exon *i*.

In our analyses of the ENCODE data, we use the same training data to define the observations and linear predictors used to learn the KATMAP models. When applying this approach the PDA cancer data, we used previous data (Supplementary Table 2 from another study^62^) to define the training data and sought to match the criteria used in the original analysis to define significant exons. We defined inclusion changes as metastatic versus primary, restricted our analysis to cassette exons observed in at least 75% of samples and defined exons as significantly upregulated or downregulated if inclusion changed by at least ±10% and the reported FDR-corrected *P* value was less than 0.05. We included all significant exons and 5,000 randomly selected nonsignificant exons in the training set. Because raw reads counts were not reported, we could not include log read counts as a linear feature but included the average inclusion level (in log odds scale) from the primary tumor samples as a linear and quadratic predictor.

### Comparing inferred perturbations to MAPP

To compare our perturbation analyses to MAPP, we downloaded the MAPP results from the ENCODE knockdowns from Zenodo^70^. For each knockdown, we extracted the 3SS and 5SS absolute *z* scores MAPP assigned to each PWM, selecting the maximum of the two splicing *z* scores. Typically MAPP aims to distinguish between a large library of motifs, including multiple motifs per factor and paralogs. To make the comparison fairer to MAPP, when computing the rank of the *z* scores, we only required MAPP to distinguish between SFs also considered by KATMAP. If an SF had multiple motifs in the MAPP outputs, we selected the motif with the highest *z* score. If an SF had paralogs (for example, PCBP1 and PCBP2), we selected the paralog with the highest *z* score, considering MAPP to have identified the correct factor if it chose a paralog of the knocked down factor. Because KATMAP models are learned from knockdown data, we limited this analysis to SFs with knockdowns in both cell types and, for the knocked down SF, always used the model learned from the other cell type to infer perturbations (to avoid circularity). We considered SFs for which we had KATMAP models and which had PWMs in the MAPP library.

### Simulation study of model inference

For our simulation studies, we wished to assess how reliably our inference algorithm could recover the parameters that truly generated the observed splicing changes. For our true parameters, (*θ*, *φ*)^(true)^, we used the posterior means inferred from twenty-one knockdowns that yielded significant activity maps. These parameters coupled with the predictor variables (*S* and *X*) naturally assign each exon probabilities of being downregulated, unchanged or upregulated after knockdown. So that our simulated data provide enough statistical power to make confident inferences, we adjust the intercepts to ensure around 500 upregulated and downregulated exons. To generate the simulated observations *Y*^(*sim*)^, we sample an outcome for each exon from a categorical distribution based on these predicted probabilities. We then use KATMAP to predict these simulated observations using the original predictor variables *S* and *X* to obtain a posterior sample (*θ*, *φ*)_1_, …, (*θ*, *φ*)_*N*_∣*Y*^(sim)^. We computed posterior means and CIs and compared these to the true values, (*θ*, *φ*)^(true)^.

#### Statistical analyses

We represent uncertainty in our analyses with CIs. For KATMAP’s inferences, these are obtained by Monte Carlo sampling. With estimates based on count or presence and absence data, we use the beta-binomial conjugate model, with uniform priors. For frequency estimates so obtained, we compute the mean and CI from the posterior beta distribution. For enrichment estimates, we sample frequencies from the posteriors of foreground and background to obtain a posterior sample of enrichments and construct the CI on this basis. Otherwise, we typically used Bayesian bootstrapping, wherein a posterior sample of estimates is obtained by repeatedly computing weighted averages, with the weights sampled from a uniform Dirichlet distribution^71^.

There are several technical details consistent throughout our approach to inference. We use the JAX scientific library to compute the gradients and Hessian matrices of our probability densities, which we need to optimize and quantify uncertainty in our model. We always work with log probabilities for the sake of numerical stability. Despite this, loss of numerical precision occasionally results in nonpositive semidefinite covariance matrices (that is, matrices with eigenvalues less than zero). In this case, we follow theorem 3.2 from Cheng and Higham^72^ to identify the nearest positive semidefinite matrix to *Σ* with eigenvalues greater than 10^−5^, which amounts to setting all eigenvalues less than 10^−5^ to this cutoff.

### Predicting the effects of deletions and ssASOs

To evaluate the effects of deletions and ASOs, we computed predictions for modified sequences. We assume that an ASO blocks SF interactions with the positions bound and one base on either side and we treat these positions as if they were the lowest-affinity nucleotide when scoring with the binding model. For deletions, we simply score the sequence with the deletion. We use the posterior mean binding and activity parameters to construct our predictions. Because we wanted to predict how each ASO or deletion alters regulation, rather than compute splicing impacts as

$$\Delta {{{A}}}_{i}={\mathop{\sum }\limits_{j=1}^{J}}B({{{S}}}_{ij};\lambda ,m,d){\alpha }_{j},$$

we instead computed predicted regulation for a given SF on the basis of occupancy:

$${{{A}}}_{i}=\mathop{\sum }\limits_{j=1}^{J}\Theta ({{{a}}}_{ij};F){\alpha }_{j},$$

where ${{{a}}}_{ij}={\rm{log}}\;{\rm{sum}}\;{\rm{exp}}(\lambda {{{S}}}_{ij})$ is the total affinity in bin *j*. We compute the change in regulation because of deletion or ASO *i* as

$$\Delta {{{A}}}_{i}=\frac{1}{\max (| \alpha | )}\mathop{\sum }\limits_{j=1}^{J}{\alpha }_{j}\left[\Theta \left({{{a}}}_{ij};F\right)-\Theta \left({{{a}}}_{0j};F\right)\right],$$

where *a*_0*j*_ is the affinity of region *j* in the reference sequence and *a*_*ij*_ is the affinity given the deletion or ASO. To compare changes in regulation across models, we normalize the predictions by the maximum change in regulation that could result from a complete loss of occupancy at a binding site (that is, $\max (| \alpha | )$). Doing this requires assuming a free protein concentration, *F*, which is not known a priori. As a proxy, we use estimates of free protein concentration in the cells from which the models were estimated based on the learned most relevant affinity, *m*, and assuming free protein decreased by half after knockdown:

$$F=\frac{1}{\sqrt{k}{e}^{m}},$$

with $k=\frac{1}{2}$. Our conclusions are not sensitive to choice of *k*, with $k=\frac{1}{2},\frac{1}{4},\frac{1}{8}$ all predicting similar disruptions to regulation.

### Constructing mutant sequences

For each selected exon, we identified the 10-nt region most strongly contributing to the splicing impact (Fig. 4a,b). We then treated all regions that were at least half as impactful as potential SREs. For each SRE, we identified the three single-nucleotide polymorphisms most disruptive to binding. For exons with both up and downstream SREs, we constructed separate sequences with the upstream or downstream mutations alone, as well as with both upstream and downstream mutations.

### Cell culture

HepG2 cells (American Type Culture Collection) were grown in DMEM (Gibco) supplemented with 10% bovine growth serum (Gibco) and 1× penicillin–streptomycin (Gen Clone, 25-512) in a 37 °C incubator under 5% CO_2_.

### Minigene splicing reporter constructs

RHCglo minigene reporter plasmid DNA (Addgene, plasmid 80169) was digested with SalI-HF (New England Biolabs (NEB), R3138S) and XbaI (NEB, R0145S) restriction enzymes to remove synthetic exon and flanking human β-globin intronic and SS sequence. Digests were performed in a single reaction according to the manufacturer’s instructions and then purified using DNA clean and concentrator kit (Zymo, D4003). DNA constructs consisting of target exonic sequences + 250 nt of flanking upstream and downstream intronic sequences and sequences overlapping the RHCglo sequence (Supplementary Table 5) were synthesized by Twist Bioscience and inserted into the linearized RHCglo pDNA using NEBuilder HiFi DNA assembly master mix (NEB, E2621S). Reactions were used to transform Mix N Go DH5α cells (Zymo). For NF2 exon 16 mutant reporters used in the RBFOX2–QKI coregulation analysis sequence changes were introduced to the wild-type minigene using the QuickChange II site-directed mutagenesis kit (Agilent, 200523) following the manufacturer’s instructions and transformed into XL1-Blue supercompotent cells. Plasmid DNA from successful transformants were purified using Qiagen Spin miniprep kit (Qiagen, 27104). RHCglo was a gift from T. Cooper.

### SF knockdown and minigene experiments

For small interfering RNA (siRNA) knockdown minigene reporter experiments, 12-well plates were seeded 24 h in advance with 0.1 × 10^6^ HepG2 cells. Cells were treated for 48 h with 50 pmol of siRNA pool targeting the SF of interest (Supplementary Table 5) or ON-TARGETplus siRNA control pool (Dharmacon) using RNAiMAX reagent (Invitrogen). At 48 h, the medium was removed and cells were transfected with 50 ng of minigene reporter pDNA using Lipofectamine 3000 reagent and incubated for 24 h to allow transcript expression. Culture medium was removed, cells were washed twice with 1× PBS pH 7.4 and total RNA was extracted using the RNeasy mini kit (Qiagen, 74106) according to the manufacturer’s instructions. Each experiment was performed as three biological replicates. The PCPB1 upstream mutant reporter assay was performed without the siRNA knockdown step as a single replicate and not used in later experiments because of the impact of mutations on the 3SS strength.

### Minigene splicing quantification by semiquantitative reverse transcription (RT)–PCR

First-strand complementary DNA (cDNA) was generated from 100–150 ng total of extracted RNA using an Oligo d(T)_20_primer (Invitrogen, 18418020) and SuperScript IV reverse transcriptase (Invitrogen, 18090050) according to the manufacturer’s instructions. cDNA was PCR-amplified using RHCglo gene product specific primers RSV5U and RTRHC^73^ (universal to all constructs) and Q5 high-fidelity 2× master mix (NEB, M0492S) for 28 cycles. PCR products were resolved on 3% agarose gels and the band intensity for exon inclusion and exon skipping isoforms was quantified using the ImageJ (version 1.53k) software package. PSI was calculated as band intensity of the inclusion isoform divided by the total intensity of both isoforms.

### Confirmation of SF knockdown by western blot

HepG2 cells were treated with each siRNA and minigene combination as described above. Cells were washed with 1× PBS and then lysed in RIPA buffer (50 mM Tris pH 7.0, 150 mM NaCl, 0.1% SDS, 0.5% sodium deoxycholate and 1× Triton X-100). Protein from each lysate was resolved on Bis–Tris NuPAGE acrylamide gels (Invitrogen) and then transferred to a iBlot nitrocellulose membrane (IB4301001) using the iBLOT dry blot system. Membranes were blocked for 1 h in Azure fluorescent blot blocking buffer (Azure Biosystems). Rabbit primary antibodies for each SF (Supplementary Table 5) at 1:1,000 dilution were added to the membranes and incubated at 4 °C overnight with gentle agitation. Membranes were washed with 1× TBST and then incubated with fluorescently labeled secondary antibody (Supplementary Table 5) at a 1:10,000 dilution for 1 h at room temperature, washed with 1× TBST and visualized using Azure c600 (Azure Biosystems). Each membrane was then incubated with mouse anti-α-tubulin (1:2,500) for 1 h at room temperature, washed with 1× TBST, incubated with secondary antibody (Supplementary Table 5) at room temperature for 1 h, washed and visualized. Band intensity was quantified using ImageJ software and the knockdown efficiency was calculated as α-tubulin normalized intensity of the SF’s band in the SF siRNA-treated cells over the control siRNA-treated cells (Supplementary Table 5).

### Computational resources

Analyses were performed on a Dell Precision 7740 laptop with an NVIDIA Quadro RTX 3000 GPU (5.9 GB, CUDA version 11.4), 16 CPUs (Intel(R) Xeon(R) E-2286M, 2.40 GHz) and 67.2 GB of RAM plus 137.4-GB swap and a Dell Precision 7960 tower with an NVIDIA Ada RTX 5000 GPU (33.7 GB, CUDA version 12.2), 72 CPUs (Intel(R) Xeon(R) w9-3475X) and 201.7 GB of RAM.

### Reporting summary

Further information on research design is available in the Nature Portfolio Reporting Summary linked to this article.

## Online content

Any methods, additional references, Nature Portfolio reporting summaries, source data, extended data, supplementary information, acknowledgements, peer review information; details of author contributions and competing interests; and statements of data and code availability are available at https://doi.org/10.1038/s41587-025-02881-9.

## Supplementary information

Supplementary Information Supplementary Methods.

Reporting Summary

Supplementary Table 1 High-level overview of the KATMAP analyses. For each analysis performed, the source and accession of the differential splicing results and the motif models are provided, as well as descriptions of exons used to train the model. High-level summaries of the results are reported as the model comparison *z* scores and numbers of significant activity coefficients, along the average number of predicted binding changes per exon. Whether an analysis yielded a significant activity map is indicated for each analysis and, if a given SF had both an RBNS-based PSAM and a PWM, we indicate which model we used in our analyses.

Supplementary Table 2 Splicing impacts computed for all cassette exons in the ENCODE knockdowns for each SF with a significant activity map. Each row describes an exon, with the chromosomal coordinates denoting the first and last nucleotide of the exon, along with the splicing impacts assigned to that exon from KATMAP’s activity models for the different SFs. If an SF gave a significant activity model in more than one knockdown, we include the splicing impacts from the model with strongest evidence of splicing activity.

Supplementary Table 3 Summary of exons identified as being coregulated by QKI and RBFOX on the basis of QKI (HepG2) and RBFOX2 (HepG2) knockdowns and the mouse *Rbfox1/2/3* triple-knockout data.

Supplementary Table 4 SF perturbations inferred using KATMAP and the inclusion-based approach, along with the DESEQ estimates of SF expression variation. Each row represents a knockdown experiments and the columns store the estimates for different SFs. The ashr shrinkage estimates, adjusted *P* values, raw estimates and associated s.d. are provided in separate sheets.

Supplementary Table 5 Details on the minigene splicing assays. Contains sequences for semiquantitative RT–PCR primers. The table also includes siRNAs, primary and secondary antibodies used in minigene reporter experiments. The final sheet contains the name and measured knockdown efficiency for each RBP and minigene combination normalized to α-tubulin, as well as coordinates (hg19) and exonic/intronic sequences used to construct minigene reporters.

## Source data

Source Data Fig. 2 Statistical source data.

Source Data Fig. 3 Statistical source data.

Source Data Fig. 4 Statistical source data.

Source Data Fig. 5 Statistical source data.

Source Data Fig. 6 Statistical source data.

Source Data Extended Data Fig. 1 Statistical source data.

Source Data Extended Data Fig. 2 Statistical source data.

Source Data Extended Data Fig. 3 Statistical source data.

Source Data Extended Data Fig. 4 Statistical source data.

Source Data Extended Data Fig. 5 Statistical source data.

Source Data Extended Data Fig. 6 Statistical source data.

Source Data Extended Data Fig. 7 Statistical source data.

## Data Availability

Pretrained models, inference summaries and tables of target predictions are available from Zenodo (https://doi.org/10.5281/zenodo.15298341)^74^. Source data are provided with this paper.

## Code availability

The KATMAP library (along with pretrained models) is available from GitLab (https://gitlab.com/LaptopBiologist/katmap).
