# Supplementary material for: KATMAP infers splicing factor activity and regulatory targets from knockdown data
Source: Nat Biotechnol. 2025 Nov 4;44(11):1934–47. doi: 10.1038/s41587-025-02881-9 (PMC13751310; doi:10.1038/s41587-025-02881-9)
Supplement: Supplementary file 1 [file 41587_2025_2881_MOESM1_ESM.pdf]

nature biotechnology

Supplementary information https://doi.org/10.1038/s41587-025-02881-9

KATMAP infers splicing factor activity and regulatory targets from knockdown data

In the format provided by the authors and unedited

1 Extended methods

1.1 Adaptive importance sampling from the marginal posterior

We employ an adaptive importance sampling procedure to the explore the set of plausible hyperparameter values. Before describing the details of this procedure, it is important to note that we work with unconstrained representations of the hyperparameters. That is, for the length-scale L and stationary scale σ, we use their log-transformed values. If we denote the hyperparameters $\varphi_i = (m_i, \lambda_i, L_i, \sigma_i)$, then we denote unconstrained version as $\tilde{\varphi}_i = (m_i, \lambda_i, \log L_i, \log \sigma_i)$. Computing the probability density on the unconstrained hyperparameters requires accounting for how the transformation stretches or compresses the density, so $P(\tilde{\varphi}) = \frac{P(\varphi)}{JacDet(\varphi)}$ where $JacDet$ is the Jacobian determinant of the transform. In practice, this amounts to multiplying the constrained density by $L_i \times \sigma_i$.

We begin using the unconstrained version of the prior as our first proposal distribution

$$G^{(1)}(\tilde{\varphi}_i) = P(\varphi_i) \times L_i \times \sigma_i$$

From this, we sample n unconstrained hyperparameters, $\tilde{\varphi}_1^{(1)}, .., \tilde{\varphi}_n^{(1)}$ and compute the importance weights which describe how the proposal distribution differs from the target marginal posterior $P(\tilde{\varphi}|Y)$:

$$w_i^{(1)} = \frac{P(\varphi_i^{(1)}|Y) \times L_i^{(1)} \times \sigma_i^{(1)}}{G^{(1)}(\tilde{\varphi}_i^{(1)})}$$

We use these evaluation points to estimate a new proposal distribution, $G^{(2)}$ that better matches the target posterior, with the adaptation procedure rooted in fitting a Student's T mixture model (TMM) using importance weighted expectation-maximization (IW-EM) [1], described below. From this, we generate and evaluate a new sample of hyperparameters. We repeat this procedure, obtaining a series of proposal distributions that approach the target, until we are confident that we will draw a sufficiently large sample from the target.

At each iteration T, this yields a set of evaluation points drawn from different proposal distributions

$$\tilde{\varphi}_1^{(t=1)}, \tilde{\varphi}_2^{(t=1)}, .., \tilde{\varphi}_n^{(t=1)} \sim G^{(t=1)}$$

$$\tilde{\varphi}_1^{(t=2)}, \tilde{\varphi}_2^{(t=2)}, .., \tilde{\varphi}_n^{(t=2)} \sim G^{(t=2)}$$

$$...$$

$$\tilde{\varphi}_1^{(t=T)}, \tilde{\varphi}_2^{(t=T)}, .., \tilde{\varphi}_n^{(t=T)} \sim G^{(t=T)}$$

Each proposal distribution contributed equally to the set of generated evaluation points, so we compute the proposal probabilities from an equally weighted mixture of all past proposal distributions

$$G_{full}^{(T)}(\tilde{\varphi}) = \frac{1}{T} \sum_{t=1}^{T} G^{(t)}(\tilde{\varphi})$$

Then the raw importance weights are

$$w_i^{(t)} = \frac{P\left(\tilde{\varphi}_i^{(t)}|Y\right)}{G_{full}^{(T)}\left(\tilde{\varphi}_i^{(t)}\right)}$$

and the raw acceptance probabilities are

$$\pi_i^{(t)} = \frac{w_i^{(t)}}{\max\limits_{j,s} \quad w_j^{(s)}}$$

Following Vehtari et al [2], we apply a stabilizing correction to these importance weights, which we describe below, to obtain stabilized importance weights and acceptance probabilities, $\hat{\pi}_{1..n}^{(1..T)}$.

From these acceptance probabilities, we can determine what fraction of samples we expect to accept if we were to stop at the current iteration

$$f_{acc} = \frac{1}{nT}\sum_{i=1}^{n}\sum_{t=1}^{T}\hat{\pi}_i^{(t)}$$

which also provides an estimate of the effective sample size

$$n_{eff} = n f_{acc}$$

To determine whether we can stop iterating, we ask whether there is at least a 99% probability the number of accepted samples n_{acc} exceeds the desired sample size n_{final}. The number of samples we will accept,n_{acc}, is the sum of independent Bernoulli trials with variable success probabilities and so follows a Poisson-binomial distribution. As this distribution is never more variable than a binomial distribution with the same mean and number of trials, we use the binomial distribution to obtain a conservative estimate of the lower bound on the number of proposal that will be accepted, n_{acc}, considering importance sampling complete once

$$P(n_{acc} \geq n_{final}) \geq .99, \qquad n_{acc} \sim Binomial(N = nT, p = f_{acc})$$

Upon termination, we subsample (without replacement) the proposed hyperparameters according to the stabilized acceptance probabilities to obtain our sample of hyperparameters from from the marginal posterior.

1.2 Adapting the proposal distribution

We first use the EM procedure described below to fit a Student's T mixture model (TMM) to the target, obtaining mixing weights $\omega_{1..C}$, means $\mu_{1..C}$, and covariance matrices $\Sigma_{1..C}$, as well as a degree-of-freedom parameter ν shared by all C component distributions. This provides an estimate of the target distribution that can account for both its local structure and the heaviness of its tails. However, in the first few iterations of adaptive importance sampling the effective sample is typically small (< 5) and we found the TMM-based proposal to occasionally become too concentrated around the modes of the target density, requiring extra rounds of adaption to properly explore tails. To decrease the time to convergence, we employ two heuristics to stabilize the adaptation in the early rounds of importance sampling. Both heuristics aim to ensure the proposal is distributed around the target density, but always somewhat more dispersed than the true target.

First, we double the fitted the covariance matrices, defining the mixture distribution which captures the local structure of the target density as

$$TMM_{local}(x) = \sum_{c=1}^{C}\omega_c \mathcal{T}\left(x; \nu, \mu_c, 2\Sigma_c\right)$$

By intentionally making the proposal broader than the IW-EM estimate of the target density, we sacrifice somewhat the efficiency with which we obtain samples from the target density. However, this ensures that subsequent evaluation points fall in the tails of the target, decreasing the risk that the adaptation overshoots.

However, when the effective sample from the target is small, we lack precise information about the finer details of the distribution's shape and TMM_{local} may still overfit. We therefore construct an even more conservative proposal. When n_{eff} is close to zero, IW-EM will shift the means in the direction of a mode in the target, but will learn little about the target's covariance. Given that covariance of the target remains obscure, we may as well continue to use the previous proposal distribution. We approximate this by computing the covariance of the proposals $\hat{\Sigma}_{proposal}$. If n_{eff} is somewhat larger than zero, we might have enough information to estimate the global covariance of the target, $\hat{\Sigma}_{target}$, which we can obtain by computing an importance weighted covariance from the proposals. To find a intermediate between these two extremes, we take a weighted average that approaches $\hat{\Sigma}_{target}$ as n_{eff} increases

$$\hat{\Sigma} = \frac{1}{1+n_{eff}}\left(\hat{\Sigma}_{proposal} + 2 \times n_{eff} \times \hat{\Sigma}_{target}\right)$$

The Bayesian flavor of transitioning from an agnostic *a priori* assumption to a data-driven estimate as more information becomes available is not coincidental. This estimator can be thought of as being twice the posterior mean, Σ', of the target covariance estimated using an inverse-Wishart Gaussian conjugate model with a prior scale matrix based on the initial proposal distribution, $\Psi_0 = \frac{1}{2}\hat{\Sigma}_{proposal}$, and $\nu_0 = p+2$ degrees of freedom, where p is the number of rows/columns in the covariance matrix,

$$\Sigma' = \frac{1}{n+\nu_0-p-1}\left(\Psi_0 + n\Sigma\right) = \frac{1}{1+n}\left(\frac{1}{2}\hat{\Sigma}_{proposal} + n\Sigma\right)$$

$$\hat{\Sigma} = 2\Sigma'$$

where Σ is a covariance matrix computed from n data points.

From this, we construct our more conservative proposal distribution

$$TMM_{global}(x) = \sum_{c=1}^{C} \omega_c \mathcal{T}\left(x; \nu = 35, \mu_c, \hat{\Sigma}\right)$$

This uses the IW-EM estimates of target's location, but replaces the covariances with a global estimate of the target's covariance, to ensure new proposals explore the target density's tails.

Our final proposal overlays these as a mixture, with the mixing weights depending upon n_{eff}

$$G(x) = \frac{n_{eff}}{\rho+n_{eff}} TMM_{local}(X) + \frac{\rho}{\rho+n_{eff}} TMM_{global}(X)$$

As n_{eff} increases, the composition of the mixture shifts from the conservative TMM_{global} to TMM_{local}. We choose $\rho = 4$ such that when $n_{eff} = 1$, 80% of the new proposals come from the conservative mixture, at $n_{eff} = 20$ it is about 16% proposals, and when $n_{eff} = 100$ the proposal is largely derived from the local TMM, with less than 4% of proposal derived from the conservative mixture. Whatever n_{eff}, the updated proposal always moves towards modes of the target density, because both TMM_{local} and TMM_{global} use the means and mixing proportions estimated from IW-EM.

1.3 Importance-weighted Expectation Maximization for a multivariate T-mixture

To construct proposals that can robustly adapt to the target, we follow Cappé et al. [1] and fit a TMM by importance weighted Expectation-Maximization (IW-EM). One approach to conceptualizating a multivariate T distribution is a scale-mixture of multivariate Gaussians. So a mixture of T distributions can be described as a mixture of Gaussians with two latent variables per realization x_i

$$z_i \sim Categorical(\omega_{1..K})$$

$$\gamma_i \sim Gamma\left(\frac{\nu_{z_i}}{2}, \frac{\nu_{z_i}}{2}\right)$$

$$x_i \sim MvNormal\left(\mu_{z_i}, \frac{\Sigma_{z_i}}{\gamma_i}\right)$$

where ν is the degrees of freedom. $\omega_{1..K}$ is a vector of mixing weights describing the fraction of the full mixture each component distribution contributes. $\mu_{1..K}$ and $\Sigma_{1..K}$ are means and covariances of the K component distributions. $z_{1..N}$ is vector of unobserved variables indicating which component each observation x_i truly derives from. The second set of unobserved variables $\gamma_{1..N}$ come from the scale-mixture representation of the Student T distribution and describe how each Gaussian z_i was rescaled to generate each realization x_i.

While, Cappé et al. [1] initialize once and perform a single EM-update at each step of importance sampling, we instead reinitialize at each importance sampling iteration t and perform t EM-updates to

prevent the adaptive distribution from getting trapped. We use K-means clustering of the proposals with $10 \times t$ components, to define the initial means ensure the components are well-distributed throughout the proposal distribution.

1.3.1 Expectation Step

For each observed x_i, there are two unobserved variables, $z_{1..N}$ and $\gamma_{1..N}$. The expectation step infers these latent variables by computing their expected values given the parameters of the mixture distribution $(\omega_{1..K}, \mu_{1..K}, \Sigma_{1..K})$.

To estimate the probability that x_i arose from a given component distribution d, we compute

$$\eta_{id} = E\left[z_i = d | X\right] = \frac{\omega_d \mathcal{T}(x_i; \nu_d, \mu_d^{(t)}, \Sigma_d^{(t)})}{\sum_{\tilde{d}} \omega_{\tilde{d}} \mathcal{T}(x_i; \nu_d, \mu_{\tilde{d}}^{(t)}, \Sigma_{\tilde{d}}^{(t)})}$$

where $\mathcal{T}$ denotes the multivariate Student-t probability density function as implemented in scipy.

Then to estimate the scale of the Gaussian from which x_i arose, assuming it belongs to the d-th Student T distribution, we compute

$$u_{id} = E\left[\gamma_i | X, z_i = d\right] = \frac{\nu_d + r}{\nu_d + \left(x_i - \mu_d^{(t)}\right)^T \left(\Sigma_d^{(t)}\right)^{-1} \left(x_i - \mu_d^{(t)}\right)}$$

where r is the dimension of the distribution (in our case, the number of hyperparameters in the additive model).

1.3.2 Maximization Step

The maximization step uses these estimates of the latent variables to refine the estimates of the mixture distribution's parameters. These estimates are essentially averages weighted by the component probabilities η_{id}. To fit the mixture to the target rather than the proposals, we follow Cappé et al. [1] and compute the updates as weighted averages using the importance weights $\pi_{i..N}$ to weight the observations:

$$\omega_j^{(t+1)} = \frac{\sum_i \eta_{ij} \pi_i}{\sum_{\tilde{i}} \pi_{\tilde{i}}}$$

$$\mu_j^{(t+1)} = \frac{\sum_i \eta_{ij} \pi_i x_i}{\sum_{\tilde{i}} \eta_{\tilde{i}j} \pi_{\tilde{i}}}$$

$$\Sigma_j^{(t+1)} = \frac{\sum_i \eta_{ij} u_{ij} \pi_i \left(x_i - \mu_j^{(t+1)}\right) \left(x_i - \mu_j^{(t+1)}\right)^T}{\sum_{\tilde{i}} \eta_{\tilde{i}j} \pi_{\tilde{i}}}$$

The degree-of-freedom parameter, ν, controls the tails of the distribution. There is no closed-form estimate of ν and so it must be estimated by optimization. To make this tractable, we constrain the components of the mixture to all share the same ν parameter. By reducing this to a single parameter, we can use a binary search to update $\nu^{(t+1)}$, considering values between $\nu \in (4, 100)$. The importance weighted EM scheme optimizes the entropy of the normalized importance weights we would get if we used the mixture as the proposal distribution

$$w_i = \frac{P(x_i)}{\sum_{j=1}^{K} \omega_j^{(t+1)} \mathcal{T}\left(x_i; \nu^{(t+1)}, \mu_j^{(t+1)}, \Sigma_j^{(t+1)}\right)}$$

$$\bar{w}_i = \frac{w_i}{\sum_{\tilde{i}}^{N} w_{\tilde{i}}}$$

$$\mathbf{H} = -\sum_i^N \bar{w}_i log(\bar{w}_i)$$

So we evaluate the entropy at twenty values of ν between 4 and 100 and identify all local maxima on this grid. We then perform binary searches between the neighbors of each maxima to hone in on the true local maxima and set $\nu^{(t+1)}$ to the value of ν that yielded the highest entropy.

1.3.3 Stabilizing the importance weights

The set of importance weights $\pi_{1..n}^{(1..T)}$ represent an estimate of how the proposal distribution differs from the target distribution. If the proposal distribution is very different from the target, the computed importance weights will poorly characterize the target and importance sampling will be inefficient or untrustworthy.

Vehtari et al provide an approach for obtaining more stable estimates of the importance weights by fitting a Pareto distribution to the tail of $\pi_{1..n}^{(1..T)}$ [2]. Using the implementation of this Pareto smoothing from the arviz library [3], we compute smoothed weights $\hat{\pi}_{1..n}^{(1..T)}$. Importantly, the shape parameter of the fitted Pareto distribution, $\hat{k}$, is diagnostic of whether importance sampling will be reliable.

We only accept posterior samples based on the Pareto-smoothed importance weights. Occasionally in the early iterations of adapting the proposal distribution, however, the range of values computed for the importance weights may be too extreme for Pareto smoothing to yield reliable importance estimates (e.g. $\hat{k} > 0.7$). In these cases, we use an older, simpler approach to stabilize the weights [4]. This defines a cutoff based on the total number of importance weights N_π and average importance weight $\bar{\pi}$

$$\gamma = \bar{\pi}\sqrt{N_\pi}$$

and then replaces all importance weights greater than γ with γ

$$\hat{\pi}_i = \begin{cases} \pi_i & \text{if } \pi_i < \gamma \\ \gamma & \text{if } \pi_i \geq \gamma \end{cases}$$

The way in which this flattens the importance weights will bias the importance estimates by accepting some proposals which should be rejected. When used to adapt the proposal distribution, this should result in an estimate of the target distribution that is broader than the true target. This decreases the efficiency with which the proposal is adapted, but prevents it from collapsing to a subset of the target distribution.

1.3.4 Assumptions about the spatial correlations: L, σ

There are two identifiability issues that can arise with Gaussian process priors. If the length-scale L is near zero, it corresponds to the biologically implausible assumption of complete spatial independence of the activity coefficients. But it also introduces problems for statistical inference. As we perform inference with respect to the log-transform of L, all values of $logL \ll 0$ are essentially equivalent. Similarly, length-scales much larger than the maximum distance between regions analyzed $L \gg \max(|j-k|)$ are practically indistinguishable.

To prevent the inference algorithm from wasting computation by exploring large but practically equivalent regions of parameter space, we construct a prior that avoids both large and small values of L. To avoid overly large length-scales, we use the right-tail of a generalized gamma distribution

$$GG(x; a, d, p) = \Gamma\left(\frac{d}{p}\right)\frac{p}{a^d}x^{d-1}exp\left(\left(\frac{x}{a}\right)^p\right)$$

This does not prevent L from being close to zero. We avoid these small values by convolving the generalized gamma with the left tail of an inverse-gamma distribution

$$HGG(x; a, d, p, b) = \frac{1}{Z(a, d, p, b)}exp\left(-\frac{b}{x}\right)GG(x; a, d, p)$$

where the normalizing constant is computed numerically with scipy.integrate.quad

$$Z(a, d, p, b) = \int_0^\infty exp\left(-\frac{b}{x}\right)GG(x; a, d, p)$$

We set the prior on the length-scale as

$$L \sim HGG(x; a = 20, d = .01, p = 5, b = 1)$$

which places 99% of the prior probability on the interval $0.32 < L < 22.6$.

We use this same distribution to exclude small values of the stationary standard deviation. If σ is close to zero, all of the activity coefficients become constrained to be close to zero solely by the prior. One way of conceptualizing the prior on σ is that it reflects different assumptions we might make about the regression coefficients in our regression analysis. We would never perform a regression analysis where we assumed *a priori* that all of the regression coefficient were zero. Whether the coefficients should be close to zero is something we wish to learn from the data, not assume in advance. So we define our prior on the stationary standard deviation as

$$\sigma \sim HGG(x; a = 2, d = .25, p = 5, b = .5)$$

which places 99% of the prior probability on the interval $0.14 < \sigma < 8.7$

1.4 Model comparison against the reduced model

To evaluate whether the data support splicing activity, we compare the full model against a reduced model without splicing activity. To verify that any improvement over the reduced model did not reflect overfitting to the training data, we sought to evaluate each model's performance on held-out data. However, to sidestep the need to rerun the inference algorithm dozens of times for each knockdown, we employ the Pareto-smoothed importance sampling approximation to leave-one-out (PSIS-LOO) cross-validation proposed by Vehtari et al. [2]

PSIS-LOO uses the posterior sample from training on all data as a proposal distribution to obtain estimates regarding the leave-one-out posterior distribution that would be obtained by excluding the i-th observation. It leverages the insight that the ratio between the complete and the leave-one-out posteriors depends only on the left-out observation, with all other terms in the posteriors cancelling out.

$$r_i(\theta) \propto \frac{P(\theta|Y_{-i})}{P(\theta|Y)}$$

$$\propto \frac{P(Y_1|\theta) \times .. \times P(Y_{i-1}|\theta) \times P(Y_{i+1}|\theta) \times .. \times P(Y_N|\theta) \times P(\theta)}{P(Y_1|\theta) \times .. \times P(Y_{i-1}|\theta) \times \boldsymbol{P(Y_i|\theta)} \times P(Y_{i+1}|\theta) \times .. \times P(Y_N|\theta) \times P(\theta)}$$

$$\propto \frac{1}{P(Y_i|\theta)}$$

These importance weights are then stabilized using the aforementioned Pareto smoothing to obtain $\tilde{r}_i(\theta)$. We estimate the log expected posterior predictive probability for each held-out exon i by computing the importance-weighted average over the complete posterior sample

$$lpd_i = \log\left(\frac{1}{\sum_{k=1}^{M} \tilde{r}_i(\theta_k)} \sum_{j=1}^{M} \tilde{r}_i(\theta_j) P(Y_i|\theta_j)\right)$$

To combine this across all held-out exons, we sum the estimated log-probabilities, exactly as we would when computing the model's objective function

$$\widehat{elpd} = \sum_{i=1}^{N} lpd_i$$

We chose this estimator as it is the finite sample version of the widely-applicable information criterion (WAIC). We compute this for the full, $\widehat{elpd}_{Full}$, and reduced models, $\widehat{elpd}_{\emptyset}$, and summarize the model comparison as $\widehat{MC} = \widehat{elpd}_{Full} - \widehat{elpd}_{\emptyset}$. A positive value of $\widehat{MC}$ means the full model with splicing activity is a better explanation of the data than the reduced model without splicing activity. To quantify how confident we are in this evidence for splicing activity, we bootstrap the exons 10,000 times, estimating $\widehat{MC}$ each time, and use these obtain a standard deviation $SD(\widehat{MC})$. We then compute a Z-score

$$Z(\widehat{MC}) = \frac{\widehat{MC}}{SD(\widehat{MC})}$$

1.5 Working with differential splicing analyses

1.5.1 Recovering effect sizes estimates from rMATS outputs

rMATs reports the inclusion levels and inclusion level differences using direct summaries of the read counts, rather than the effect sizes inferred by the underlying model. The actual parameters of the rMATS model, however, are defined in log-odds scale. This log-odds representation allows interval estimates to be naturally defined around the effect size in a way that respects the constraint that $0 < \psi < 1$. The read count based summaries, however, may equal 0 or 1, and cannot be converted as is to log-odds scale. To resolve this, we recompute PSI with pseudocounts added to the observed reads, which is equivalent to estimating the inclusion read fraction with a beta-binomial model.

$$f_i = \frac{a + IJC_i}{a + b + IJC_i + SJC_i}$$

We identify the appropriate pseudocounts from the data itself, fitting a beta-binomial distribution to the read counts of all exons. We then estimate the inclusion read fraction for each exon and convert these to PSI

$$\psi_i = \frac{f_i L_i^{(S)}}{f_i L_i^{(S)} + (1 - f_i) L_i^{(I)}}$$

where the $L_i^{(I)}$ and $L_i^{(S)}$ account for the differing amounts of informative sequence associated with the inclusion and exclusion junctions respectively.

1.5.2 Reconstructing uncertainty about effect size from rMATS outputs

We sought to reconstruct the posterior standard deviation for these effect sizes from the rMATS p-values. rMATS computes its p-values using a likelihood ratio test, comparing the likelihood of the full model to that where the effect size is set to a small value near zero, $LR = \frac{Lk_\emptyset}{Lk_{full}}$. The test statistic $\Lambda_i = -2logLR$ follows a chi-squared distribution and can be recovered by inputting the rMATS p-value, p_i for exon i into the chi-squared inverse cumulative density function

$$\Lambda_i = CDF^{-1}_{\chi^2(df=1)}(1 - p_i)$$

Describing the uncertainty about the effect sizes with a mean and standard deviation implies a normal approximation to the likelihood. The likelihood-ratios, therefore, can be thought of as comparing the density of this normal distribution at its mode to a point in its tail. The likelihood of the full model is evaluated at its mode (e.g. effect size)

$$L^{full} = \frac{1}{\sigma_i\sqrt{2\pi}} exp\left[-\frac{(\delta_i - \delta_i)^2}{2\sigma_i^2}\right] = \frac{1}{\sigma_i\sqrt{2\pi}}$$

and is entirely determined by the standard deviation. The null model, however, evaluates the likelihood at a point away from the mode, $\delta_\emptyset$, assuming the effect size is close to zero

$$L^{null} = \frac{1}{\sigma_i\sqrt{2\pi}} exp\left[-\frac{(\delta_\emptyset - \delta_i)^2}{2\sigma_i^2}\right]$$

The likelihood ratio is then

$$LR_i = \frac{\frac{1}{\sigma_i\sqrt{2\pi}} exp\left[-\frac{(\delta_\emptyset - \delta_i)^2}{2\sigma_i^2}\right]}{\frac{1}{\sigma_i\sqrt{2\pi}}} = exp\left[-\frac{(\delta_\emptyset - \delta_i)^2}{2\sigma_i^2}\right]$$

and the negative log-likelihood ratio is

$$logLR = -\frac{(\delta_\emptyset - \delta_i)^2}{2\sigma_i^2}$$

To get the variance σ_i^2 of the normal approximation we can rearrange

$$\sigma_i^2 = \frac{(\delta_\emptyset - \delta_i)^2}{-2logLR}$$

The Chi2 test statistic, $\Lambda_i = -2logLR_i$, is the denominator so this can be written:

$$\sigma_i^2 = \frac{(\delta_\emptyset - \delta_i)^2}{\Lambda_i}$$

References

[1] Cappé, O., Douc, R., Guillin, A., Marin, J.-M. & Robert, C. P. Adaptive importance sampling in general mixture classes. *Stat Comput* **18**, 447–459 (2008-12-01). URL https://doi.org/10.1007/s11222-008-9059-x.

[2] Vehtari, A., Simpson, D., Gelman, A., Yao, Y. & Gabry, J. Pareto smoothed importance sampling. *Journal of Machine Learning Research* **25** (2024) 25 1–58. URL http://jmlr.org/papers/v25/19-556.html.

[3] Kumar, R., Carroll, C., Hartikainen, A. & Martin, O. ArviZ a unified library for exploratory analysis of bayesian models in python. *Journal of Open Source Software* **4**, 1143 (2019-01-15). URL https://joss.theoj.org/papers/10.21105/joss.01143.

[4] Ionides, E. L. Truncated importance sampling. *Journal of Computational and Graphical Statistics* **17** 2 (2008-06-01). URL https://www.tandfonline.com/doi/abs/10.1198/106186008X320456.
