# Supplementary material for: KATMAP infers splicing factor activity and regulatory targets from knockdown data
Source: Nat Biotechnol. 2025 Nov 4;44(11):1934–47. doi: 10.1038/s41587-025-02881-9 (PMC13751310; doi:10.1038/s41587-025-02881-9)
Supplement: Supplementary file 2 [file 41587_2025_2881_MOESM2_ESM.pdf]

nature portfolio

Corresponding author(s): Michael P McGurk, Christopher B. Burge

Last updated by author(s): Sep 19, 2025

Reporting Summary

Nature Portfolio wishes to improve the reproducibility of the work that we publish. This form provides structure for consistency and transparency in reporting. For further information on Nature Portfolio policies, see our Editorial Policies and the Editorial Policy Checklist.

Statistics

For all statistical analyses, confirm that the following items are present in the figure legend, table legend, main text, or Methods section.

n/a	Confirmed	
☐	☒	The exact sample size (*n*) for each experimental group/condition, given as a discrete number and unit of measurement
☐	☒	A statement on whether measurements were taken from distinct samples or whether the same sample was measured repeatedly
☐	☒	The statistical test(s) used AND whether they are one- or two-sided<br>*Only common tests should be described solely by name; describe more complex techniques in the Methods section.*
☒	☐	A description of all covariates tested
☐	☒	A description of any assumptions or corrections, such as tests of normality and adjustment for multiple comparisons
☐	☒	A full description of the statistical parameters including central tendency (e.g. means) or other basic estimates (e.g. regression coefficient) AND variation (e.g. standard deviation) or associated estimates of uncertainty (e.g. confidence intervals)
☐	☒	For null hypothesis testing, the test statistic (e.g. *F*, *t*, *r*) with confidence intervals, effect sizes, degrees of freedom and *P* value noted<br>*Give P values as exact values whenever suitable.*
☐	☒	For Bayesian analysis, information on the choice of priors and Markov chain Monte Carlo settings
☒	☐	For hierarchical and complex designs, identification of the appropriate level for tests and full reporting of outcomes
☐	☒	Estimates of effect sizes (e.g. Cohen's *d*, Pearson's *r*), indicating how they were calculated

Our web collection on statistics for biologists contains articles on many of the points above.

Software and code

Policy information about availability of computer code

Data collection	ImageJ (v1.53k) was used to quantify the results of the minigene splicing reporter assays
Data analysis	We learned regulatory models using KATMAP (presented in this manuscript, https://gitlab.com/LaptopBiologist/katmap). Differential splicing anlayses were performed with rMATS 4.1.1. Gene ontology analyses were performed using GOrilla.

For manuscripts utilizing custom algorithms or software that are central to the research but not yet described in published literature, software must be made available to editors and reviewers. We strongly encourage code deposition in a community repository (e.g. GitHub). See the Nature Portfolio guidelines for submitting code & software for further information.

Data

Policy information about availability of data

All manuscripts must include a data availability statement. This statement should provide the following information, where applicable:

- Accession codes, unique identifiers, or web links for publicly available datasets
- A description of any restrictions on data availability
- For clinical datasets or third party data, please ensure that the statement adheres to our policy

Pretrained models, inference summaries, and tables of target predictions are available in a Zenodo repository (https://zenodo.org/records/15298342)

Research involving human participants, their data, or biological material

Policy information about studies with human participants or human data. See also policy information about sex, gender (identity/presentation), and sexual orientation and race, ethnicity and racism.

Reporting on sex and gender	Not applicable
Reporting on race, ethnicity, or other socially relevant groupings	Not applicable
Population characteristics	Not applicable
Recruitment	Not applicable
Ethics oversight	Not applicable

Note that full information on the approval of the study protocol must also be provided in the manuscript.

Field-specific reporting

Please select the one below that is the best fit for your research. If you are not sure, read the appropriate sections before making your selection.

☒ Life sciences ☐ Behavioural & social sciences ☐ Ecological, evolutionary & environmental sciences

For a reference copy of the document with all sections, see nature.com/documents/nr-reporting-summary-flat.pdf

Life sciences study design

All studies must disclose on these points even when the disclosure is negative.

Sample size	Minigene experiments were performed in three replicates, as is standard for RT-PCR assays. Replicates represent separate transfections performed on separate days to assess biological variability in inclusion.
Data exclusions	One of the cis-element mutations we considered for our minigene splicing reporter (PCPB1, upstream of the 3'SS) impacted 3'SS strength, so we did not include this minigene in the follow-up knockdowns experiments. We nonetheless included the measured effect of the mutation on inclusion in the Results for transparency, and noted in the Methods that it was excluded from the knockdowns.
Replication	All minigene experiments were performed in three replicates each, representing separate transfections performed on different days, with consistent inclusion levels measured across replicates.
Randomization	Each condition was evaluated in three replicates, each representing separate transfections on separate days. No further randomization was employed.
Blinding	Blinding was not employed, as the results of RT-PCR and Western blots are straightforward measurements and not especially subject to subjective bias.

Reporting for specific materials, systems and methods

We require information from authors about some types of materials, experimental systems and methods used in many studies. Here, indicate whether each material, system or method listed is relevant to your study. If you are not sure if a list item applies to your research, read the appropriate section before selecting a response.

Materials & experimental systems

n/a	Involved in the study	
☐	☒	Antibodies
☐	☒	Eukaryotic cell lines
☒	☐	Palaeontology and archaeology
☒	☐	Animals and other organisms
☒	☐	Clinical data
☒	☐	Dual use research of concern
☒	☐	Plants

Methods

n/a	Involved in the study	
☒	☐	ChIP-seq
☒	☐	Flow cytometry
☒	☐	MRI-based neuroimaging

Antibodies

Antibodies used	Rabbit anti-HNRNPC (MBL Life Science Cat# RN052PW) - used for Western blotting (1:1000 dilution)<br>Rabbit anti-PCBP1 (Proteintech Cat# 14523-1-AP) - used for Western blotting (1:000 dilution)<br>Rabbit anti-QKI (Proteintech Cat# 13169-1-AP) - used for Western blotting (1:1000 dilution)<br>Rabbit anti-RBFOX2 (Proteintech Cat# 12498-1-AP) - used for Western blotting (1:1000 dilution)<br>Rabbit anti-DAZAP1 (Abcam Cat# ab182558) - used for Western blotting (1:1000 dilution)<br>Mouse anti-α-tubulin (Sigma Cat# T5168) - used for Western blotting (1:2500 dilution)<br>Goat anti-rabbit AzureSpectra 700 conjugated antibody (Azure biosystems Cat# AC2128) – secondary antibody for Western blotting (1:10,000)<br>Goat anti-mouse AzureSpectra 550 conjugated antibody (Azure biosystems Cat# AC2159) – secondary antibody for Western blotting (1:10,000)
Validation	All primary and secondary antibodies used in this study were validated by their manufacturer for use in Western blotting. In our experiments, specific signal was observed at the expected size for targets in Western blot for all primary antibodies with consistent signal across replicates and reduced signal for target proteins following treatment with siRNA directed to targets transcript.

Eukaryotic cell lines

Policy information about cell lines and Sex and Gender in Research

Cell line source(s)	HepG2 – Originally sourced for ATCC for previous work
Authentication	HepG2 cells were originally sourced from an authenticated repository (ATCC). Cells were also validated using short tandem repeat (STR) at ATCC using their Cell Line Authentication Service.
Mycoplasma contamination	HepG2 cells were tested to confirm lines were negative for mycoplasma by the Preclinical Modeling Core Facility at the Koch Institute for Integrative Cancer Research.
Commonly misidentified lines<br>(See ICLAC register)	Not applicable

Plants

Seed stocks	Not applicable (plants were not used)
Novel plant genotypes	Not applicable (plants were not used)
Authentication	Not applicable (plants were not used)
